# Antibacterial and β-amyloid precursor protein-cleaving enzyme 1 inhibitory polyketides from the fungus *Aspergillus chevalieri*

**DOI:** 10.3389/fmicb.2022.1051281

**Published:** 2022-11-22

**Authors:** Qing-Yuan Wang, He-Ping Chen, Kai-Yue Wu, Xinyang Li, Ji-Kai Liu

**Affiliations:** ^1^School of Pharmaceutical Sciences, South-Central Minzu University, Wuhan, China; ^2^Graduate School of Pharmaceutical Sciences, The University of Tokyo, Bunkyo-ku, Japan

**Keywords:** *Aspergillus chevalieri*, polyketide, DP4+, antiproliferative activity, antibacterial activity, BACE1 inhibition

## Abstract

One new prenylated benzenoid, (±)-chevalieric acid (**1**), and four new anthraquinone derivatives, (10*S*,12*S*)-, (10*S*,12*R*)-, (10*R*,12*S*)-, and (10*R*,12*R*)-chevalierone (**2**–**5**), together with ten previously described compounds (**6**–**15**), were isolated from the fungus *Aspergillus chevalieri* (L. Mangin) Thom and Church. The structures of new compounds were elucidated by extensive 1D and 2D nuclear magnetic resonance (NMR), and HRESIMS spectroscopic analysis. The absolute configurations of **2**–**5** were determined by experimental and calculated electronic circular dichroism (ECD) and DP4+ analysis. Compound **10** showed weak cytotoxicity against human lung cancer cell line A549 with IC_50_ 39.68 μM. Compounds **2**–**5** exhibited antibacterial activities against the methicillin-resistant *Staphylococcus aureus* (MRSA) and opportunistic pathogenic bacterium *Pseudomonas aeruginosa*. The MIC value for compound **6** against MRSA is 44.02 μM. Additionally, Compounds **8**, **10**, **11** showed weak to moderate inhibitory activities against the β-secretase (BACE1), with IC_50_ values of 36.1, 40.9, 34.9 μM, respectively.

## Introduction

Dementia is a syndrome in which there is deterioration in cognitive function beyond the usual consequences of biological aging (World Health Organization, 2022). Dementia ranks the seventh leading cause of death and one of the major causes of disability among older people. Currently, more than 55 million people live with dementia worldwide, and a 10 million new cases increase every year. Alzheimer’s disease (AD) is the most common form of dementia which contributes to 60–70% of the cases. Current research indicate that amyloid β peptide (Aβ) aggregation is pathophysiological result of AD (Busche and Hyman, 2020; Piller, 2022). The Aβ protein is produced by cleavage of Aβ protein precursor by the β-secretase (BACE1) (Vassar and Kandalepas, 2011). However, in the field of AD drug development, more than 99% of clinical trials failed (Cummings et al., 2019). One of the main reasons is that lacking a potential lead compound reservoir which could feed the drug development process continuously. Additionally, human health is threatened by the emerging of “superbugs,” which show resistance to the antibiotics currently used (Rossiter et al., 2017). The pace for antibiotic drug development in recent years has slowed down due to the already widely used antibiotics with different mechanisms and lacking promising lead compounds. The rapid evolution of bacterial resistance to antibiotics urged us to keep the pace to find new types of lead compounds.

Fungal secondary metabolites are a natural reservoir which have contributed and still contributing to the development of drugs (Schueffler and Anke, 2014). *Aspergillus chevalieri* is a fungus belonging to the family *Aspergillaceae*. Fungi from this genus are widely distributed in the Dead Sea, desert, highlands, mangroves, or somewhere with high salt. Previous research on *Aspergillus* spp. led to the discovery of many diketopiperazine indole alkaloids (Zhong W.-M. et al., 2018), benzaldehyde derivatives (Zhong W.-M. et al., 2018; Zhong et al., 2020; Zhong W. et al., 2021), anthraquinone derivatives (Zhang et al., 2017; Zhao D. et al., 2018) and cochlioquinones (Koyama et al., 2005; Yamazaki et al., 2008). Most of the benzaldehyde derivatives displayed cytotoxic activity (Gao et al., 2012) and antioxidant activity (Miyake et al., 2009). In a follow-up program for mining bioactive natural products from fungi, the secondary metabolites of the fungus *A. chevalieri* were studied, which resulted in the isolation of five new and ten known compounds. We herein report the isolation, structural elucidation, and biological activities of the isolates in terms of cytotoxicity, antibacterial activity, and BACE1 inhibitory activity.

## Materials and methods

### General experimental procedures

UV spectra were recorded on a Hitachi UH5300 UV-Vis spectrophotometer (Hitachi, Co., Ltd., Tokyo, Japan). IR spectra were obtained on a Tracer-100 Fourier transform infrared spectrometer (Shimadzu Corporation, Tokyo, Japan) using KBr pellet. All nuclear magnetic resonance (NMR) spectra were recorded on a Bruker Ascend 600 MHz spectrometer (Bruker Corporation, Karlsruhe, Germany). HRESIMS were measured on a Q Exactive HF mass spectrometer (Thermo Fisher Scientific, Waltham, MA, USA). Column chromatography (CC) was performed on silica gel (Qingdao Haiyang Chemical Co., Ltd., Qingdao, China) and Sephadex LH-20 (Amersham Biosciences, Uppsala, Sweden). Medium pressure liquid chromatography (MPLC) was performed on a Biotage SP1 equipment, and column was packed with C_18_ silica gel. Preparative high performance liquid chromatography (prep-HPLC) was performed on an Agilent 1260 liquid chromatography system equipped with Agilent Zorbax SB-C_18_ columns (particle size, 5 μm, i.d. 9.4 mm × 150 mm or 21.2 mm × 150 mm) and a diode array detector (DAD) (Agilent Technologies, Santa Clara, CA, US). Fractions were monitored by DAD detector, or thin layer chromatography (TLC) (GF_254_, Qingdao Haiyang Chemical Co., Ltd., Qingdao, China). The chiral phase HPLC analysis and preparation were performed on Agilent 1200 liquid chromatography system with Daicel CHIRALPAK AD-H and CHIRALPAK AS-H columns (particle size 5 μm, i.d. 4.6 mm × 250 mm, flow rate 1 mL/min) (Daicel Corporation, Tokyo, Japan). 3-(4,5-Dimethylthiazol-2-yl)-2,5-diphenyltetrazolium bromide (MTT) and LY2811376 were purchased from Sigma-Aldrich (St. Louis, MO, USA). Penicillin G sodium salt and ceftazidime were purchased from Solarbio (Beijing, China). Dulbecco’s modified Eagle’s medium (DMEM) and fetal bovine serum were obtained from HyClone (Logan, UT, USA). BACE1 FRET assay kit, red (P2985) was bought from Thermo Fisher Scientific (MA, USA).

### Fungal material

*Aspergillus chevalieri* was isolated from the mud of the coast of Shenzhen Bay, China in May 2019, and was identified by ITS region sequencing. A voucher strain (No. HP-5) was stored at the School of Pharmaceutical Sciences, South-Central Minzu University, Wuhan, China. The fungus was fermented by cooked rice medium. The preparation of rice media was 100 g rice with 100 mL sea water each in 500-mL Erlenmeyer flasks. The fermentation was kept in dark environment for 40 days.

### Extraction and isolation

The rice cultures of *A. chevalieri* (15 kg) were collected and extracted with methanol at room temperature (100 L, 5 days each). The methanol extract was evaporated to dry under reduced pressure. The residue (2.1 kg) was resuspended in H_2_O and partitioned against ethyl acetate (EtOAc) for three times to give an EtOAc extract (226 g). The EtOAc extract was subjected to CC eluting with the solvent mixture petroleum ether-acetone (v/v 20:1, 10:1, 5:1, 2:1, 0:1) to yield four fractions (A–D).

Fraction A was eluted by MPLC with a stepwise gradient of MeOH-H_2_O (v/v 0:100–100:0) to afford subfractions A1-A8. Fraction A4 was applied to silica gel CC (petroleum ether–acetone from v/v 10:1 to 0:1) to yield five subfractions A4a-A4e.

The subfraction A4b was separated by prep-HPLC (MeCN-H_2_O = v/v 46:54–56:44 in 25 min, 4 mL min^–1^) to yield **7** (4.1 mg, t*_*R*_* = 14.5 min) and **8** (2.8 mg, t*_*R*_* = 18.0 min).

Fraction A5 was purified to silica gel CC (petroleum ether–acetone from v/v 10:1 to 0:1) and was further isolated by prep-HPLC (MeCN-H_2_O = v/v 37:63–47:53 in 25 min, 4 mL min^–1^) to yield compound **14** (3.5 mg, t*_*R*_* = 13.6 min) and compound **15** (4.2 mg, t*_*R*_* = 16.5 min). Fraction A6 was subjected to prep-HPLC (MeCN-H_2_O = v/v 35:65–55:45 for 25 min, 4 mL min^–1^) to give compound **1** (2.8 mg, t*_*R*_* = 15.2 min).

Fraction B was purified by prep-HPLC (MeCN-H_2_O = v/v 47:53–57:43 for 25 min, 4 mL min^–1^) to yield the compound mixture **2**–**5** (3.6 mg, t*_*R*_* = 21.5 min). Then the compounds **2** and **3** were purified by semi-preparative HPLC with CHIRALPAK AS-H (*n*-hexane/2-propanol = v/v 97:3, isocratic, 1 mL min^–1^) to yield **2** (0.9 mg, t*_*R*_* = 20.0 min), **3** (0.7 mg, t*_*R*_* = 27.3 min) and compound mixture **4**/**5** (1.8 mg, t*_*R*_* = 17.0 min). The mixture **4**/**5** were further applied to semi-preparative HPLC with CHIRALPAK AD-H (*n*-hexane/2-propanol = v/v 94:6 isocratic, 1 mL min^–1^) to yield **4** (0.7 mg, t*_*R*_* = 13.2 min), **5** (0.6 mg, t*_*R*_* = 16.1 min).

Fraction D was fractionated by silica gel CC (CHCl_3_-MeOH from v/v 40:1, 30:1, 20:1, 10:1, 0:1) to give four subfractions D1–D3. Then subfraction D2 was isolated by prep-HPLC (MeCN-H_2_O = 49:51, 4 mL min^–1^, 25 min) to yield **9** (3.4 mg, t*_*R*_* = 17.5 min) and **10** (6.2 mg, t*_*R*_* = 18.6 min). Fraction D3 was subject to silica gel CC (petroleum ether–acetone from v/v 10: 1 to 0: 1) and further applied to prep-HPLC (MeCN-H_2_O = 43:57–63:37 in 25 min, 4 mL min^–1^) to yield **11** (6.2 mg, t*_*R*_* = 15.2 min) and **12** (4.3 mg, t*_*R*_* = 18.7 min).

Fraction E was separated by Sephadex LH-20 (MeOH), and further purified by prep-HPLC (MeCN-H_2_O = 53:47 in 25 min, 3 mL min^–1^) to yield **6** (2.4 mg, t*_*R*_* = 17.3 min), and **13** (2.6 mg, t*_*R*_* = 21.5 min).

### Spectroscopic data

(±)-Chevalieric acid (**1**): yellow amorphous solid; UV (MeOH) λ_max_ (log ε): 210 (3.01) nm; [α]_D_^24^ + 2.7 (*c* 0.05, MeOH); ^1^H and ^13^C NMR data, Table 1; HRESIMS *m/z* 319.19028 [M+H]^+^, calcd. for C_19_H_27_O_4_, 319.19093.

**TABLE 1:** ^1^H and ^13^C nuclear magnetic resonance (NMR) spectroscopic data of compounds 1–5 (600/150 MHz, CDCl_3_).

No.	1		2/4		3/5	
1	170.6, C		165.7, C		165.5, C	
2	108.0, C		99.6, CH	6.39, d (2.2)	99.4, CH	6.39, d (1.8)
3	154.5, C		165.2, C		165.4, C	
4	129.2, C		108.1, CH	6.50, d (2.2)	108.5, CH	6.31, d (1.8)
4a			147.0, C		145.8, C	
5	124.0, CH	6.86, s	120.9, CH	6.57, s	120.9, CH	6.71, s
6	143.3, C		147.0, C		147.4, C	
7	121.5, C		116.6, CH	6.71, s	116.4, CH	6.76, s
8	26.8, CH_2_	2.65, dd (16.7, 11.8) 3.08, dd (16.7, 3.5)	162.5, C		162.3, C	
8a			114.4, C		114.2, C	
9	80.0, CH	4.51, m	191.8, C		191.8, C	
9a			110.6, C		110.6, C	
10	35.0, CH_2_	1.73, m, 1.88, m	47.8, CH	4.06, d (3.4)	47.6, CH	4.07, d (2.3)
10a			143.8, C		145.0, C	
11	24.7, CH_2_	1.47, m 1.56, m	22.4, CH_3_	2.38, s	22.4, CH_3_	2.38, s
12	31.7, CH_2_	1.33, m	40.2, CH	2.49, m	40.2, CH	2.50, overlapped
13	22.7, CH_2_	1.33, m	46.7, CH_2_	2.02, dd (17.4, 7.3) 2.37, dd (17.4, 5.8)	47.0, CH_2_	2.10, overlapped 2.47, overlapped
14	14.1, CH_3_	0.90, t (7.0)	207.9, C		207.9, C	
15	27.6, CH_2_	3.31, d (7.6)	30.8, CH_3_	2.09, s	30.9, CH_3_	2.12, s
16	121.3, CH	5.28, br. t (7.6)	16.4, CH_3_	0.69, d (7.0)	16.4, CH_3_	0.62, d (6.7)
17	134.0, C					
18	17.9, CH_3_	1.69, s				
19	26.0, CH_3_	1.75, s				
1-OCH_3_			55.8, CH_3_	3.87, s	55.8, CH_3_	3.87, s
2-OH				12.50, s		12.55, s
7-OH		10.89, s				
11-OH				12.20, s		12.17, s

(10*S*,12*S*)-Chevalierone (**2**): yellow powder; UV (MeOH) λ_max_ (log ε): 230 (3.40), 270.0 (3.17), 340.0 (3.46) nm; IR (KBr) ν_max_ 3429, 2962, 2927, 1712, 1620, 1600, 1288, 1103, 817 cm^–1^. [α]_D_^24^ + 30.7 (*c* 0.07, MeOH); ^1^H and ^13^C NMR data, Table 1; HRESIMS *m/z* 355.15402 [M+H]^+^, calcd. for C_21_H_23_O_5_, 355.15455.

(10*S*,12*R*)-Chevalierone (**3**): yellow powder; UV (MeOH) λ_max_ (log ε): 220.0 (3.29), 275.0 (2.89), 360.0 (3.22) nm; [α]_D_^24^ + 30.39 (*c* 0.05, MeOH); ^1^H and ^13^C NMR data, Table 1; HRESIMS *m/z* 355.15405 [M+H]^+^, calcd. for C_21_H_23_O_5_, 355.15455.

(10*R*,12*R*)-Chevalierone (**4**): yellow powder; UV (MeOH) λ_max_ (log ε): 230.0 (3.38), 270.0 (3.13), 350.0 (3.45) nm; [α]_D_^24^ −33.10 (*c* 0.05, MeOH); ^1^H and ^13^C NMR data, Table 1; HRESIMS *m/z* 355.15402 [M+H]^+^, cacld. for C_21_H_23_O_5_, 355.15455.

(10*R*,12*S*)-Chevalierone (**5**): yellow powder; UV (MeOH) λ_max_ (log ε): 230.0 (3.42) 275.0 (3.32) 340.0 (3.49) nm; [α]_D_^24^ −33.14 (*c* 0.06, MeOH); ^1^H and ^13^C NMR data, Table 1; HRESIMS *m/z* 355.15393 [M+H]^+^, calcd. for C_21_H_23_O_5_, 355.15455.

### Electronic circular dichroism and nuclear magnetic resonance calculations

Conformational analyses of the candidate structures were performed using the MMFF94s force field. For ECD calculation, the conformers with a distribution higher than 1% were further optimized at B3LYP/6-31G(d) level of theory in the Gaussian 16 software package (Frisch et al., 2016). The conformers within 3 kcal/mol energy of global minimum were subjected to theoretical calculation of ECD using time-dependent density functional theory (TDDFT) at B3LYP/6-31G(d,p) level in methanol with IEFPCM model. The calculated and weighted ECD curves were all generated using SpecDis 1.71 (Bruhn et al., 2013, 2017), respectively, and plotted in Microsoft Office Excel Program. For NMR calculation, the conformers with a distribution higher than 1% were further optimized at B3LYP/6-311G(2d,p) level of theory in Gaussian 16 software package. The conformers within 3 kcal/mol energy of global minimum were subjected to theoretical calculation of ECD using TDDFT at B3LYP/6-311G(2d,p) level in chloroform with IEFPCM model. The calculated data were processed with an in-house Office Excel Workbook.

### DP4+ calculation and analysis

The conformations of compounds **2**–**5** from ECD calculations were further optimized at the B3LYP/6-311G(2d,p) level. With these optimized conformers, chemical shift values at mPW1PW91/6-31 + G(d,p) for DP4+ for conformational analysis were computed in the solvent phase with chloroform. The averaged shielding tensors were used for probabilities calculation with the spreadsheet provided by the original publication (Grimblat et al., 2015).

### Cytotoxicity assays

The human lung cancer cell line A549 used in this study was purchased from Conservation Genetics CAS Kunming Cell Bank. The cells were cultured in high-glucose DMEM with 10% fetal bovine serum, 100 mg/mL streptomycin, 100 U/mL penicillin, and kept in an incubator at 37°C under an atmosphere of 5% CO_2_. The cell viability was assessed by 3-(4,5-dimethylthiazol-2-yl)-2,5-diphenyltetrazolium bromide (MTT) method. The presence of NAD(P)H-dependent cellular oxidoreductases reflects the number of viable cells. These enzymes are capable of reducing MTT to its insoluble formazan, which has a purple color. Therefore, colorimetric measurements of the amount of insoluble formazan which produced in living cells based on the reduction of MTT can evaluate the cell viability. In brief, 100 μL of adherent cells was seeded to each well of a 96-well cell culture plate. The plate was kept for 12 h for adherence. The DMSO solution of test compounds were added to each well to give a final compound concentration of 40 μM. However, suspended cells were seeded with the same density of 1 × 10^5^ cells/mL every 100 μL of culture medium before adding the test compounds. Then the plate was incubated for another 48 h. After 48 h of incubation, 100 μg of MTT was added to each well, another 4 h of incubation was continued at 37°C. After removal of 100-μL culture medium, the cells were lysed with 20% SDS-50% DMF (100 μL). The remained lysates were subjected to measure the optical density value at 595 nm with a 96-well microtiter plate reader (Tecan Spark 10M, Switzerland). Cisplatin was used as positive control. All the experiments were in triplicates. The IC_50_ value was calculated by a published method (Chen et al., 2018).

### Antibacterial assays

In this study, the tested bacteria strains, *Escherichia coli* ATCC25922, *Staphylococcus aureus* subsp. *aureus* ATCC29213, *Salmonella enterica* subsp. *enterica* ATCC14028, *Pseudomonas aeruginosa* ATCC27853, methicillin-resistant *S. aureus* (MRSA) were purchased from China General Microbiological Culture Collection Center (CGMCC). All the strains were cultured in Mueller-Hinton broth (Oxoid, Thermo Fisher Scientific) at 37°C with shaking at 200 rpm overnight. Each culture was diluted 40-fold in fresh MHB broth and incubated at 37°C with shaking (200 rpm) for another 2–3 h. The mid-log phase cultures were diluted to a concentration of 5 × 10^5^ CFU/mL by McFarland Standards, which was used as seeding solution for following antibacterial assays. Each well of the compound-containing 96-well plates was seeded with 100 μL of aforementioned seeding solution, giving a final compound concentration 200 μM. Plates were covered and incubated at 37°C for 24 h. The inhibitory rates were calculated by [(1-OD_625sample_)/OD_625negative control_] × 100%. Penicillin G sodium and ceftazidime were used as positive controls (inhibitory rates >99%) (Zhao Z.-Z. et al., 2018). Compounds were first tested for their potential against five bacterial strains at a single concentration of 200 μM. If the inhibitory rate above 50%, different concentrations of the compounds were further evaluated for the antibacterial ability.

### β-amyloid precursor protein-cleaving enzyme 1 FRET assays

β-amyloid precursor protein-cleaving enzyme 1 (β-secretase, BACE1) assays were performed according to the literature (Qi et al., 2018; Yatsu et al., 2019). First, 10 μL of the test sample, 10 μL of the 722 nM BACE1 substrate (Rh-EVNNLDAEFK-Quencher in 50 mM ammonium bicarbonate), and 10 μL of the BACE1 enzyme (1 U/mL) were mixed in black 384-well plates and incubated for 3 h at room temperature. The fluorescence intensity was measured at 540 nm excitation and 590 nm emission using a microplate reader (Tecan Spark 10M, TECAN, Männedorf, Switzerland) at the time points of 1 h and 3 h. The BACE1 inhibition rate was calculated by the following equation: BACE1 inhibition rate (%) = {1–[(S–S_0_)–(B–B_0_)/(C–C_0_)–(B–B_0_)]} × 100, where C is the fluorescence of a DMSO control (enzyme, substrate, and assay buffer with DMSO) after 3 h of incubation, C_0_ is the fluorescence of the DMSO control at 1 h after incubation, B is the fluorescence of a non-enzyme control (substrate and assay buffer with DMSO) after 3 h of incubation, B_0_ is the fluorescence of the non-enzyme control at 1 h after incubation, S is the fluorescence of the tested samples (enzyme, sample solution, and substrate) after 3 h of incubation, and S_0_ is the fluorescence of the tested samples at 1 h after incubation. The IC_50_ values were calculated by GraphPad Prism software (v5.02, CA, USA).

## Results and discussion

### Structural elucidation

**FIGURE 1:**
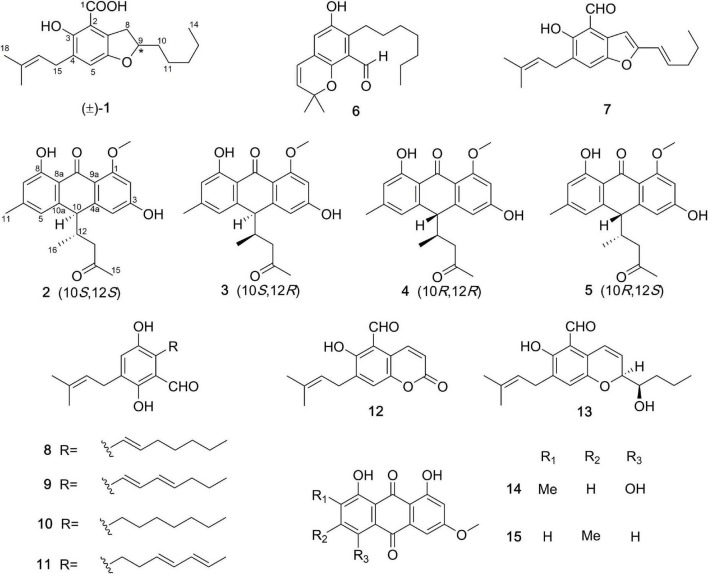
The chemical structures of compounds **1**–**15**.

**FIGURE 2:**
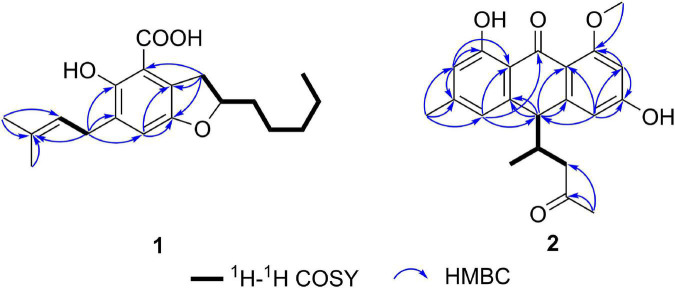
Key HMBC and ^1^H-^1^H COSY correlations of **1** and **2**.

Compound **1** (Figure 1) was obtained as a racemate which was confirmed by chiral phase HPLC analysis. The HRESIMS spectroscopic analysis at *m/z* 319.19028 [M+H]^+^ (calcd. for C_19_H_27_O_4_, 319.19093) revealed the molecular formula is C_19_H_26_O_4_. According to the 1D NMR spectroscopic data of **1** (Table 1), two olefinic protons at δ_H_ 6.86 (1H, s, H-5) and at δ_H_ 5.28 (1H, br. t, *J* = 7.6 Hz, H-16), one methine at δ_H_ 4.51 (1H, m, H-9), three methyl groups at δ_H_ 0.90 (3H, t, *J* = 7.0 Hz, H_3_-14), 1.69 (3H, s, H_3_-18), 1.75 (3H, s, H_3_-19) were identified. The ^13^C NMR and HSQC spectra revealed one carbonyl carbon at δ_C_ 170.6 (C-1), six non-protonated carbons at δ_C_ 108.0 (C-2), 121.5 (C-7), 143.3 (C-6), 129.2 (C-4), 154.5 (C-3), 134.0 (C-17), three methines at δ_C_ 124.0 (C-5), 80.0 (C-9), 121.3 (C-16), six methylene carbons at δ_C_ 26.8 (C-8), 35.0 (C-10), 24.7 (C-11), 31.7 (C-12), 22.7 (C-13), 27.6 (C-15) and three methyl carbons at δ_C_ 14.1 (C-14), 17.9 (C-18), 26.0 (C-19). The data showed high similarity to those of 2-(2′,3-epoxy-1′,3′-heptadienyl)-6-hydroxy-5-(3-methyl-2-butenyl)benzaldehyde (**7**) (Wang et al., 2006), a previously described compound which was also been encountered in this study. The ^1^H-^1^H COSY correlations of H_2_-8, H-9, H_2_-10, H_2_-11, and the HMBC correlation from H_2_-8 to C-2, C-6, C-7, and from H-9 to C-6, together with the ^13^C chemical shifts of C-6 and C-9 suggested that the absence of conjugated double bonds at C-8, C-9, C-10, and C-11 in **1** compared to those of **7** (Figure 2).

Additionally, the ^13^C chemical shifts of C-1 (δ_C_ 170.6), as well as the absence of the aldehyde proton, indicated that C-1 in **1** was a carboxylic group instead of being an aldehyde carbonyl in **7**. This change was also in accordance with the HRESIMS data. Considering that this isolate only has one chiral carbon, the question about whether compound **1** was optically pure was raised (Chen et al., 2016). Although the chiral phase HPLC analysis of this sample on two kinds of chiral phase columns, the CHIRALPAK AS-H and AD-H showed only one peak, suggesting either the sample was optically pure or the chiral phase columns used were unable to separate the antipodes. However, the small specific optical rotation value (+2.7) and the reported analog in the literature (Zhong W.-M. et al., 2018; Zhong W. et al., 2021) indicated that this sample is a racemic mixture. Hence, compound **1** was named chevalieric acid.

**FIGURE 3:**
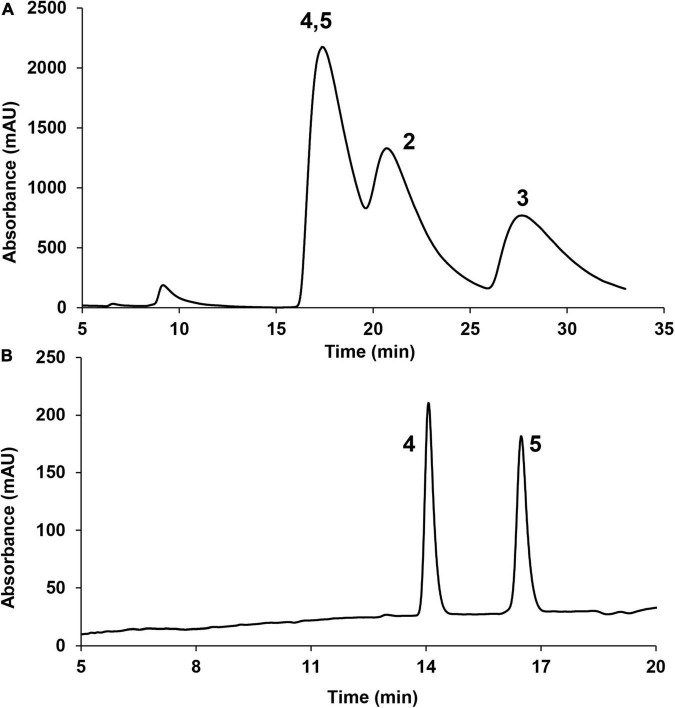
Chiral phase HPLC analysis of compounds **2**–**5** (220 nm). **(A)** CHIRALPAK AS-H column; **(B)** CHIRALPAK AD-H column.

In this experiment, a chromatograph peak by reverse phase HPLC analysis on the C_18_ column was obtained, of which the purity met the requirement for further NMR analysis. However, the ^1^H NMR spectrum exhibited two sets of proton signals with a ratio 1:1 (Supplementary Figure 16), indicating the presence of two stereoisomers of this sample. Chiral phase HPLC analysis (column: Daicel CHIRALPAK AS-H) of this sample gave three peaks (Figure 3A), which were further subjected to measuring the ^1^H NMR spectra. The results suggested that the latter two peaks which were designated as compounds **2** and **3** were optically pure; however, the first peak was still a mixture. Further chiral phase HPLC analysis (column Daicel CHIRALPAK AD-H) of this mixture presented two isolated peaks which were designated as compounds **4** and **5** (Figure 3B). The clean NMR spectra of the optically pure isolates helped to unambiguously determine their structures by NMR spectroscopic analysis, specifical optical rotation measurements, and computational methods.

The NMR spectra between compounds **2** and **4**, compounds **3** and **5** are identical, indicating that **2**/**4**, **3**/**5** are two pairs of enantiomers. The HRESIMS spectroscopic analysis of **2**/**4** gave the protonated ion peak at *m/z* 355.15402 [M+H]^+^, revealing the molecular formula of C_21_H_22_O_5_ (calcd. for C_21_H_23_O_5_, 355.15455). The ^1^H NMR spectroscopic data (Table 1) of **2**/**4** showed four aromatic protons at δ_H_ 6.39 (d, *J* = 2.2 Hz, H-2), 6.50 (d, *J* = 2.2 Hz, H-4), 6.57 (s, H-5), 6.71 (s, H-7), two methine protons at δ_H_ 4.06 (d, *J* = 3.4 Hz, H-10), 2.49 (m, H-12), one methylene group at δ_H_ 2.02 (dd, *J* = 17.4, 7.3 Hz, H-13a), 2.37 (dd, *J* = 17.4, 5.8 Hz, H-13b), four methyl groups at δ_H_ 2.38 (s, H-11), 2.09 (s, H-15), 0.69 (d, *J* = 7.0 Hz, H-16), 3.87 (s, 1-OCH_3_). The ^13^C NMR data (Table 1) and HSQC spectra indicated a total of 21 carbon signals, including two carbonyl carbons at δ_C_ 191.8 (C-9), 207.9 (C-14), twelve aromatic carbons at δ_C_ 165.7 (C-1), 99.6 (C-2), 165.2 (C-3), 108.1 (C-4), 147.0 (C-4a), 120.9 (C-5), 147.0 (C-6), 116.6 (C-7), 162.5 (C-8), 114.4 (C-8a), 110.6 (C-9a), 143.8 (C-10a), two methine carbons at δ_C_ 47.8 (C-10), 40.2 (C-12), one methylene carbon at δ_C_ 46.7 (C-13), four methyl carbons at δ_C_ 22.4 (C-11), 30.8 (C-15), 16.4 (C-16), 55.8 (1-OCH_3_). The HMBC correlations from H-2 to C-1, C-3, C-9a, C-4, from H-4 to C-2, C-3, C-4a, C-9a, C-10, from H-5 to C-6, C-10, C-11, C-7, C-8a, and from H-7 to C-5, C-8, C-8a, C-11 (Figure 2) suggested the presence of an anthraquinone skeleton in compounds **2**/**4**. The ^1^H-^1^H COSY correlations of H-10/H-12/H_2_-13, H-12/H_3_-16 in combination with the HMBC correlation from H_3_-15 to the carbonyl at δ_C_ 207.9 (C-14), and C-13 indicated that an additional chain with five carbons attached to the anthraquinone skeleton at the position C-10. The HMBC correlation from δ_H_ 3.87 to C-1 showed that C-1 was substituted by a methoxy group. According to the analysis above, the planar structure of **2**/**4** was determined as shown in Figure 2. Structural elucidation of the NMR spectra of **3**/**5** showed that the two compounds shared the same planar structures with compounds **2** and **4**.

**FIGURE 4:**
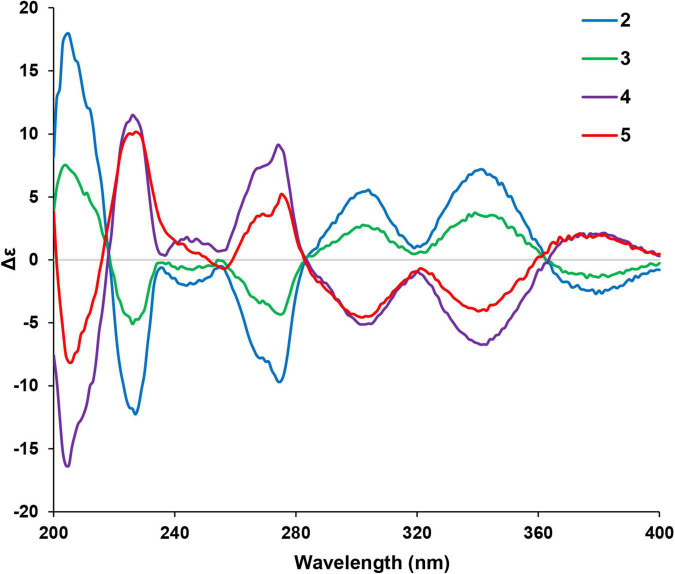
Experimental CD spectra of compounds **2**–**5**.

Given that there are two chiral centers in the planar structures of compounds **2**–**5** which correspond to four stereoisomers, including two pairs of enantiomers, the 10*S*,12*S*, the 10*R*,12*R*, the 10*S*,12*R*, and the 10*R*,12*S*. The absolute configurations of the four congeners were determined by computational methods. The experimental circular dichroism (CD) spectra of the four compounds were obviously classified into two groups which mirrors to each other, i.e., compounds **2** and **3** mirror with compounds **4** and **5**, respectively, which was in accordance with the NMR data. Therefore, to simplify the calculation work, only compounds **2** and **3** were discussed hereinafter. First of all, the absolute configurations of **2** and **3** were unable to be determined by ECD calculations because of the remarkable similarity between the experimental CD spectra of these two compounds (Figure 4).

**FIGURE 5:**
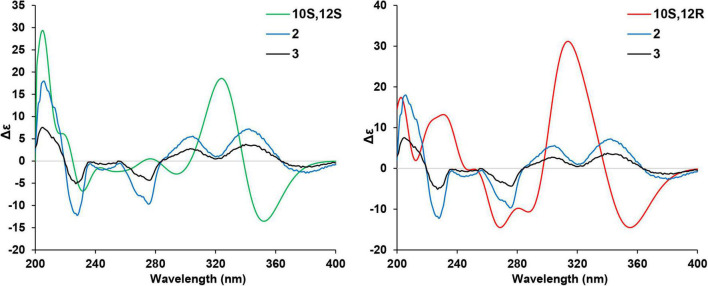
Comparison of the calculated electronic circular dichroism (ECD) of (10*S*,12*S*)- and (10*S*,12*R*)-isomers vs. compounds **2** and **3**.

The two theoretical epimers with the configurations 10*S*,12*S* and 10*S*,12*R* were subjected to ECD calculations. As a result, the comparison between the calculated ECD and experimental CD failed to assign the absolute configuration of compounds **2** and **3** due to the close absorption patterns of the experimental CD of the two compounds (Figure 5).

**FIGURE 6:**
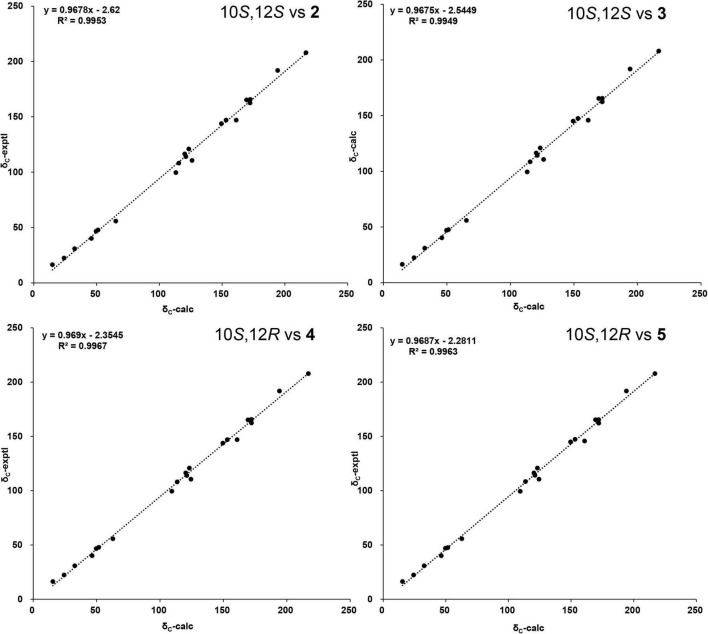
The linear regression analysis of calculated and experimental nuclear magnetic resonance (NMR) data of (10,12)-stereoisomers and compounds **2** and **3**.

The two stereoisomers were further subjected to ^13^C NMR calculations at the B3LYP/6-311G(2d,p) level of theory with IEFPCM model in chloroform based on the optimized geometries at B3LYP/6-311G(2d,p) level of theory. As shown in Figure 6, all the linear regression analyses between the two theoretical epimers with compounds **2** and **3** gave good results since the close similarity of the ^13^C NMR between compounds **2** and **3**. Therefore, this evidence was unable to assign the absolute configurations of compounds **2** and **3** unambiguously.

The DP4+ method is one of the leading strategies with reliable performance in structural assignment of compounds based on the Bayesian analysis of the calculated NMR data (Grimblat et al., 2015; Marcarino et al., 2021). The NMR shielding values of the (10,12)-stereoisomers were calculated with the recommended level of theory [mPW1PW91/6-31G + (d,p)//B3LYP/6-31G(d)], the calculated results were subjected to DP4+ analysis against the experimental chemical shifts of compounds **2** and **3**, respectively. As shown in Table 2, the DP4+ analyses of all data (^13^C and ^1^H shielding values) suggested that compound **2** preferred the 10*S*,12*S* configuration instead of the 10*S*,12*R* configuration (98.01–1.99), and compound **3** preferred the 10*S*,12*R* configuration instead of the 10*S*,12*S* configuration (99.84–0.16), allowing the unambiguously assign the absolute configuration of compounds **2** and **3** as 10*S*,12*S*, and 10*S*,12*R*, respectively. Taking in consideration of the specific optical values and NMR results, the absolute configurations of compounds **4** and **5** were, thereby, determined as 10*R*,12*R*, and 10*R*,12*S*, respectively.

**TABLE 2:** DP4+ analysis of the (10,12)-stereoisomers with compounds **2** and **3** (sDP4+: scaled chemical shifts; uDP4+: unscaled chemical shifts).

Item	2		3	
	10*S*,12*S*	10*S*,12*R*	10*S*,12*S*	10*S*,12*R*
sDP4+ (H data)	92.23%	7.77%	41.70%	58.30%
sDP4+ (C data)	80.11%	19.89%	27.01%	72.99%
sDP4+ (all data)	97.95%	2.05%	20.93%	79.07%
uDP4+ (H data)	33.92%	66.08%	2.37%	97.63%
uDP4+ (C data)	66.79%	33.21%	20.14%	79.86%
uDP4+ (all data)	50.80%	49.20%	0.61%	99.39%
DP4+ (H data)	85.89%	14.11%	1.71%	98.29%
DP4+ (C data)	89.01%	10.99%	8.54%	91.46%
DP4+ (all data)	**98.01**%	**1.99%**	**0.16%**	**99.84%**

The bold values indicate the values of greatest importance.

In this study, ten previously reported compounds were obtained, asperglaucin B (**6**), the 2-(2′,3-epoxy-1′,3′-heptadienyl)-6-hydroxy-5-(3-methyl-2-butenyl)benzalde-hyde (**7**) (Chen et al., 2016), tetrahydroauroglaucin (**8**) (Smetanina et al., 2007), dihydroauroglaucin (**9**) (Miyake et al., 2009), flavoglaucin (**10**) (Yoshihira et al., 1972), isodihydroauroglaucin (**11**) (Miyake et al., 2009), cristaldehyde A (**12**) (Zhang et al., 2019), (3′*S*,4′*R*)-6-(3′,5-epoxy-4′-hydroxy-1′-heptenyl)-2-hyd roxy-3-(3″-methyl-2″-butenyl)benzaldehyde (**13**) (Chen et al., 2017), 1,4,5-trihydroxy-7-methoxy-2-methyl-anthraquinone (**14**) (Awaad et al., 2014), physcion (**15**) (Yoshihira et al., 1972). All the isolated polyketides were classified into two groups, the salicylaldehyde derivatives with prenyl group modifications, and the anthraquinones. Compounds **2**–**5** are unusual anthraquinones with additional side chains, which worth to be studied in terms of biosynthetic origins.

### Biological activity evaluation

All the benzaldehyde derivatives were subjected to screening the cytotoxicity against the human lung cancer line A549, only compound **10** showed weak cytotoxicity activity against the human lung cancer line A549 with the IC_50_ 39.68 μM (positive control cisplatin, IC_50_ 5.03 μM).

The Aβ protein, produced by the β-amyloid precursor protein-cleaving enzyme 1, is considered as the pathophysiology product of AD. We evaluated the BACE1 inhibitory of all benzaldehyde derivatives compounds isolated in this study. Compounds **8**, **10**, and **11** showed moderate to weak inhibition against the β-secretase with the IC_50_ values of 36.1, 40.9, 34.9 μM, respectively (Table 3). The IC_50_ value for the positive control LY2811376 was 0.6 μM. The results indicated the potential of salicylaldehydes serving as template structures for further chemical modifications in the field of anti-AD drug research and development.

**TABLE 3:** β-amyloid precursor protein-cleaving enzyme 1 (BACE1) inhibitory tests of compounds **1** and **6**–**13**.

Sample	1	6	7	8	9	10	11	12	13	LY281 11376
IC_50_ (μM)	100.7	>100	>100	36.1	>100	40.9	34.9	>100	>100	0.6

All the anthraquinone derivatives (**2**–**5**, **14**–**15**) were evaluated for the antibacterial activity against five bacteria, the *Escherichia coli* ATCC25922, *S. aureus* subsp. *aureus* ATCC29213, *S. enterica* subsp. *enterica* ATCC14028, *P. aeruginosa* ATCC27853, and methicillin-resistant *S. aureus* (Table 4). Compounds **3**–**5** showed significant inhibition against the opportunistic pathogenic bacterium *P. aeruginosa* (inhibition rate: 81.0–91.5%) and methicillin-resistant *S. aureus* (inhibition rate: 74.0–88.5%) at the concentration of 200 μM, while the structural congener compound **2** only showed weak inhibition (inhibition rate: 38.2%) against the *P. aeruginosa* at 200 μM. Due to the shortage of samples, we only measured the inhibitory activity against MRSA of compound **5** with the MIC_50_ value of 44.02 μM.

**TABLE 4:** Inhibition rates of anthraquinone derivatives against the bacteria at the concentration of 200 μM.

Sample	*Escherichia coli*	*Staphylococcus aureus* subsp. *aureus*	*Salmonella enterica* subsp. *enterica*	*Pseudomonas aeruginosa*	Methicillin-resistant *Staphylococcus aureus*
Penicillin G sodium	–^a^	–	75.7%	99.9%	60.5%
Ceftazidime	99.8%	–	99.3%	–	–
2	–	–	–	38.2%	–
3	–	–	–	81.9%	74.1%
4	–	–	–	81.0%	85.0%
5	–	–	–	91.5%	88.5%
14	–	–	–	38.2%	52.0%
15	–	–	–	38.0%	–

^a^This symbol “–” represent no activity.

## Conclusion

In summary, five previously undescribed polyketide derivatives with ten known compounds were isolated from the fungus *A. chevalieri*. Through comprehensive analysis of 1D, 2D NMR spectroscopic data, ECD calculations, ^13^C NMR calculations, and DP4+ analysis, the structures, including the absolute configurations of the new compounds, were unambiguously determined. In the bioassays, compound **10** showed weak cytotoxicity activity against the human lung cancer line A549 with the IC_50_ 39.68 μM. Compounds **8**, **10**, and **11** exhibited moderate BACE1 inhibitory activities with IC_50_ values of 36.1, 40.9, 34.9 μM, respectively. Compounds **3**, **4**, and **5** showed significant antibacterial activities against both *P. aeruginosa* and MRSA. Notably, the four stereoisomers **2**–**5** displayed different antibacterial activities, raising the question about stereochemistry-bioactivity-relationship, which need to be addressed in the future. This study provides new horizons for follow-up drug development for anti-MRSA agents.

## Data availability statement

The original contributions presented in this study are included in the article/Supplementary material, further inquiries can be directed to the corresponding authors.

## Author contributions

XL and J-KL designed and supervised the project. Q-YW and H-PC performed the experiments. All authors analyzed data, discussed the results, wrote the manuscript, and read and agreed to the published version of the manuscript.

## References

[B1] Awaad A. S. Al-Zaylaee H. M. Alqasoumi S. I. Zain M. E. Aloyan E. M. Alafeefy A. M. (2014). Anti-leishmanial activities of extracts and isolated compounds from *Drechslera rostrata* and *Eurotium tonpholium*. *Phytother. Res.* 28 774–780. 10.1002/ptr.5096 24375822

[B2] Bruhn T. Schaumlöffel A. Hemberger Y. Bringmann G. (2013). SpecDis: Quantifying the comparison of calculated and experimental electronic circular dichroism spectra. *Chirality* 25 243–249. 10.1002/chir.22138 23532998

[B3] Bruhn T. Schaumlöffel A. Hemberger Y. Pescitelli G. (2017). *SpecDis version 1.71, Berlin, Germany.* Available online at: https://specdis-software.jimdofree.com (accessed November 12, 2022).

[B4] Busche M. A. Hyman B. T. (2020). Synergy between amyloid-β and tau in Alzheimer’s disease. *Nat. Neurosci.* 23 1183–1193. 10.1038/s41593-020-0687-6 32778792 PMC11831977

[B5] Chen H.-P. Zhao Z.-Z. Li Z.-H. Huang Y. Zhang S.-B. Tang Y. (2018). Anti-proliferative and anti-inflammatory lanostane triterpenoids from the polish edible mushroom *Macrolepiota procera*. *J. Agric. Food Chem.* 66 3146–3154. 10.1021/acs.jafc.8b00287 29510036

[B6] Chen H.-P. Zhao Z.-Z. Zhang Y. Bai X. Zhang L. Liu J.-K. (2016). (+)- and (–)-Ganodilactone, a pair of meroterpenoid dimers with pancreatic lipase inhibitory activities from the macromycete *Ganoderma leucocontextum*. *RSC Adv.* 6 64469–64473. 10.1039/C6RA10638B

[B7] Chen M. Zhao Q. Hao J.-D. Wang C.-Y. (2017). Two benzaldehyde derivatives and their artefacts from a gorgonian-derived *Eurotium* sp. fungus. *Nat. Prod. Res.* 31 268–274. 10.1080/14786419.2016.1230116 27627699

[B8] Cummings J. Feldman H. H. Scheltens P. (2019). The “rights” of precision drug development for Alzheimer’s disease. *Alz. Res. Ther.* 11:76. 10.1186/s13195-019-0529-5 31470905 PMC6717388

[B9] Frisch M. J. Trucks G. W. Schlegel H. B. Scuseria G. E. Robb M. A. Cheeseman J. R. (2016). *Gaussian 16, Revision C.01.* Wallingford CT: Gaussian, Inc.

[B10] Gao J. Radwan M. M. León F. Wang X. Jacob M. R. Tekwani B. L. (2012). Antimicrobial and antiprotozoal activities of secondary metabolites from the fungus *Eurotium repens*. *Med. Chem. Res.* 21 3080–3086. 10.1007/s00044-011-9798-7 23024574 PMC3457657

[B11] Grimblat N. Zanardi M. M. Sarotti A. M. (2015). Beyond DP4: An improved probability for the stereochemical assignment of isomeric compounds using quantum chemical calculations of NMR shifts. *J. Org. Chem.* 80 12526–12534. 10.1021/acs.joc.5b02396 26580165

[B12] Koyama N. Nagahiro T. Yamaguchi Y. Masuma R. Tomoda H. Ōmura S. (2005). Stemphones, novel potentiators of imipenem activity against methicillin-resistant *Staphylococcus aureus*, Produced by *Aspergillus* sp. FKI-2136. *J. Antibiot.* 58 695–703. 10.1038/ja.2005.95 16466023

[B13] Marcarino M. O. Cicetti S. Zanardi M. M. Sarotti A. M. (2021). A critical review on the use of DP4+ in the structural elucidation of natural products: The good, the bad and the ugly. A practical guide. *Nat. Prod. Rep.* 39 58–76. 10.1039/D1NP00030F 34212963

[B14] Miyake Y. Ito C. Itoigawa M. Osawa T. (2009). Antioxidants produced by *Eurotium herbariorum* of filamentous fungi used for the manufacture of karebushi, dried bonito (katsuobushi). *Biosci. Biotechnol. Biochem.* 73 1323–1327. 10.1271/bbb.80887 19502740

[B15] Piller C. (2022). Blots on a field? *Science* 377 358–363. 10.1126/science.add9993 35862524

[B16] Qi C. Liu M. Zhou Q. Gao W. Chen C. Lai Y. (2018). BACE1 Inhibitory meroterpenoids from *Aspergillus terreus*. *J. Nat. Prod.* 81 1937–1945. 10.1021/acs.jnatprod.7b01050 30207465

[B17] Rossiter S. E. Fletcher M. H. Wuest W. M. (2017). Natural products as platforms to overcome antibiotic resistance. *Chem. Rev.* 117 12415–12474. 10.1021/acs.chemrev.7b00283 28953368 PMC5869711

[B18] Schueffler A. Anke T. (2014). Fungal natural products in research and development. *Nat. Prod. Rep.* 31 1425–1448. 10.1039/C4NP00060A 25122538

[B19] Smetanina O. F. Kalinovskii A. I. Khudyakova Y. V. Slinkina N. N. Pivkin M. V. Kuznetsova T. A. (2007). Metabolites from the marine fungus *Eurotium repens*. *Chem. Nat. Compd.* 43 395–398. 10.1007/s10600-007-0147-5

[B20] Vassar R. Kandalepas P. C. (2011). The β-secretase enzyme BACE1 as a therapeutic target for Alzheimer’s disease. *Alz. Res. Ther.* 3:20. 10.1186/alzrt82 21639952 PMC3226309

[B21] Wang S. Li X.-M. Teuscher F. Li D.-L. Diesel A. Ebel R. Proksch P. (2006). Chaetopyranin, a benzaldehyde derivative, and other related metabolites from *Chaetomium globosum*, an endophytic fungus derived from the marine red alga *Polysiphonia urceolata*. *J. Nat. Prod.* 69 1622–1625. 10.1021/np060248n 17125234

[B22] World Health Organization (2022). *Dementia.* Available online at: https://www.who.int/news-room/fact-sheets/detail/dementia (accessed September 21, 2022).

[B23] Yamazaki H. Koyama N. Ōmura S. Tomoda H. (2008). Structure-activity relationships of stemphones, potentiators of imipenem activity against methicillin-resistant *Staphylococcus aureus*. *J. Antibiot.* 61 426–441. 10.1038/ja.2008.59 18776655

[B24] Yatsu G. Kino Y. Sasaki H. Satoh J.-I. Kinoshita K. Koyama K. (2019). Meroterpenoids with BACE1 Inhibitory activity from the fruiting body of *Boletinus asiaticus*. *J. Nat. Prod.* 82 1797–1801. 10.1021/acs.jnatprod.8b01092 31244141

[B25] Yoshihira K. Takahashi C. Sekita S. Natori S. (1972). Tetrahydroauroglaucin from *Penicillium charlesii*. *Chem. Pharm. Bull.* 20 2727–2728. 10.1248/cpb.20.2727

[B26] Zhang P. Jia C. Deng Y. Chen S. Chen B. Yan S. (2019). Anti-inflammatory prenylbenzaldehyde derivatives isolated from *Eurotium cristatum*. *Phytochemistry* 158 120–125. 10.1016/j.phytochem.2018.11.017 30529862

[B27] Zhang Y. Jia A. Chen H. Wang M. Ding G. Sun L. (2017). Anthraquinones from the saline-alkali plant endophytic fungus *Eurotium rubrum*. *J. Antibiot.* 70 1138–1141. 10.1038/ja.2017.121 29018264

[B28] Zhao D. Cao F. Guo X.-J. Zhang Y.-R. Kang Z. Zhu H.-J. (2018). Antibacterial indole alkaloids and anthraquinones from a sewage-derived fungus *Eurotium* sp. *Chem. Nat. Compd.* 54 399–401. 10.1007/s10600-018-2361-8

[B29] Zhao Z.-Z. Zhao K. Chen H.-P. Bai X. Zhang L. Liu J.-K. (2018). Terpenoids from the mushroom-associated fungus *Montagnula donacina*. *Phytochemistry* 147 21–29. 10.1016/j.phytochem.2017.12.015 29287257

[B30] Zhong W. Chen Y. Mai Z. Wei X. Wang J. Zeng Q. (2020). Euroticins A and B, two pairs of highly constructed salicylaldehyde derivative enantiomers from a marine-derived fungus *Eurotium* sp. SCSIO F452. *J. Org. Chem.* 85 12754–12759. 10.1021/acs.joc.0c01407 32909756

[B31] Zhong W. Chen Y. Wei X. Wang J. Zhang W. Wang F. (2021). Salicylaldehyde derivatives from a marine-derived fungus *Eurotium* sp. SCSIO F452. *J. Antibiot.* 74 273–279. 10.1038/s41429-020-00395-x 33361799

[B32] Zhong W.-M. Wang J.-F. Shi X.-F. Wei X.-Y. Chen Y.-C. Zeng Q. (2018). Eurotiumins A–E, five new alkaloids from the marine-derived fungus *Eurotium* sp. SCSIO F452. *Mar. Drugs* 16:136. 10.3390/md16040136 29690501 PMC5923423

[B33] Zhong W.-M. Wei X.-Y. Chen Y.-C. Zeng Q. Wang J.-F. Shi X.-F. (2021). Structurally diverse polycyclic salicylaldehyde derivative enantiomers from a marine-derived fungus *Eurotium* sp. SCSIO F452. *Mar. Drugs* 19:543. 10.3390/md19100543 34677441 PMC8538301

