# Supplementary material for: Antibacterial and β-amyloid precursor protein-cleaving enzyme 1 inhibitory polyketides from the fungus Aspergillus chevalieri
Source: Front Microbiol. 2022 Nov 22;13:1051281. doi: 10.3389/fmicb.2022.1051281 (PMC9722750; doi:10.3389/fmicb.2022.1051281)
Supplement: Supplementary file 1 [file Data_Sheet_1.PDF]

Supplementary Material

Contents

1. Calculation details of **2**, **3**. 2
2. Standard orientation of the optimized conformers. 5
3. Supplementary figures of compounds **1–15**. 19

Supplementary Figure 1. ^{1}H NMR spectrum of (±)-**1** (600 MHz, $CDCl_3$). 19
Supplementary Figure 2. ^{13}C and DEPT NMR spectra of (±)-**1** (150 MHz, $CDCl_3$). 19
Supplementary Figure 3. HSQC spectrum of (±)-**1**. 20
Supplementary Figure 4. ^{1}H-^{1}H COSY spectrum of (±)-**1**. 20
Supplementary Figure 5. HMBC spectrum of (±)-**1.** 21
Supplementary Figure 6. ROESY spectrum of (±)-**1**. 21
Supplementary Figure 7. HRESIMS report of (±)-**1**. 22
Supplementary Figure 8. UV spectrum of compound (±)-**1**. 22
Supplementary Figure 9. ^{1}H NMR spectrum of **2–5** (600 MHz, $CDCl_3$). 23
Supplementary Figure 10. ^{13}C and DEPT NMR spectra of **2–5** (150 MHz, $CDCl_3$). 23
Supplementary Figure 11. ^{1}H NMR spectrum of **4**, **5** (600 MHz, $CDCl_3$) after first separation. 24
Supplementary Figure 12. ^{1}H NMR spectrum of **2** (600 MHz, $CDCl_3$) after first separation. 24
Supplementary Figure 13. ^{1}H NMR spectrum of **3** (600 MHz, $CDCl_3$) after first separation. 25
Supplementary Figure 14. ^{1}H NMR spectrum of **4** (600 MHz, $CDCl_3$) after second separation. 25
Supplementary Figure 15. ^{1}H NMR spectrum of **5** (600 MHz, $CDCl_3$) after second separation. 26
Supplementary Figure 16. ^{1}H NMR spectrum of **2** (600 MHz, $CDCl_3$). 27
Supplementary Figure 17. ^{13}C and DEPT NMR spectra of **2** (150 MHz, $CDCl_3$). 27
Supplementary Figure 18. HSQC spectrum of **2**. 28
Supplementary Figure 19. ^{1}H-^{1}H COSY spectrum of **2**. 28
Supplementary Figure 20. HMBC spectrum of **2**. 29
Supplementary Figure 21. ROESY spectrum of **2**. 29
Supplementary Figure 22. HRESIMS report of **2**. 30
Supplementary Figure 23. UV spectrum of compound **2**. 31
Supplementary Figure 24. CD spectra of compound **2**. 31
Supplementary Figure 25. ^{1}H NMR spectrum of **3** (600 MHz, $CDCl_3$). 32
Supplementary Figure 26. ^{13}C NMR spectra of **3** (150 MHz, $CDCl_3$). 32
Supplementary Figure 27. HRESIMS report of **3**. 33
Supplementary Figure 28. UV spectrum of compound **3**. 34
Supplementary Figure 29. CD spectra of compound **3**. 34
Supplementary Figure 30. ^{1}H NMR spectrum of **4** (600 MHz, $CDCl_3$). 35
Supplementary Figure 31. ^{13}C NMR spectra of **4** (150 MHz, $CDCl_3$). 35
Supplementary Figure 32. HRESIMS report of **4**. 36
Supplementary Figure 33. UV spectrum of compound **4**. 37
Supplementary Figure 34. CD spectra of compound **4**. 37
Supplementary Figure 35. ^{1}H NMR spectrum of **5** (600 MHz, $CDCl_3$). 38
Supplementary Figure 36. ^{13}C NMR spectra of **5** (150 MHz, $CDCl_3$). 38
Supplementary Figure 37. HRESIMS report of **5**. 39
Supplementary Figure 38. UV spectrum of compound **5**. 40

Supplementary Figure 39. CD spectra of compound **5**. 40
Supplementary Figure 40. ^{1}H NMR spectrum of **6** (600 MHz, $CDCl_3$). 41
Supplementary Figure 41. ^{13}C and DEPT NMR spectra of **6** (150 MHz, $CDCl_3$) 41
Supplementary Figure 42. ^{1}H NMR spectrum of **7** (600 MHz, $CDCl_3$). 42
Supplementary Figure 43. ^{13}C NMR spectra of **7** (150 MHz, $CDCl_3$). 42
Supplementary Figure 44. ^{1}H NMR spectrum of **8** (600 MHz, $CDCl_3$). 43
Supplementary Figure 45. ^{13}C NMR spectra of **8** (150 MHz, $CDCl_3$). 43
Supplementary Figure 46. ^{1}H NMR spectrum of **9** (600 MHz, $CDCl_3$). 44
Supplementary Figure 47. ^{13}C NMR spectra of **9** (150 MHz, $CDCl_3$). 44
Supplementary Figure 48. ^{1}H NMR spectrum of **10** (600 MHz, $CDCl_3$). 45
Supplementary Figure 49. ^{13}C NMR spectra of **10** (150 MHz, $CDCl_3$). 45
Supplementary Figure 50. ^{1}H NMR spectrum of **11** (600 MHz, acetone-*d*6). 46
Supplementary Figure 51. ^{13}C NMR spectra of **11** (150 MHz, acetone-*d*6). 46
Supplementary Figure 52. ^{1}H NMR spectrum of **12** (600 MHz, $CDCl_3$). 47
Supplementary Figure 53. ^{13}C NMR spectra of **12** (150 MHz, $CDCl_3$). 47
Supplementary Figure 54. ^{1}H NMR spectrum of **13** (600 MHz, $CDCl_3$). 48
Supplementary Figure 55. ^{13}C NMR spectra of **13** (150 MHz, $CDCl_3$). 48
Supplementary Figure 56. ^{1}H NMR spectrum of **14** (600 MHz, $CDCl_3$). 49
Supplementary Figure 57. ^{13}C NMR spectra of **14** (150 MHz, $CDCl_3$). 49
Supplementary Figure 58. ^{1}H NMR spectrum of **15** (600 MHz, $CDCl_3$). 50
Supplementary Figure 59. ^{13}C NMR spectrum of **15** (150 MHz, $CDCl_3$). 50
Supplementary Figure 60. Chiral separation of four racemates **2-5** by the column CHIRALPAK AS-H, (flow rate = 1 mL·min^{-1}, *n*-hexane/2-propanol = 97/3). 51
Supplementary Figure 61. Chiral separation of four racemates **4-5** by the column CHIRALPAK AD-H, (flow rate = 1 mL·min^{-1}, *n*-hexane/2-propanol = 94/6). 51
Supplementary Figure 62. IR spectrum of **2** 51
Supplementary Figure 63. Dose-response curve with IC_{50} value of compound **10** 52
4. Molecular identification result of the fungus 52

1. Calculation details of **2**, **3**.

Energy analysis of the calculated conformers of **2**, **3**.

**Species**	**Gibbs free energy**	***ΔE*** **(kcal/mol)**	P_E**%**
**(10*S*, 12*S*)-2a**	-1189.281965	1.68	7.6
**(10*S*, 12*S*)-2b**	-1189.284648	0.00	71.1
**(10*S*, 12*S*)-2c**	-1189.281792	1.79	8.1
**(10*S*, 12*S*)-2d**	-1189.282755	1.19	5.4
**(10*S*, 12*S*)-2e**	-1189.282755	1.19	5.4
**(10*S*, 12*S*)-2f**	-1189.281327	2.08	9.4
**(10*S*, 12*R*)-3a**	-1189.281205	0.00	31.9
**(10*S*, 12*R*)-3b**	-1189.280502	0.44	15.1
**(10*S*, 12*R*)-3c**	-1189.281011	0.12	25.9
**(10*S*, 12*R*)-3d**	-1189.279233	1.24	3.9
**(10*S*, 12*R*)-3e**	-1189.279808	0.88	7.3
**(10*S*, 12*R*)-3f**	-1189.280347	0.54	12.8
**(10*S*, 12*R*)-3g**	-1189.27899	1.39	3.0

Calculated ^{13}C NMR results for 2, 3.

No.	(10*S*, 12*S*)-2a	(10*S*, 12*S*)-2b	(10*S*, 12*S*)-2c	(10*S*, 12*S*)-2d	(10*S*, 12*S*)-2e	(10*S*, 12*S*)-2f	Calcd. Shielding Values	Exp.	Corr.	|DCorr-exp|	MAE
1C	87.855	92.1574	86.4282	87.8835	86.7197	84.7617	86.7	108.5	109.48	1.0	17.85
2C	37.8562	37.6343	37.6697	37.2811	37.6384	39.94	37.7	165.4	159.65	5.8	
3C	88.5159	101.901	89.6233	87.9394	89.0754	88.6197	89.9	99.4	106.20	6.8	
4C	34.3102	35.5686	34.7221	33.6994	34.3838	33.2891	34.7	165.5	162.72	2.8	
5C	75.9604	81.2238	75.9279	76.8742	76.9521	75.4554	76.2	110.9	120.28	9.4	
6C	44.9222	44.4463	44.8498	44.2063	44.1927	49.9672	44.9	145.8	152.28	6.5	
7C	10.702	10.8208	10.6998	10.3951	10.2086	12.1761	10.7	191.8	187.32	4.5	
8C	81.6687	80.9921	81.6575	81.2832	81.2819	80.8049	81.7	114.2	114.60	0.4	
9C	55.0021	55.7009	54.8558	54.8513	54.5906	51.798	54.9	145	142.05	2.9	
10C	147.2749	147.2467	147.2423	147.9055	147.9229	142.0904	147.4	47.6	47.33	0.3	
11C	35.1381	36.2601	35.1277	34.5833	34.6672	35.2891	35.2	162.3	162.26	0.0	
12C	81.9537	81.7442	82.055	81.8028	81.9612	82.5975	82.1	116.4	114.16	2.2	
13C	51.1401	51.8205	51.1633	51.2591	51.2151	50.2477	51.2	147.4	145.82	1.6	
14C	79.9026	79.7665	79.7206	79.4456	79.3164	80.3276	79.8	120.9	116.54	4.4	
15C	172.0324	171.7349	171.8411	172.0768	171.7685	172.6372	172.0	22.4	22.06	0.3	
19C	134.3934	142.0537	134.4429	136.1064	136.3591	134.1035	134.8	55.8	60.19	4.4	
21C	153.1198	153.054	153.4371	152.1616	152.0609	152.3825	153.5	40.2	41.03	0.8	
22C	181.3607	181.3176	181.2861	180.9961	181.2951	177.0898	181.4	16	12.44	3.6	
23C	148.7658	148.4632	149.2828	148.962	149.5323	153.1949	149.4	47	45.21	1.8	
24C	-12.434	-12.4893	-11.8002	-12.3553	-11.6434	-10.18	-11.8	207.9	210.42	2.5	
25C	164.6972	164.5504	164.9653	164.5715	164.6952	164.687	165.1	30.9	29.16	1.7	
27H	24.2144	24.5072	23.9426	24.2767	24.0365	24.4897	24.4897	6.39	6.50	0.1	1.77
28H	24.575	25.0347	24.923	24.6645	24.9612	24.7906	24.7906	6.31	6.24	0.1	
29H	27.4981	27.5072	27.2966	27.4012	27.1883	27.5744	27.5744	4.07	3.79	0.3	
30H	24.7087	24.7098	24.702	24.6119	24.6196	24.695	24.695	6.75	6.32	0.4	
31H	24.7406	24.8261	24.7333	24.8699	24.8626	24.7985	24.7985	6.71	6.23	0.5	
32H	29.5107	29.6014	29.5066	29.5302	29.528	29.5624	29.5624	2.38	2.04	0.3	
33H	29.0404	29.0821	29.0249	29.0728	29.0546	29.0598	29.0598	2.38	2.48	0.1	
34H	28.8192	29.0407	28.7902	28.8972	28.7974	29.0482	29.0482	2.38	2.49	0.1	
36H	27.4682	27.2552	26.8659	28.2218	28.2902	27.4287	27.4287	3.87	3.92	0.0	
37H	26.9284	27.7422	28.2033	27.201	27.1668	26.6587	26.6587	3.87	4.59	0.7	
38H	28.1468	27.8265	27.508	27.5341	27.5933	28.0546	28.0546	3.87	3.37	0.5	
40H	28.9767	28.9295	28.9358	29.0271	28.9493	28.9369	28.9369	2.5	2.59	0.1	
41H	31.409	31.4174	31.4289	31.4122	31.4073	30.1521	30.1521	0.62	1.52	0.9	
42H	30.9692	30.8582	31.0197	30.9764	31.0252	30.4554	30.4554	0.62	1.25	0.6	
43H	31.6697	31.6631	31.6284	31.6376	31.5974	30.6905	30.6905	0.62	1.05	0.4	
44H	29.3533	29.4009	29.3288	29.2886	29.2543	29.7636	29.7636	2.1	1.86	0.2	
45H	28.3773	28.4669	28.4713	28.3389	28.469	29.4216	29.4216	2.47	2.16	0.3	
46H	29.0138	29.0311	29.0217	29.0193	29.0254	29.6274	29.6274	2.12	1.98	0.1	
47H	29.1083	29.1338	29.1114	29.1171	29.1071	29.4061	29.4061	2.12	2.18	0.1	
48H	29.4819	29.5112	29.4641	29.511	29.499	29.9235	29.9235	2.12	1.72	0.4	

No.	**(10*S*, 12R)-3a**	**(10*S*, 12*R*)-3b**	**(10*S*, 12*R*)-3c**	**(10*S*, 12*R*)-3d**	**(10*S*, 12*R*)-3e**	**(10*S*, 12*R*)-3f**	**Calcd. Shielding Values**	**Exp.**	**Corr.**	**|δcorr-exp|**	**MAE**
1C	85.472	85.7315	86.7251	82.2681	85.93	86.7307	85.90	108.5	110.1	1.62	18.51
2C	39.295	39.1459	39.2981	38.1641	38.6599	39.2738	39.17	165.4	158.2	7.22	
3C	89.3551	89.1958	88.2805	88.6071	88.471	88.2518	88.88	99.4	107.0	7.65	
4C	34.0483	33.6861	33.8397	33.6004	33.6141	33.0844	33.77	165.5	163.7	1.77	
5C	74.6981	75.5337	73.6339	74.8508	76.2767	75.2461	74.88	110.9	121.4	10.54	
6C	49.6305	49.4937	50.22	49.953	49.0546	49.2698	49.66	145.8	147.4	1.59	
7C	11.8478	11.2646	12.0505	12.7882	11.8412	10.6773	11.65	191.8	186.5	5.32	
8C	81.8793	80.0747	80.8372	81.0934	81.8858	81.2206	81.01	114.2	115.1	0.94	
9C	51.7767	50.8799	52.2133	52.4915	52.6287	52.5972	51.76	145	145.2	0.23	
10C	145.9252	146.9714	146.1882	148.2604	142.0781	146.566	146.10	47.6	48.2	0.59	
11C	35.7338	35.9952	36.0164	36.0728	34.4903	35.1394	35.74	162.3	161.7	0.60	
12C	82.8065	82.9899	83.011	82.4809	82.1617	83.0138	82.87	116.4	113.2	3.17	
13C	49.5707	49.109	49.8414	51.4228	49.6706	47.2008	49.36	147.4	147.7	0.29	
14C	79.4487	79.3543	79.6616	80.4886	79.6075	79.6089	79.52	120.9	116.7	4.23	
15C	172.606	172.3604	172.8698	172.6777	171.9782	172.4483	172.53	22.4	21.0	1.39	
19C	133.9741	136.0523	134	133.875	133.6254	135.7594	134.72	55.8	59.9	4.10	
21C	152.8377	151.8032	152.5511	148.8333	152.4446	152.7456	152.31	40.2	41.8	1.61	
22C	181.7021	182.2088	181.1671	179.6768	183.9089	181.5834	181.85	16	11.4	4.57	
23C	148.9365	148.2013	149.5174	149.4806	146.2685	148.9366	148.64	47	45.6	1.42	
24C	-11.4276	-12.0582	-12.0567	-15.1053	-9.1166	-12.2495	-11.74	207.9	210.5	2.63	
25C	164.9246	165.0197	164.6567	163.9802	164.11	164.8532	164.79	30.9	29.0	1.93	
27H	25.0505	24.9075	25.2232	23.5649	24.6807	25.3006	25.00	6.31	6.17	0.14	1.19
28H	24.9194	24.82	24.5866	25.0471	24.8196	24.5135	24.77	6.37	6.37	0.00	
29H	27.3099	27.0798	27.0328	27.328	27.6187	26.8845	27.17	4.07	4.17	0.10	
30H	24.6905	24.7584	24.7529	24.6322	24.584	24.5955	24.71	6.71	6.43	0.28	
31H	24.1184	24.0565	24.1671	24.72	24.5393	24.2323	24.17	6.75	6.93	0.18	
32H	29.4458	29.6116	29.4647	29.4289	29.6087	29.4853	29.52	2.38	2.00	0.38	
33H	28.9086	28.9533	28.9473	28.9917	29.0549	28.9544	28.95	2.38	2.53	0.15	
34H	28.9952	28.9557	29.0141	29.1687	29.0017	28.9673	28.99	2.38	2.49	0.11	
36H	27.4059	27.6623	27.3725	27.4685	27.5044	27.539	27.50	3.87	3.87	0.00	
37H	26.7587	28.289	26.7959	26.7917	26.8293	28.1651	27.35	3.87	4.00	0.13	
38H	28.0831	27.3689	28.0393	28.1113	28.0913	27.443	27.81	3.87	3.58	0.29	
40H	28.9923	29.0206	29.1616	29.3694	28.9214	29.1486	29.06	2.5	2.43	0.07	
41H	31.396	31.3711	31.4148	31.1659	31.0481	31.4339	31.36	0.62	0.30	0.32	
42H	30.8956	30.8904	30.8971	30.7581	31.1081	30.914	30.91	0.62	0.72	0.10	
43H	31.4565	31.5563	31.4595	31.3333	31.7633	31.5959	31.52	0.62	0.16	0.46	
44H	28.6464	28.5058	28.6975	29.2926	28.8697	28.8651	28.67	2.1	2.79	0.69	
45H	29.2945	29.2784	29.133	29.0895	28.9277	29.1979	29.21	2.47	2.29	0.18	
46H	29.0421	29.0194	29.136	28.7614	29.4328	29.1443	29.09	2.12	2.40	0.28	
47H	29.4885	29.5018	29.5253	29.55	29.6586	29.5567	29.52	2.12	2.00	0.12	
48H	29.0858	29.0641	29.1073	29.0955	29.0866	29.1317	29.09	2.12	2.40	0.28	

2. Standard orientation of the optimized conformers.

Standard orientation of (10*S*, 12*S*)-2a

C	-1.41830000	2.11580000	-0.69720000
C	-2.79430000	2.23810000	-0.85540000
C	-3.63330000	1.15370000	-0.64060000
C	-3.09190000	-0.07300000	-0.24770000
C	-1.70480000	-0.22120000	-0.09710000
C	-0.85860000	0.88080000	-0.31770000
C	-1.08990000	-1.52260000	0.27200000
C	0.33870000	-1.74060000	-0.04900000
C	1.16400000	-0.63670000	-0.30780000
C	0.64820000	0.77870000	-0.11750000
C	0.85120000	-3.04130000	-0.13730000
C	2.16990000	-3.25550000	-0.52650000
C	2.99890000	-2.17010000	-0.82700000
C	2.48570000	-0.86870000	-0.72890000
C	4.40250000	-2.40130000	-1.30470000
O	0.10410000	-4.15650000	0.13340000
O	-1.71370000	-2.42950000	0.81440000
O	-3.94680000	-1.13660000	-0.12530000
C	-4.56970000	-1.12870000	1.15980000
O	-3.36180000	3.41950000	-1.22880000
C	1.05360000	1.44290000	1.24810000
C	0.72340000	0.57880000	2.47500000
C	2.53390000	1.86950000	1.31780000
C	2.89160000	2.98100000	0.34440000
C	4.34940000	3.35120000	0.26250000
O	2.04660000	3.56570000	-0.33270000
H	-0.76630000	2.97150000	-0.86410000
H	-4.70340000	1.25740000	-0.79420000
H	1.08070000	1.35730000	-0.94470000
H	2.53990000	-4.27520000	-0.60480000
H	3.12710000	-0.02840000	-0.98860000
H	4.81770000	-3.31700000	-0.87070000
H	4.41670000	-2.48820000	-2.39540000
H	5.05680000	-1.57550000	-1.00620000
H	-0.77260000	-3.85180000	0.46320000
H	-5.09750000	-2.07920000	1.28230000
H	-3.83590000	-1.04020000	1.96890000
H	-5.30580000	-0.32060000	1.22760000
H	-2.65840000	4.07500000	-1.37280000
H	0.46480000	2.36460000	1.36160000
H	0.93740000	1.12950000	3.39800000
H	-0.33610000	0.30940000	2.50400000
H	1.31860000	-0.33950000	2.49830000
H	2.75320000	2.26030000	2.31950000

H	3.20000000	1.01940000	1.14850000
H	4.69530000	3.69500000	1.24030000
H	4.92950000	2.48320000	-0.06020000
H	4.48520000	4.15640000	-0.46480000

Standard orientation of (10*S*, 12*S*)-2b

C	1.37710000	-2.12590000	-0.63100000
C	2.75340000	-2.26660000	-0.73630000
C	3.60560000	-1.19870000	-0.47610000
C	3.08560000	0.04540000	-0.09950000
C	1.68790000	0.19610000	0.01400000
C	0.82860000	-0.88890000	-0.25270000
C	1.06750000	1.49730000	0.39480000
C	-0.34240000	1.74160000	0.01490000
C	-1.17170000	0.65490000	-0.29390000
C	-0.68250000	-0.76820000	-0.10070000
C	-0.83220000	3.05090000	-0.07660000
C	-2.12940000	3.29040000	-0.51940000
C	-2.96060000	2.22170000	-0.86960000
C	-2.47090000	0.91160000	-0.76710000
C	-4.33930000	2.48020000	-1.40250000
O	-0.08110000	4.15050000	0.24330000
O	1.66660000	2.38750000	0.98990000
O	3.86320000	1.14980000	0.12330000
C	5.26960000	0.96980000	0.21430000
O	3.31670000	-3.45390000	-1.10070000
C	-1.14490000	-1.44220000	1.24160000
C	-0.83990000	-0.60110000	2.49070000
C	-2.63450000	-1.84090000	1.25650000
C	-2.98100000	-2.93310000	0.25760000
C	-4.44190000	-3.27530000	0.12530000
O	-2.12600000	-3.52460000	-0.40080000
H	0.71770000	-2.96800000	-0.83280000
H	4.67130000	-1.36530000	-0.59100000
H	-1.09480000	-1.33170000	-0.94850000
H	-2.48080000	4.31660000	-0.59860000
H	-3.11290000	0.08400000	-1.06360000
H	-4.75900000	3.39640000	-0.97410000
H	-4.30620000	2.58130000	-2.49160000
H	-5.01750000	1.66050000	-1.14290000
H	0.76970000	3.82520000	0.61990000
H	5.71070000	1.92740000	0.50740000
H	5.52910000	0.23480000	0.98350000
H	5.69220000	0.69350000	-0.75700000
H	2.60930000	-4.10220000	-1.25770000
H	-0.57730000	-2.37640000	1.36150000

H	-1.09650000	-1.15910000	3.39830000
H	0.22320000	-0.35410000	2.55970000
H	-1.41710000	0.32880000	2.50610000
H	-2.89470000	-2.23930000	2.24530000
H	-3.27800000	-0.97580000	1.07590000
H	-4.82480000	-3.62480000	1.08720000
H	-4.99580000	-2.39270000	-0.20400000
H	-4.56930000	-4.06870000	-0.61630000

Standard orientation of (10*S*, 12*S*)-2c

C	1.48780000	-2.12630000	-0.72100000
C	2.86510000	-2.22580000	-0.88100000
C	3.68880000	-1.13200000	-0.65160000
C	3.12250000	0.07930000	-0.24280000
C	1.73170000	0.20530000	-0.09770000
C	0.90360000	-0.90760000	-0.33190000
C	1.09440000	1.49400000	0.27880000
C	-0.33530000	1.69200000	-0.04810000
C	-1.14000000	0.57730000	-0.32160000
C	-0.60540000	-0.83200000	-0.13350000
C	-0.86720000	2.98520000	-0.13490000
C	-2.18410000	3.18130000	-0.53930000
C	-2.99130000	2.08500000	-0.85910000
C	-2.45830000	0.79160000	-0.76240000
C	-4.39120000	2.29620000	-1.35620000
O	-0.14030000	4.11030000	0.15020000
O	1.70260000	2.40770000	0.82750000
O	3.96690000	1.14900000	-0.09850000
C	4.52550000	1.16750000	1.21630000
O	3.37220000	-3.42820000	-1.27330000
C	-1.00770000	-1.50390000	1.22990000
C	-0.54320000	-0.71180000	2.46370000
C	-2.51530000	-1.79640000	1.37710000
C	-3.05250000	-2.80600000	0.37760000
C	-4.54280000	-3.02570000	0.38950000
O	-2.32440000	-3.42840000	-0.39340000
H	0.86660000	-3.00220000	-0.90510000
H	4.76230000	-1.19610000	-0.79590000
H	-1.02800000	-1.41670000	-0.96120000
H	-2.56920000	4.19550000	-0.61640000
H	-3.08100000	-0.05800000	-1.03800000
H	-4.82720000	3.20310000	-0.92400000
H	-4.39090000	2.38830000	-2.44660000
H	-5.03640000	1.45860000	-1.07110000
H	0.73810000	3.81680000	0.48520000
H	3.75790000	1.07060000	1.99230000

H	5.27240000	0.37400000	1.32600000
H	5.02880000	2.12920000	1.35330000
H	4.33250000	-3.34200000	-1.39360000
H	-0.49680000	-2.47630000	1.28180000
H	-0.78030000	-1.26100000	3.38200000
H	0.53900000	-0.55520000	2.46080000
H	-1.03680000	0.26290000	2.52960000
H	-2.70480000	-2.22430000	2.36980000
H	-3.10430000	-0.87770000	1.30590000
H	-4.85150000	-3.39010000	1.37230000
H	-5.05250000	-2.08740000	0.15690000
H	-4.81210000	-3.76990000	-0.36500000

Standard orientation of (10*S*, 12*S*)-2d

C	1.31390000	-2.22200000	-0.57090000
C	2.68380000	-2.45710000	-0.59540000
C	3.58130000	-1.45340000	-0.26210000
C	3.10600000	-0.18420000	0.07820000
C	1.72590000	0.07130000	0.12340000
C	0.82030000	-0.95580000	-0.19990000
C	1.18420000	1.41240000	0.46920000
C	-0.20830000	1.73570000	0.08030000
C	-1.08880000	0.70200000	-0.26870000
C	-0.68610000	-0.75130000	-0.10100000
C	-0.62680000	3.07140000	0.00490000
C	-1.90110000	3.38940000	-0.45520000
C	-2.78080000	2.37490000	-0.84370000
C	-2.36270000	1.03910000	-0.76140000
C	-4.13320000	2.71760000	-1.39600000
O	0.17520000	4.12510000	0.35510000
O	1.85250000	2.28440000	1.01560000
O	4.04110000	0.75740000	0.41790000
C	4.44620000	1.49730000	-0.73390000
O	3.18710000	-3.67650000	-0.93680000
C	-1.23190000	-1.43060000	1.20800000
C	-0.93220000	-0.63150000	2.48590000
C	-2.73840000	-1.75680000	1.16190000
C	-3.10160000	-2.80780000	0.12530000
C	-4.57100000	-3.08790000	-0.05230000
O	-2.25400000	-3.41420000	-0.52900000
H	0.61440000	-3.01820000	-0.81980000
H	4.64860000	-1.65450000	-0.26730000
H	-1.09750000	-1.26870000	-0.97800000
H	-2.19560000	4.43450000	-0.51920000
H	-3.04150000	0.25410000	-1.09010000
H	-4.51180000	3.64610000	-0.95590000

H	-4.07380000	2.83880000	-2.48200000
H	-4.85890000	1.93030000	-1.16630000
H	1.00460000	3.75100000	0.73350000
H	4.98750000	0.85600000	-1.43800000
H	3.59390000	1.97110000	-1.23300000
H	5.12680000	2.28720000	-0.40250000
H	2.44910000	-4.28090000	-1.12380000
H	-0.71610000	-2.39430000	1.32760000
H	-1.24490000	-1.19670000	3.37130000
H	0.13800000	-0.43490000	2.59590000
H	-1.46680000	0.32340000	2.50360000
H	-3.05110000	-2.16520000	2.13130000
H	-3.33410000	-0.85810000	0.98190000
H	-4.99180000	-3.44710000	0.88990000
H	-5.08110000	-2.17540000	-0.37080000
H	-4.71020000	-3.85570000	-0.81820000

Standard orientation of (10*S*, 12*S*)-2e

C	1.41910000	-2.21970000	-0.59320000
C	2.79530000	-2.41260000	-0.61470000
C	3.66220000	-1.38220000	-0.27890000
C	3.14120000	-0.13090000	0.06430000
C	1.75260000	0.07730000	0.11490000
C	0.88040000	-0.97540000	-0.21770000
C	1.16640000	1.39150000	0.48880000
C	-0.23150000	1.67910000	0.09460000
C	-1.07550000	0.62410000	-0.27760000
C	-0.63250000	-0.81950000	-0.12000000
C	-0.68780000	3.00270000	0.03030000
C	-1.96390000	3.28890000	-0.44540000
C	-2.80600000	2.25380000	-0.86230000
C	-2.34950000	0.93010000	-0.78990000
C	-4.15840000	2.56250000	-1.43430000
O	0.07860000	4.07420000	0.40530000
O	1.80330000	2.26930000	1.06280000
O	4.05110000	0.83720000	0.40000000
C	4.36280000	1.64780000	-0.73380000
O	3.25750000	-3.64530000	-0.96630000
C	-1.16630000	-1.52310000	1.18170000
C	-0.74220000	-0.81200000	2.47810000
C	-2.69450000	-1.73130000	1.21820000
C	-3.22230000	-2.66270000	0.14080000
C	-4.71940000	-2.81480000	0.06840000
O	-2.48370000	-3.27450000	-0.62870000
H	0.76260000	-3.04990000	-0.85150000
H	4.73770000	-1.52730000	-0.28000000

H	-1.02640000	-1.34340000	-1.00080000
H	-2.28870000	4.32540000	-0.50120000
H	-2.99760000	0.12850000	-1.14070000
H	-4.57190000	3.47440000	-0.99090000
H	-4.08420000	2.69760000	-2.51760000
H	-4.86410000	1.75150000	-1.22540000
H	0.91140000	3.71870000	0.79340000
H	4.88880000	1.06180000	-1.49540000
H	3.46870000	2.11000000	-1.16570000
H	5.03040000	2.44770000	-0.40010000
H	4.22730000	-3.64760000	-0.90570000
H	-0.71730000	-2.52600000	1.22300000
H	-1.07110000	-1.38420000	3.35310000
H	0.34490000	-0.71960000	2.55250000
H	-1.18340000	0.18660000	2.55610000
H	-2.97230000	-2.18980000	2.17590000
H	-3.22510000	-0.77710000	1.15380000
H	-5.09320000	-3.21430000	1.01420000
H	-5.17580000	-1.84370000	-0.13870000
H	-4.98140000	-3.50680000	-0.73670000

Standard orientation of (10*S*, 12*S*)-2f

C	-2.17690000	0.75960000	-1.71150000
C	-3.47150000	0.30860000	-1.47770000
C	-3.72190000	-0.67740000	-0.53520000
C	-2.65460000	-1.21880000	0.18600000
C	-1.33770000	-0.78990000	-0.05270000
C	-1.09120000	0.22720000	-0.99420000
C	-0.18000000	-1.42040000	0.63160000
C	1.15750000	-1.29530000	0.01310000
C	1.38660000	-0.28330000	-0.92810000
C	0.31480000	0.74840000	-1.24950000
C	2.18500000	-2.18200000	0.35940000
C	3.43330000	-2.08830000	-0.24880000
C	3.68030000	-1.08980000	-1.19680000
C	2.65260000	-0.19830000	-1.53610000
C	5.01550000	-1.00500000	-1.87660000
O	2.03010000	-3.17370000	1.29100000
O	-0.28440000	-2.04560000	1.68220000
O	-2.93820000	-2.25430000	1.03750000
C	-3.31290000	-1.75560000	2.32270000
O	-4.47550000	0.86400000	-2.21270000
C	0.63940000	2.11460000	-0.54970000
C	-0.12930000	3.28150000	-1.19110000
C	0.41290000	2.09510000	0.97700000
C	1.07940000	3.24890000	1.71230000
C	0.71960000	3.40340000	3.16740000
O	1.89020000	4.00270000	1.17670000
H	-2.02170000	1.52460000	-2.46970000
H	-4.72590000	-1.05200000	-0.36420000
H	0.38440000	0.90080000	-2.33760000
H	4.21160000	-2.79690000	0.02530000
H	2.84620000	0.58020000	-2.27360000
H	5.81310000	-1.36650000	-1.21910000

H	5.00890000	-1.60820000	-2.78950000
H	5.25510000	0.03110000	-2.13760000
H	1.14400000	-3.05670000	1.70350000
H	-3.34520000	-2.60220000	3.01470000
H	-2.59070000	-1.02710000	2.70810000
H	-4.31230000	-1.30930000	2.28390000
H	-5.31230000	0.42140000	-1.99110000
H	1.70220000	2.32760000	-0.73170000
H	0.27120000	4.24510000	-0.86050000
H	-0.04200000	3.25510000	-2.28230000
H	-1.19060000	3.26680000	-0.92580000
H	-0.65930000	2.12330000	1.20190000
H	0.83260000	1.18670000	1.41950000
H	-0.35600000	3.57080000	3.26310000
H	1.01200000	2.50350000	3.71400000
H	1.24840000	4.26260000	3.58920000

Standard orientation of (10*S*, 12*R*)-3a

C	-2.25240000	0.58400000	-0.80930000
C	-3.33530000	-0.26760000	-0.99560000
C	-3.22390000	-1.62600000	-0.73890000
C	-2.00390000	-2.13890000	-0.28920000
C	-0.89230000	-1.29660000	-0.11660000
C	-1.02100000	0.08640000	-0.34900000
C	0.44390000	-1.83480000	0.25240000
C	1.63980000	-1.01640000	-0.04650000
C	1.49070000	0.35620000	-0.28000000
C	0.14130000	1.03890000	-0.11570000
C	2.90980000	-1.60440000	-0.10340000
C	4.02770000	-0.84180000	-0.43060000
C	3.89270000	0.52350000	-0.70520000
C	2.62670000	1.11760000	-0.61080000
C	5.09940000	1.35520000	-1.02620000
O	3.12680000	-2.93240000	0.15210000
O	0.60120000	-2.93460000	0.77360000
O	-1.92090000	-3.49840000	-0.13900000
C	-2.31380000	-3.87800000	1.18120000
O	-4.49960000	0.28120000	-1.44320000
C	0.08790000	1.80690000	1.25500000
C	-0.04000000	0.87840000	2.47340000
C	-1.01540000	2.88200000	1.33310000
C	-0.82990000	4.02390000	0.34800000
C	-1.97180000	4.99970000	0.23590000
O	0.19900000	4.16920000	-0.31040000
H	-2.37380000	1.63890000	-1.04340000
H	-4.05750000	-2.30150000	-0.90180000
H	0.09310000	1.76490000	-0.93840000
H	5.00330000	-1.32070000	-0.46990000
H	2.52230000	2.18910000	-0.78760000
H	5.86060000	0.75790000	-1.53900000

H	4.83580000	2.18450000	-1.69090000
H	5.52920000	1.76160000	-0.10560000
H	2.27240000	-3.31650000	0.45490000
H	-2.05230000	-4.93130000	1.31930000
H	-1.79680000	-3.29410000	1.95080000
H	-3.39800000	-3.77790000	1.30020000
H	-5.14160000	-0.43160000	-1.60240000
H	1.04290000	2.33820000	1.37690000
H	0.01290000	1.45790000	3.40210000
H	0.77170000	0.14650000	2.51010000
H	-0.99410000	0.34200000	2.47740000
H	-2.00580000	2.44210000	1.18780000
H	-1.00860000	3.33840000	2.33110000
H	-2.14480000	5.47360000	1.20510000
H	-1.72620000	5.77220000	-0.49800000
H	-2.87130000	4.47390000	-0.09380000

Standard orientation of (10*S*, 12*R*)-3b

C	-2.36770000	-0.10960000	-0.67100000
C	-3.19340000	-1.22710000	-0.70500000
C	-2.71550000	-2.47440000	-0.33330000
C	-1.37660000	-2.60850000	0.04510000
C	-0.53230000	-1.48590000	0.10790000
C	-1.03640000	-0.21540000	-0.23130000
C	0.90610000	-1.60920000	0.46700000
C	1.82780000	-0.51760000	0.07960000
C	1.30540000	0.73540000	-0.26180000
C	-0.17840000	1.03480000	-0.11530000
C	3.21100000	-0.73640000	0.03340000
C	4.07380000	0.27250000	-0.38660000
C	3.56610000	1.51900000	-0.76780000
C	2.18580000	1.74800000	-0.68640000
C	4.49460000	2.61960000	-1.18880000
O	3.78820000	-1.92730000	0.38720000
O	1.37340000	-2.59790000	1.02350000
O	-0.96630000	-3.86680000	0.40010000
C	-0.40760000	-4.54970000	-0.72330000
O	-4.48280000	-1.04680000	-1.10720000
C	-0.42900000	1.87810000	1.18840000
C	-0.28470000	1.06180000	2.48310000
C	-1.78380000	2.61480000	1.21680000
C	-1.93300000	3.66860000	0.13250000
C	-3.29800000	4.28930000	-0.01010000
O	-0.99540000	4.02130000	-0.58160000
H	-2.77530000	0.84820000	-0.98550000
H	-3.35550000	-3.35080000	-0.34200000
H	-0.43000000	1.64340000	-0.99410000
H	5.14350000	0.07850000	-0.41390000
H	1.78870000	2.73030000	-0.94660000
H	5.38920000	2.21290000	-1.67200000
H	4.00930000	3.28410000	-1.91140000
H	4.79910000	3.20620000	-0.31660000
H	3.07700000	-2.49820000	0.75890000
H	-0.00430000	-5.50290000	-0.36910000
H	-1.18160000	-4.76380000	-1.46830000

H	0.41020000	-3.98440000	-1.18290000
H	-4.94200000	-1.90370000	-1.08280000
H	0.34410000	2.65880000	1.23390000
H	-0.38390000	1.71350000	3.35850000
H	0.69710000	0.58530000	2.55270000
H	-1.05460000	0.28790000	2.56340000
H	-2.61760000	1.91100000	1.14840000
H	-1.88830000	3.14570000	2.17160000
H	-3.57300000	4.78720000	0.92290000
H	-3.28490000	5.02830000	-0.81600000
H	-4.02850000	3.51420000	-0.25460000

Standard orientation of (10*S*, 12*R*)-3c

C	-2.27490000	0.36990000	-0.86840000
C	-3.26840000	-0.58680000	-1.05140000
C	-3.03470000	-1.92120000	-0.75370000
C	-1.78240000	-2.31300000	-0.27690000
C	-0.75950000	-1.36690000	-0.10740000
C	-1.01260000	-0.00560000	-0.37160000
C	0.61560000	-1.77050000	0.28920000
C	1.73410000	-0.85730000	-0.03440000
C	1.46220000	0.49140000	-0.29680000
C	0.05650000	1.05120000	-0.13680000
C	3.05060000	-1.33120000	-0.08200000
C	4.09500000	-0.47910000	-0.43050000
C	3.83800000	0.86270000	-0.73420000
C	2.52440000	1.34430000	-0.64760000
C	4.96580000	1.79070000	-1.07790000
O	3.38490000	-2.62800000	0.20400000
O	0.86760000	-2.83060000	0.85300000
O	-1.56670000	-3.65410000	-0.10060000
C	-1.99970000	-4.06020000	1.19830000
O	-4.49730000	-0.25030000	-1.53500000
C	-0.06830000	1.81630000	1.23130000
C	-0.05540000	0.88650000	2.45650000
C	-1.29610000	2.74460000	1.33590000
C	-1.26700000	3.92580000	0.38060000
C	-2.43740000	4.87140000	0.45710000
O	-0.35030000	4.11360000	-0.41700000
H	-2.46940000	1.40890000	-1.11970000
H	-3.81210000	-2.66060000	-0.92350000
H	-0.05530000	1.76630000	-0.96210000
H	5.10940000	-0.86970000	-0.46280000
H	2.32480000	2.39790000	-0.84630000
H	5.77480000	1.25240000	-1.58280000
H	4.62790000	2.58020000	-1.75740000
H	5.36140000	2.25170000	-0.16770000
H	2.56950000	-3.07840000	0.52320000
H	-1.65770000	-5.08670000	1.36020000

H	-1.57330000	-3.43060000	1.98740000
H	-3.09320000	-4.05260000	1.26000000
H	-4.48130000	0.67180000	-1.83910000
H	0.81570000	2.46290000	1.33010000
H	-0.06650000	1.47320000	3.38210000
H	0.84660000	0.26920000	2.48690000
H	-0.93010000	0.22890000	2.47620000
H	-2.22760000	2.19160000	1.18690000
H	-1.33360000	3.17490000	2.34500000
H	-2.49390000	5.30460000	1.45870000
H	-2.30840000	5.67780000	-0.27010000
H	-3.35900000	4.33150000	0.22610000

Standard orientation of (10*S*, 12*R*)-3d

C	-1.78380000	1.56360000	-0.80700000
C	-3.13900000	1.29800000	-0.97240000
C	-3.65710000	0.04120000	-0.69580000
C	-2.80070000	-0.96710000	-0.24580000
C	-1.42540000	-0.72320000	-0.09480000
C	-0.90920000	0.56220000	-0.35050000
C	-0.47950000	-1.80720000	0.27960000
C	0.95200000	-1.63560000	-0.05190000
C	1.44550000	-0.35180000	-0.31670000
C	0.56350000	0.87710000	-0.14260000
C	1.80670000	-2.74330000	-0.11130000
C	3.14110000	-2.58710000	-0.47560000
C	3.64190000	-1.31850000	-0.78740000
C	2.79480000	-0.20580000	-0.68890000
C	5.08610000	-1.14520000	-1.15580000
O	1.39480000	-4.01740000	0.17640000
O	-0.83260000	-2.84430000	0.83230000
O	-3.34330000	-2.21350000	-0.07270000
C	-3.86540000	-2.34870000	1.25020000
O	-3.93280000	2.31120000	-1.42000000
C	0.89340000	1.58310000	1.22360000
C	0.34620000	0.83750000	2.45080000
C	0.44270000	3.05330000	1.29440000
C	1.21670000	3.93150000	0.33280000
C	2.49480000	4.54790000	0.83700000
O	0.83840000	4.09670000	-0.82630000
H	-1.41240000	2.55730000	-1.05360000
H	-4.70930000	-0.17950000	-0.84300000
H	0.85000000	1.54370000	-0.96710000
H	3.78470000	-3.46280000	-0.51770000
H	3.19130000	0.78650000	-0.90140000
H	5.46810000	-2.03430000	-1.66840000
H	5.21580000	-0.29750000	-1.83670000
H	5.68340000	-0.97130000	-0.25550000
H	0.46560000	-3.96090000	0.49740000
H	-4.11340000	-3.40260000	1.40690000
H	-3.13740000	-2.05230000	2.01370000
H	-4.78420000	-1.76280000	1.36050000
H	-4.83110000	1.96960000	-1.56340000
H	1.98710000	1.59130000	1.33480000
H	0.68050000	1.32490000	3.37370000
H	0.70620000	-0.19420000	2.49010000

H	-0.74820000	0.82680000	2.46550000
H	-0.62740000	3.17250000	1.10340000
H	0.60630000	3.45450000	2.30260000
H	3.20870000	3.76160000	1.09240000
H	2.92880000	5.18160000	0.05850000
H	2.28390000	5.16620000	1.71300000

Standard orientation of (10*S*, 12*R*)-3e

C	-2.17700000	-0.34930000	-1.32220000
C	-2.98170000	-1.48300000	-1.30570000
C	-2.55910000	-2.64570000	-0.67950000
C	-1.30720000	-2.67080000	-0.05870000
C	-0.47080000	-1.54140000	-0.08440000
C	-0.91640000	-0.35540000	-0.69900000
C	0.90530000	-1.57910000	0.47660000
C	1.88220000	-0.56710000	0.01800000
C	1.42190000	0.60530000	-0.59520000
C	-0.06580000	0.90530000	-0.69550000
C	3.25670000	-0.77920000	0.18750000
C	4.17900000	0.15160000	-0.28200000
C	3.74030000	1.31220000	-0.92770000
C	2.36370000	1.53970000	-1.06490000
C	4.73110000	2.33530000	-1.40010000
O	3.76810000	-1.88740000	0.80900000
O	1.27370000	-2.42490000	1.28570000
O	-0.90050000	-3.86930000	0.46670000
C	-1.33230000	-3.99030000	1.82340000
O	-4.19040000	-1.40740000	-1.93040000
C	-0.48890000	1.96780000	0.37820000
C	-0.47770000	1.42470000	1.81660000
C	-1.86190000	2.59710000	0.06200000
C	-2.09540000	3.93280000	0.75260000
C	-3.52260000	4.41180000	0.81130000
O	-1.17760000	4.61750000	1.20120000
H	-2.54000000	0.53630000	-1.83840000
H	-3.17100000	-3.54190000	-0.68000000
H	-0.19820000	1.35940000	-1.68960000
H	5.24070000	-0.03490000	-0.13780000
H	2.02200000	2.46280000	-1.53210000
H	5.67430000	1.86100000	-1.69100000
H	4.35200000	2.86870000	-2.27820000
H	4.92950000	3.05930000	-0.60390000
H	3.00830000	-2.39870000	1.17060000
H	-0.82840000	-4.85770000	2.25980000
H	-1.07310000	-3.10870000	2.42040000
H	-2.41240000	-4.16690000	1.86560000
H	-4.61600000	-2.28120000	-1.89810000
H	0.25540000	2.77510000	0.34480000

H	-0.71260000	2.21740000	2.53470000
H	0.50660000	1.03280000	2.08890000
H	-1.21650000	0.62920000	1.95630000
H	-1.95290000	2.79340000	-1.01250000
H	-2.66820000	1.91850000	0.36150000
H	-4.12970000	3.68850000	1.36120000
H	-3.56790000	5.37410000	1.32880000
H	-3.90870000	4.53560000	-0.20340000

Standard orientation of (10*S**, 12*R**)-3f

C	2.31570000	0.37000000	-0.73780000
C	3.00410000	1.57830000	-0.77550000
C	2.40080000	2.75230000	-0.35120000
C	1.06980000	2.73150000	0.07020000
C	0.35790000	1.52180000	0.12120000
C	0.99410000	0.32150000	-0.25480000
C	-1.08320000	1.47950000	0.49070000
C	-1.88190000	0.30190000	0.08020000
C	-1.22650000	-0.88110000	-0.28280000
C	0.28100000	-1.01700000	-0.13720000
C	-3.28050000	0.36950000	0.03700000
C	-4.02840000	-0.72000000	-0.40130000
C	-3.38830000	-1.89730000	-0.80330000
C	-1.99120000	-1.97650000	-0.72460000
C	-4.19280000	-3.08460000	-1.24400000
O	-3.98340000	1.48380000	0.41190000
O	-1.65480000	2.39740000	1.07030000
O	0.52740000	3.92190000	0.47610000
C	-0.06190000	4.60750000	-0.62920000
O	4.28900000	1.65060000	-1.22330000
C	0.62460000	-1.82920000	1.16560000
C	0.32860000	-1.05800000	2.46320000
C	2.07290000	-2.35640000	1.23240000
C	2.40600000	-3.40110000	0.18080000
C	3.79930000	-3.97250000	0.22990000
O	1.59940000	-3.76790000	-0.67160000
H	2.80020000	-0.54140000	-1.07740000
H	2.95580000	3.68600000	-0.36450000
H	0.59650000	-1.59310000	-1.01650000
H	-5.11290000	-0.64300000	-0.42620000
H	-1.49020000	-2.90500000	-1.00110000
H	-5.12550000	-2.76870000	-1.72300000
H	-3.63830000	-3.68150000	-1.97570000
H	-4.43310000	-3.71440000	-0.38200000
H	-3.33820000	2.12270000	0.79380000
H	-0.57040000	5.49480000	-0.24060000
H	0.70700000	4.93870000	-1.33560000
H	-0.80500000	3.98790000	-1.14320000
H	4.55880000	0.78480000	-1.57040000
H	-0.02590000	-2.71580000	1.18280000
H	0.50980000	-1.69480000	3.33660000
H	-0.71650000	-0.74120000	2.51730000
H	0.96730000	-0.17500000	2.56490000
H	2.79810000	-1.54090000	1.16330000
H	2.22940000	-2.84580000	2.20240000
H	3.96090000	-4.46260000	1.19300000
H	3.92380000	-4.71040000	-0.56740000

H	4.52810000	-3.17100000	0.08650000

Standard orientation of (10*S*, 12*R*)-3g

C	-1.58840000	1.82110000	-0.66940000
C	-2.97830000	1.78320000	-0.68360000
C	-3.66210000	0.64150000	-0.29370000
C	-2.93880000	-0.49290000	0.08370000
C	-1.53390000	-0.46650000	0.12600000
C	-0.84620000	0.70920000	-0.23380000
C	-0.74180000	-1.67240000	0.48740000
C	0.67840000	-1.73410000	0.07510000
C	1.34040000	-0.55620000	-0.29200000
C	0.66820000	0.80080000	-0.13900000
C	1.35260000	-2.96140000	0.02820000
C	2.66860000	-3.02930000	-0.42080000
C	3.33210000	-1.86900000	-0.83300000
C	2.66910000	-0.63690000	-0.74980000
C	4.75890000	-1.93620000	-1.29250000
O	0.77430000	-4.14340000	0.40830000
O	-1.22290000	-2.64120000	1.06670000
O	-3.67150000	-1.58880000	0.45790000
C	-3.88700000	-2.45370000	-0.65830000
O	-3.63860000	2.90550000	-1.08510000
C	1.19870000	1.51310000	1.16000000
C	0.63000000	0.92460000	2.46070000
C	0.98940000	3.03810000	1.17220000
C	1.82180000	3.73330000	0.11450000
C	3.20340000	4.18330000	0.50970000
O	1.40210000	3.88250000	-1.03250000
H	-1.08760000	2.73180000	-0.99520000
H	-4.74670000	0.60730000	-0.28740000
H	0.99240000	1.37260000	-1.01870000
H	3.16960000	-3.99420000	-0.44940000
H	3.19500000	0.27190000	-1.04100000
H	4.96980000	-2.89700000	-1.77380000
H	4.96910000	-1.15210000	-2.02750000
H	5.43280000	-1.81200000	-0.43930000
H	-0.10740000	-3.92900000	0.79160000
H	-4.38860000	-3.35430000	-0.29220000
H	-4.54110000	-1.97650000	-1.39620000
H	-2.94600000	-2.75690000	-1.12990000
H	-4.59550000	2.74270000	-1.04060000
H	2.28560000	1.35330000	1.20340000
H	1.10170000	1.39520000	3.33080000
H	0.82470000	-0.14870000	2.53740000
H	-0.44860000	1.09100000	2.54460000

H	-0.05930000	3.31900000	1.04250000
H	1.28520000	3.45530000	2.14320000
H	3.80840000	3.31720000	0.78780000
H	3.67920000	4.69200000	-0.33330000
H	3.13660000	4.88240000	1.34690000

3. Supplementary figures of compounds 1–15.

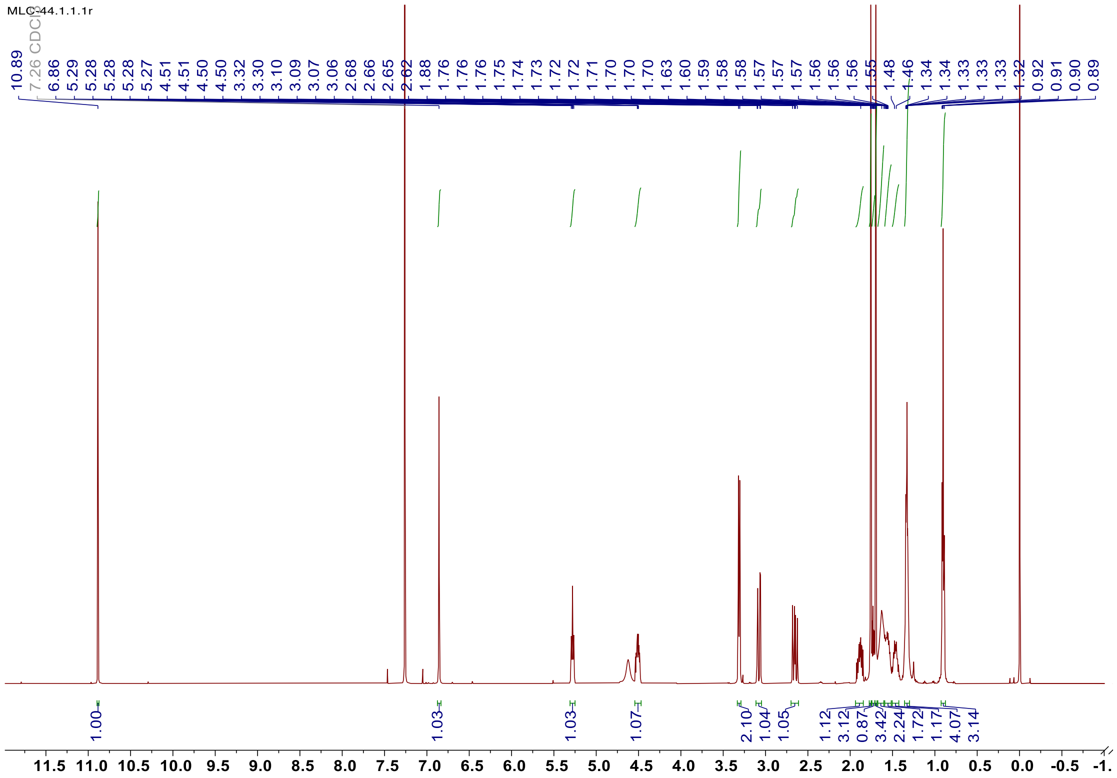

Supplementary Figure 1. ^{1}H NMR spectrum of (±)-**1** (600 MHz, $CDCl_3$).

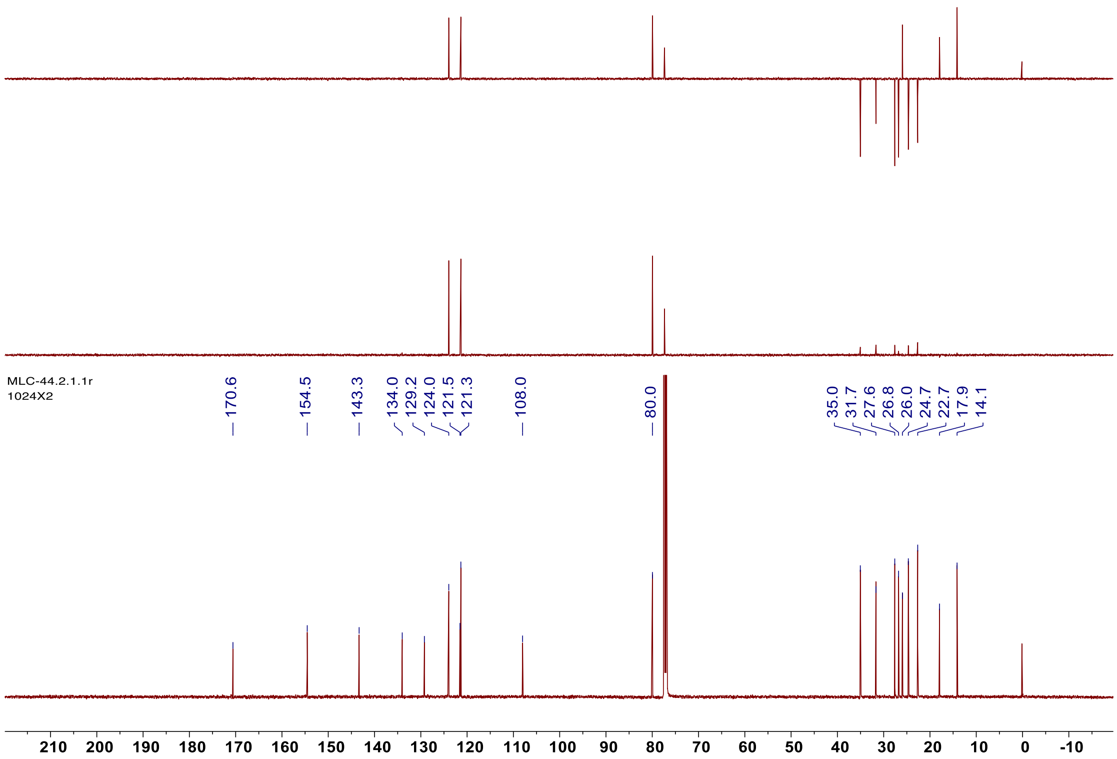

Supplementary Figure 2. ^{13}C and DEPT NMR spectra of (±)-**1** (150 MHz, $CDCl_3$).

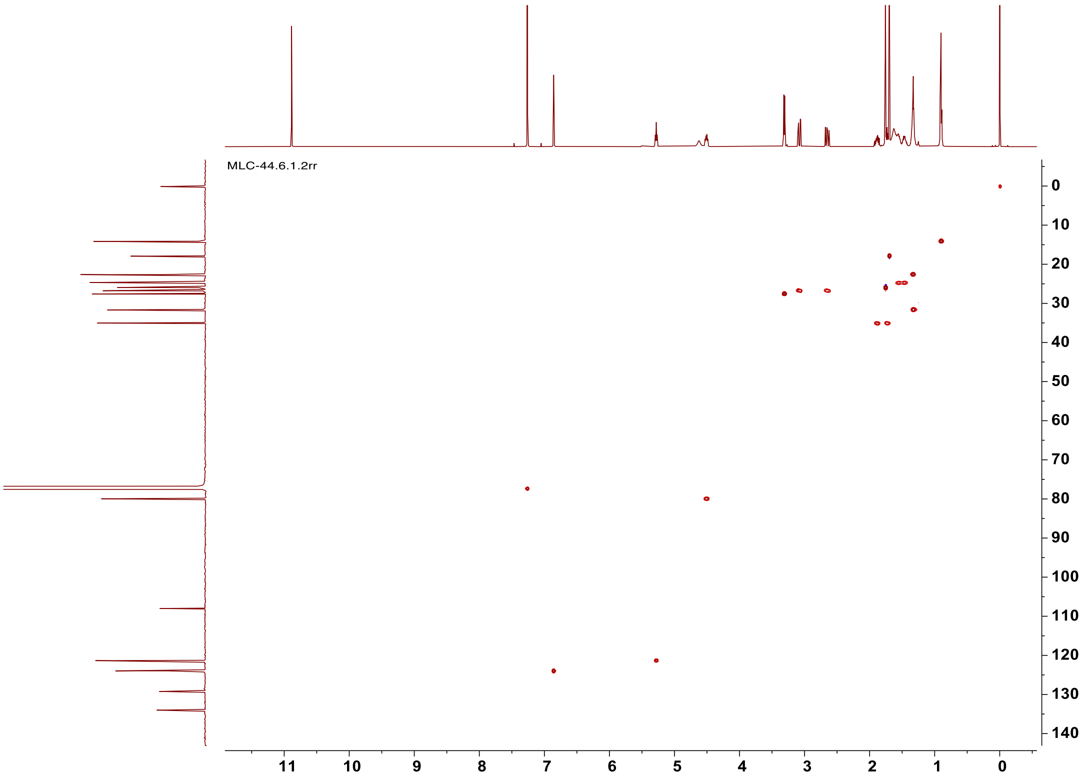

Supplementary Figure 3. HSQC spectrum of (±)-**1**.

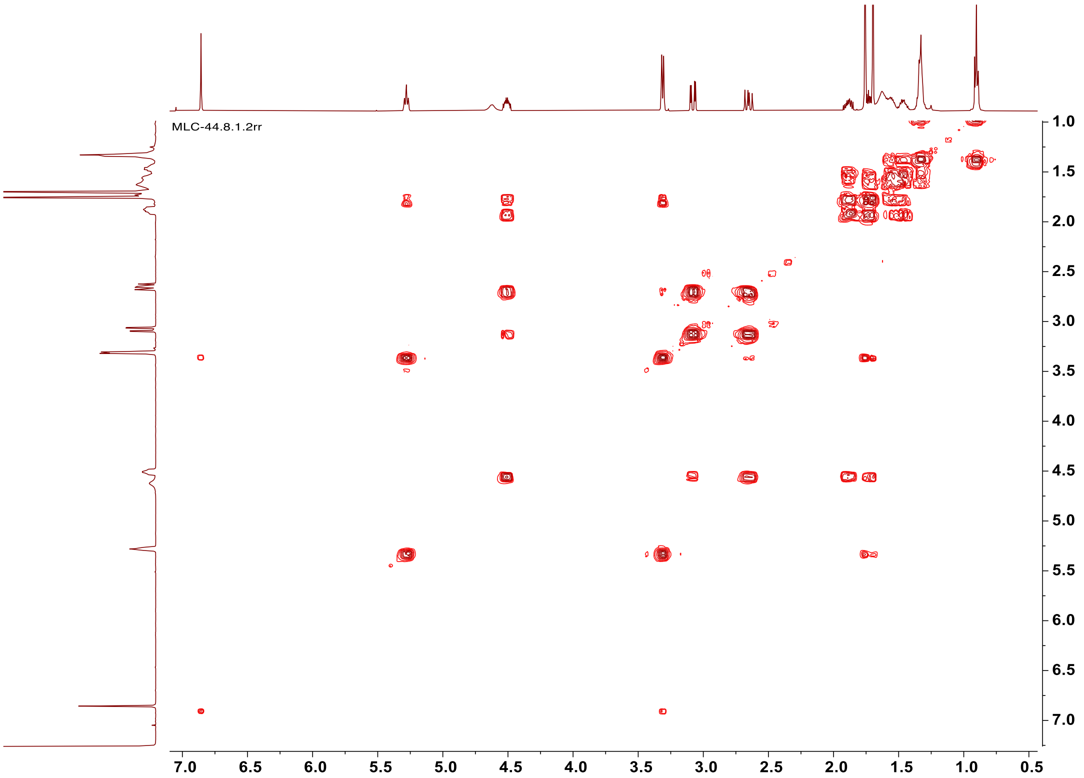

Supplementary Figure 4. ^{1}H-^{1}H COSY spectrum of (±)-**1**.

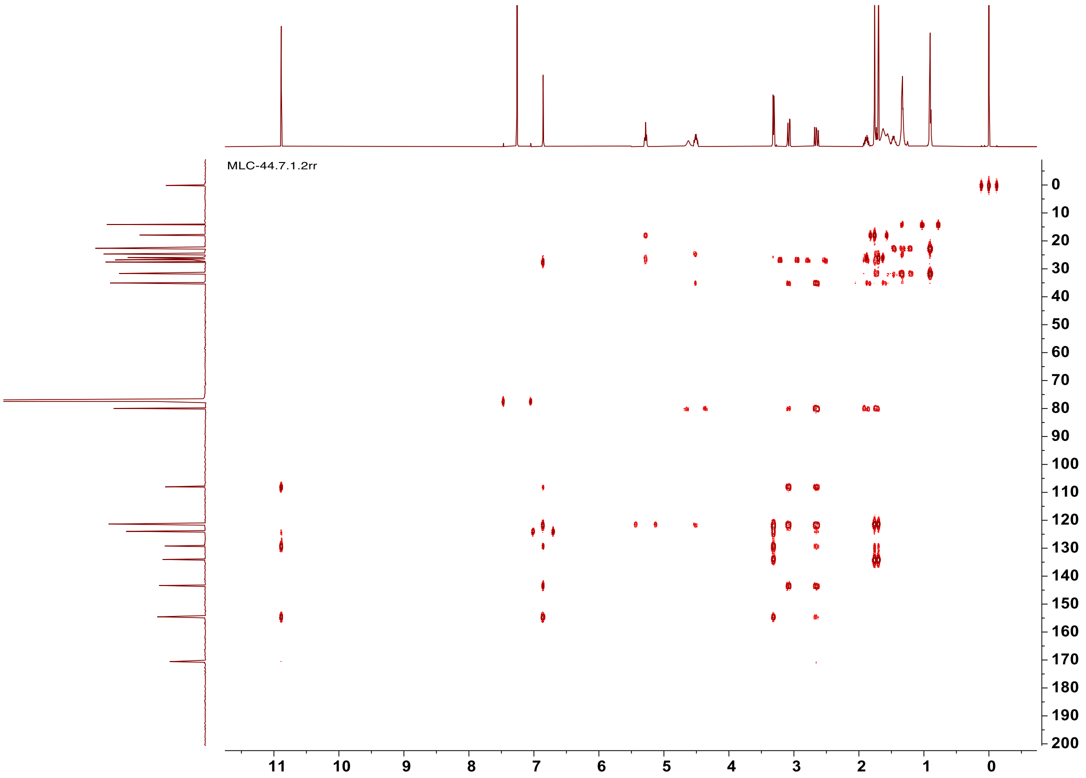

Supplementary Figure 5. HMBC spectrum of (±)-**1**.

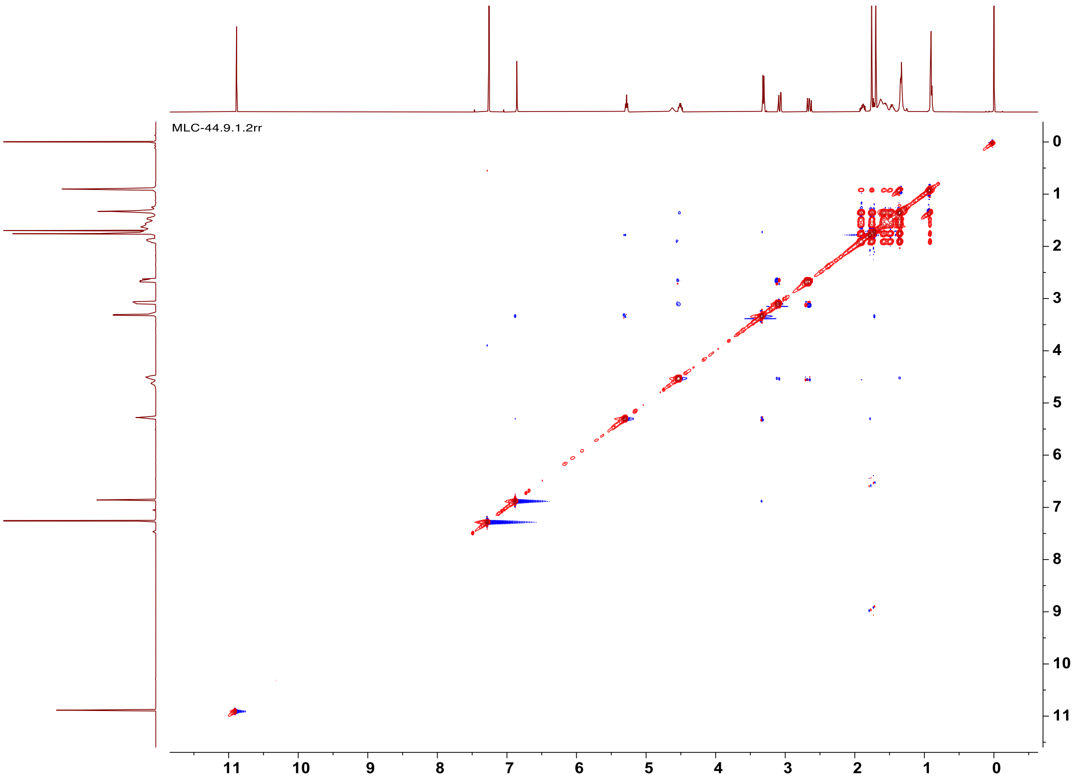

Supplementary Figure 6. ROESY spectrum of (±)-**1**.

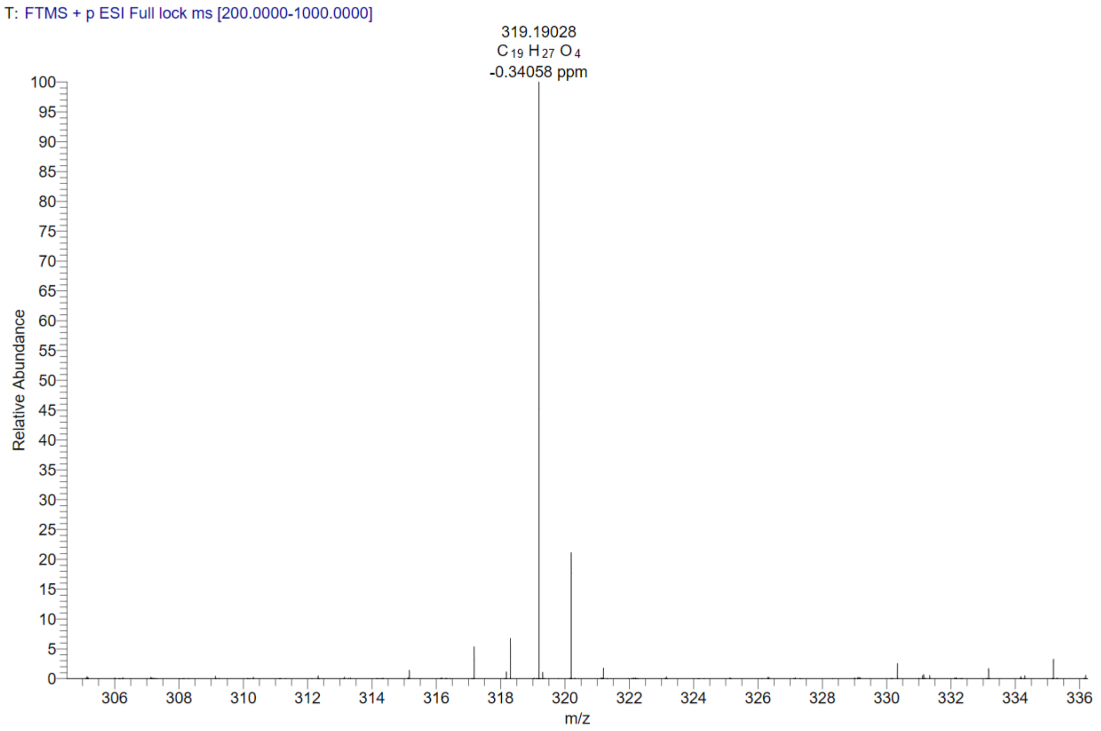

Supplementary Figure 7. HRESIMS report of (±)-**1**.

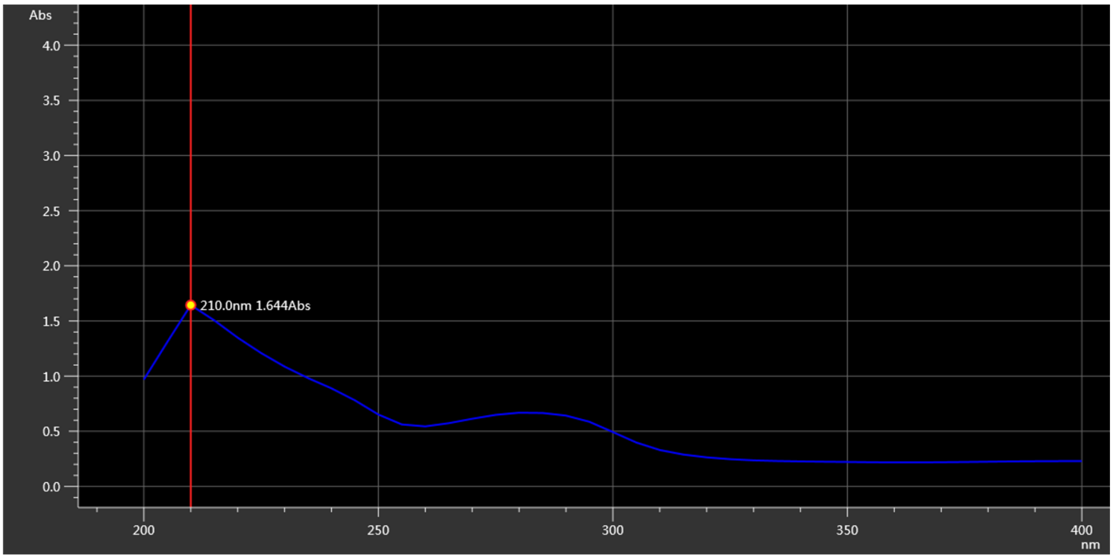

Supplementary Figure 8. UV spectrum of compound (±)-**1**

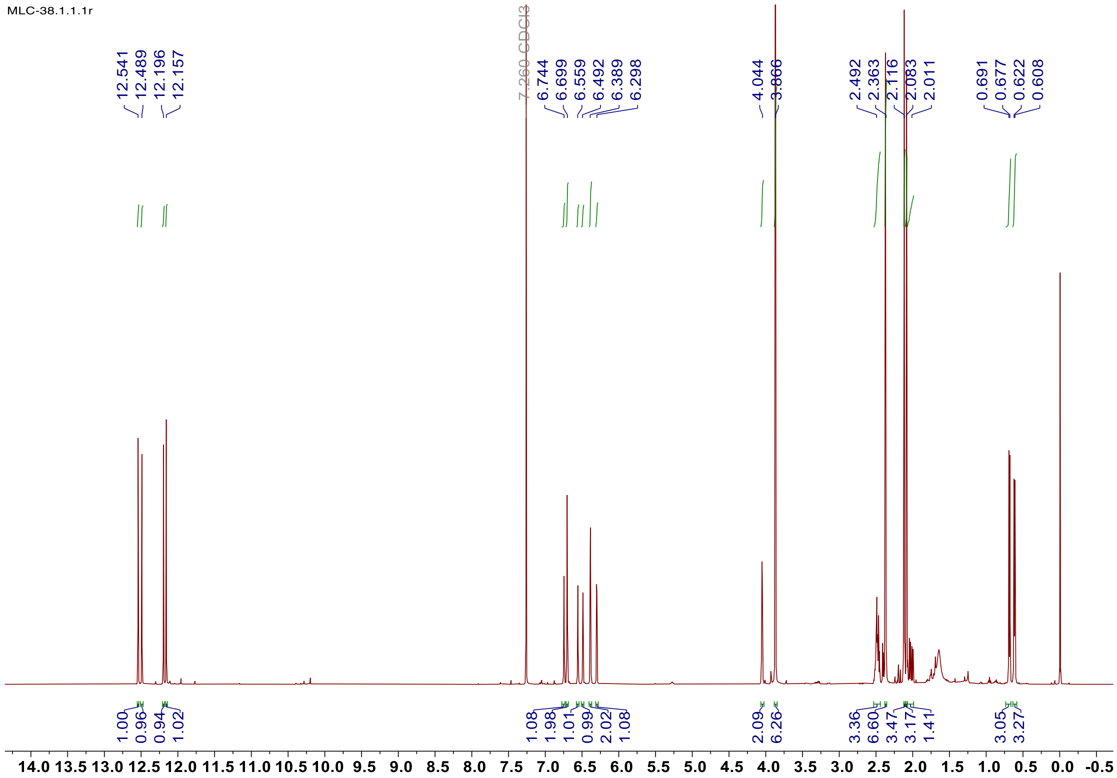

Supplementary Figure 9. ^{1}H NMR spectrum of **2–5** (600 MHz, $CDCl_3$).

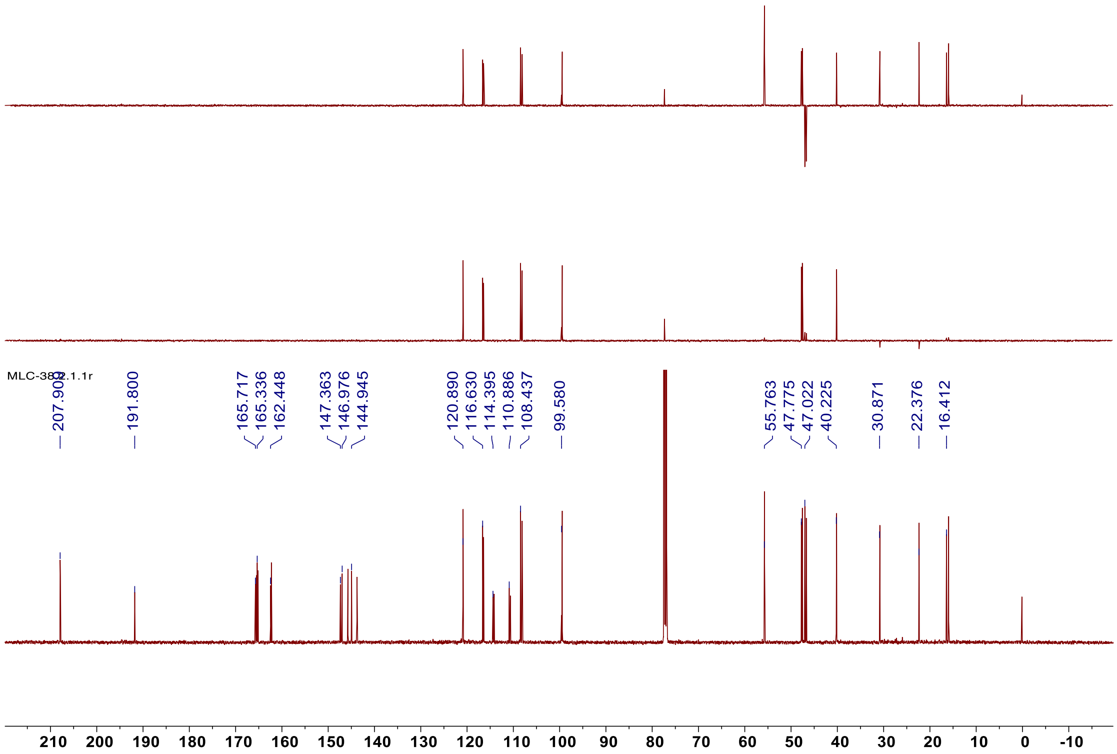

Supplementary Figure 10. ^{13}C and DEPT NMR spectra of **2–5** (150 MHz, $CDCl_3$).

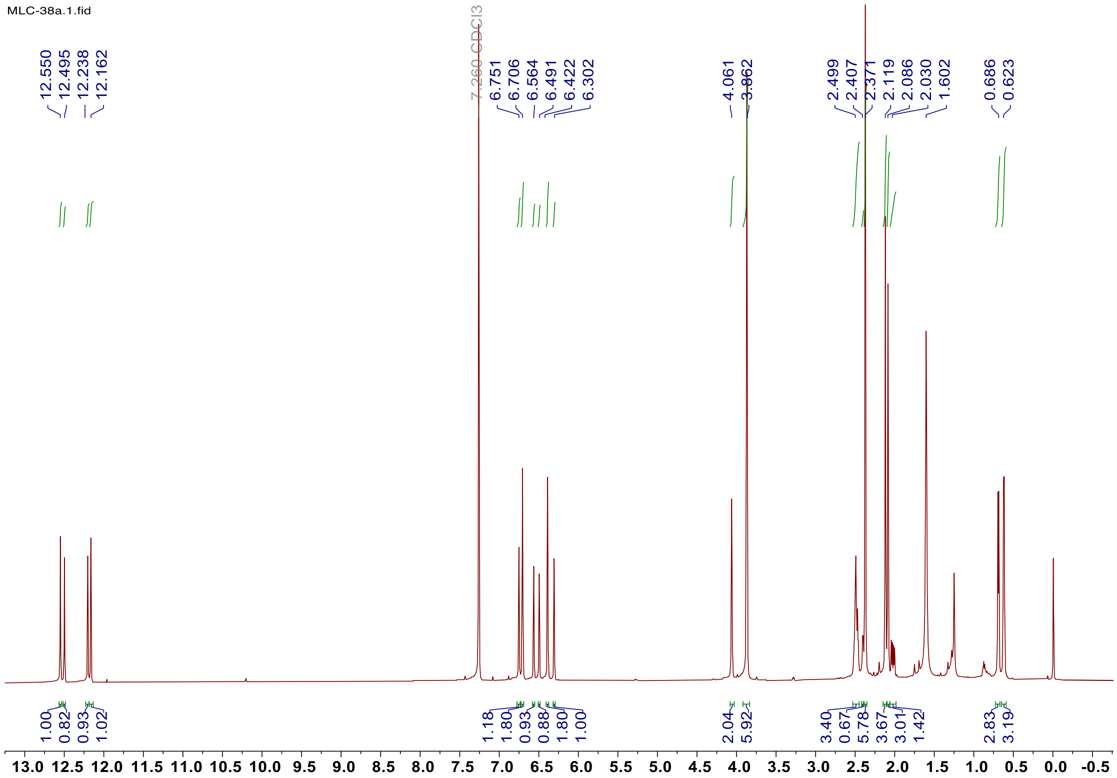

Supplementary Figure 11. ^{1}H NMR spectrum of **4**, **5** (600 MHz, $CDCl_3$) after first separation.

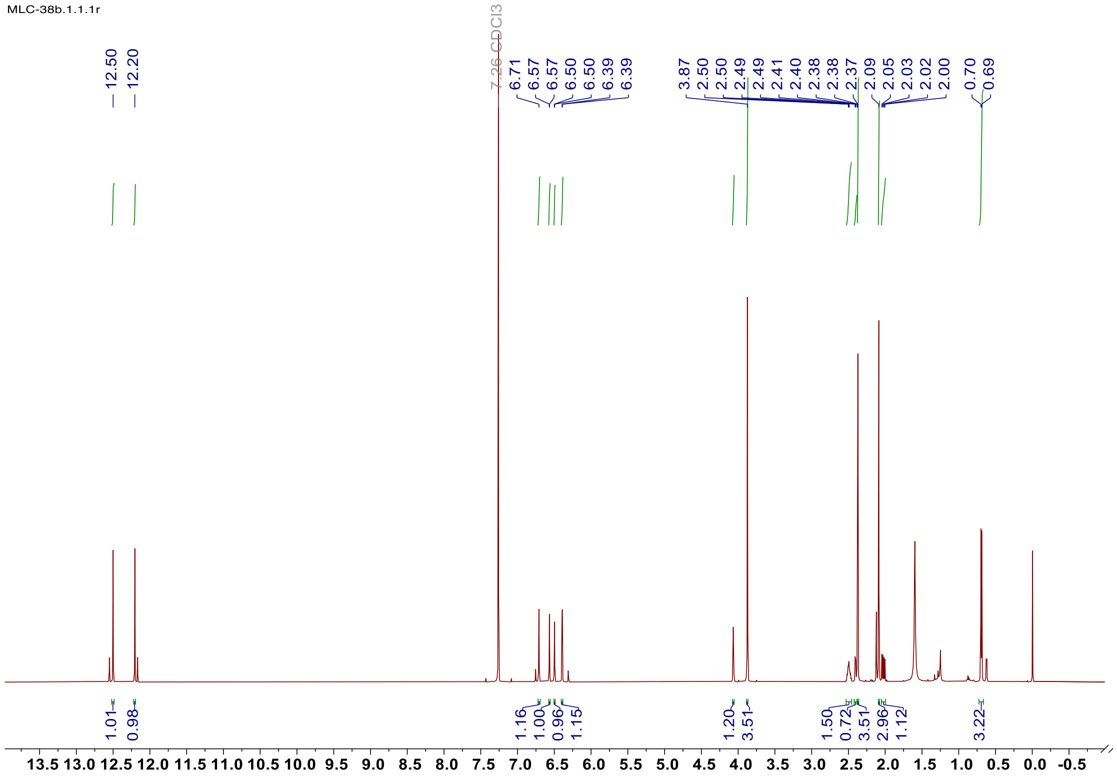

Supplementary Figure 12. ^{1}H NMR spectrum of **2** (600 MHz, $CDCl_3$) after first separation.

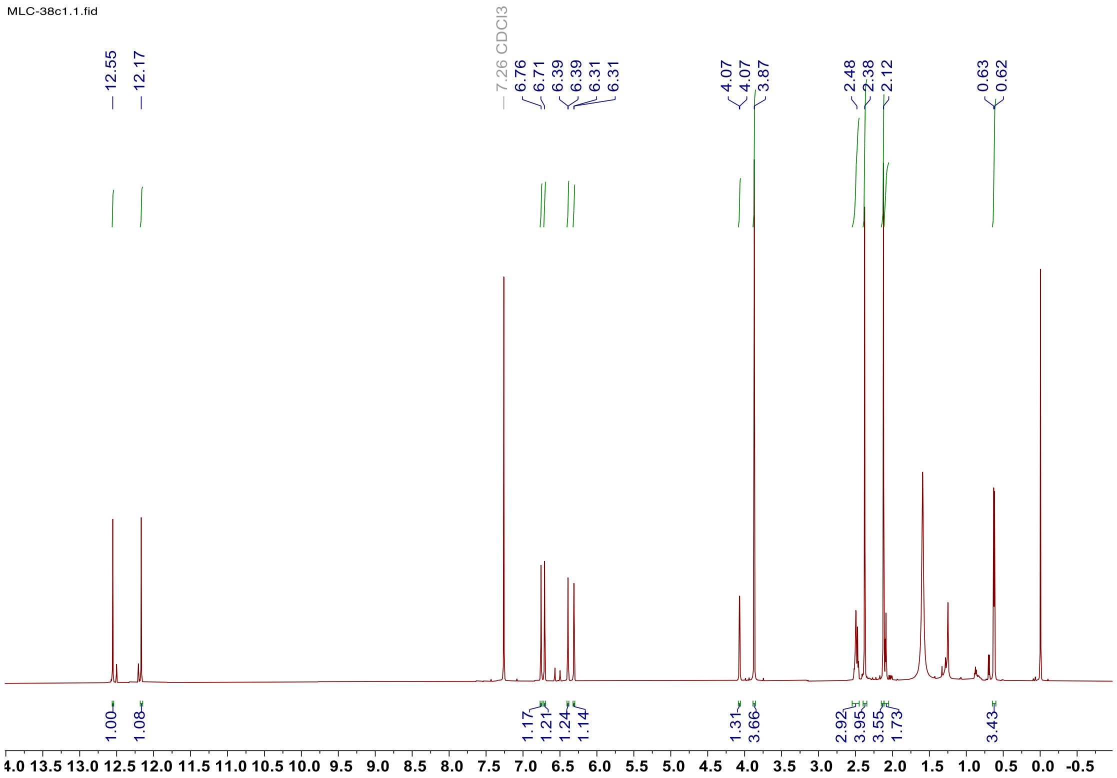

Supplementary Figure 13. ^{1}H NMR spectrum of **3** (600 MHz, $CDCl_3$) after first separation.

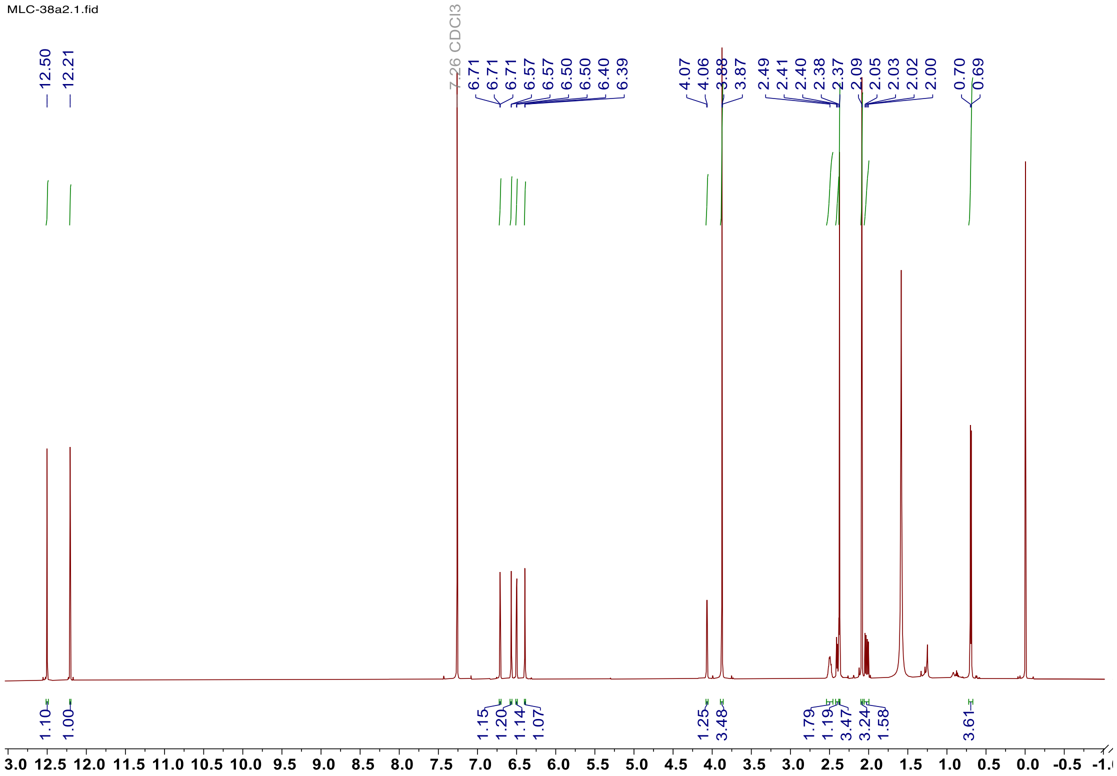

Supplementary Figure 14. ^{1}H NMR spectrum of **4** (600 MHz, $CDCl_3$) after second separation.

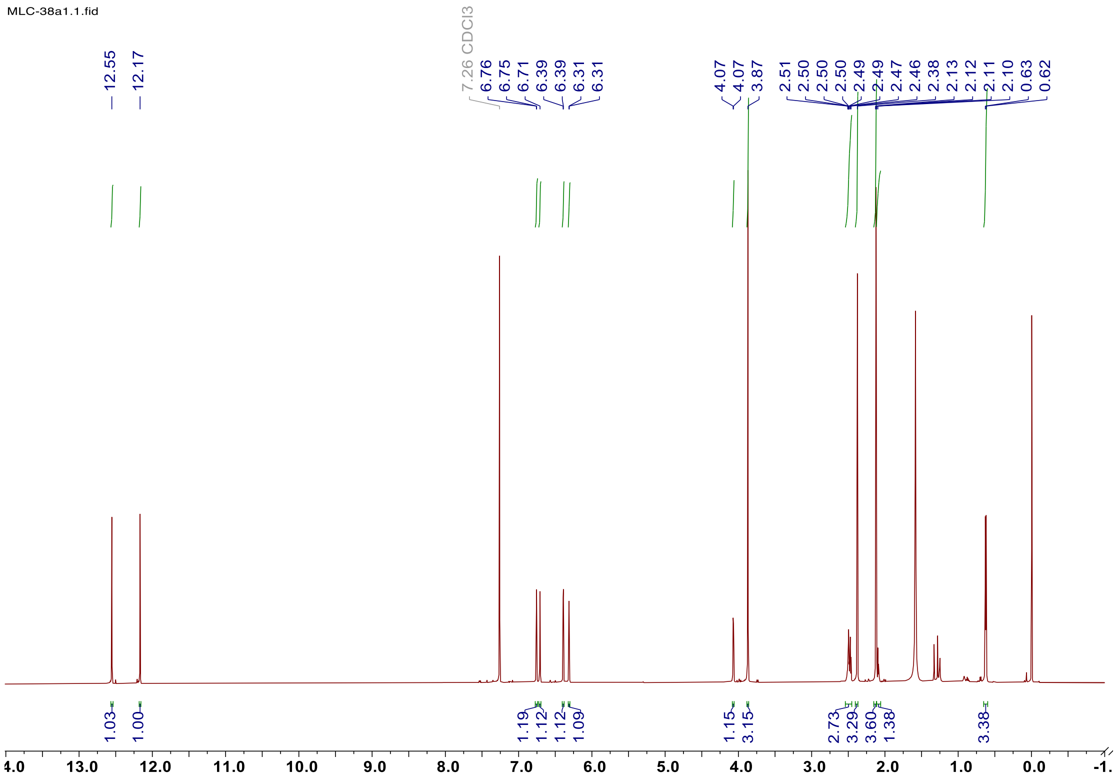

Supplementary Figure 15. ^{1}H NMR spectrum of **5** (600 MHz, $CDCl_3$) after second separation.

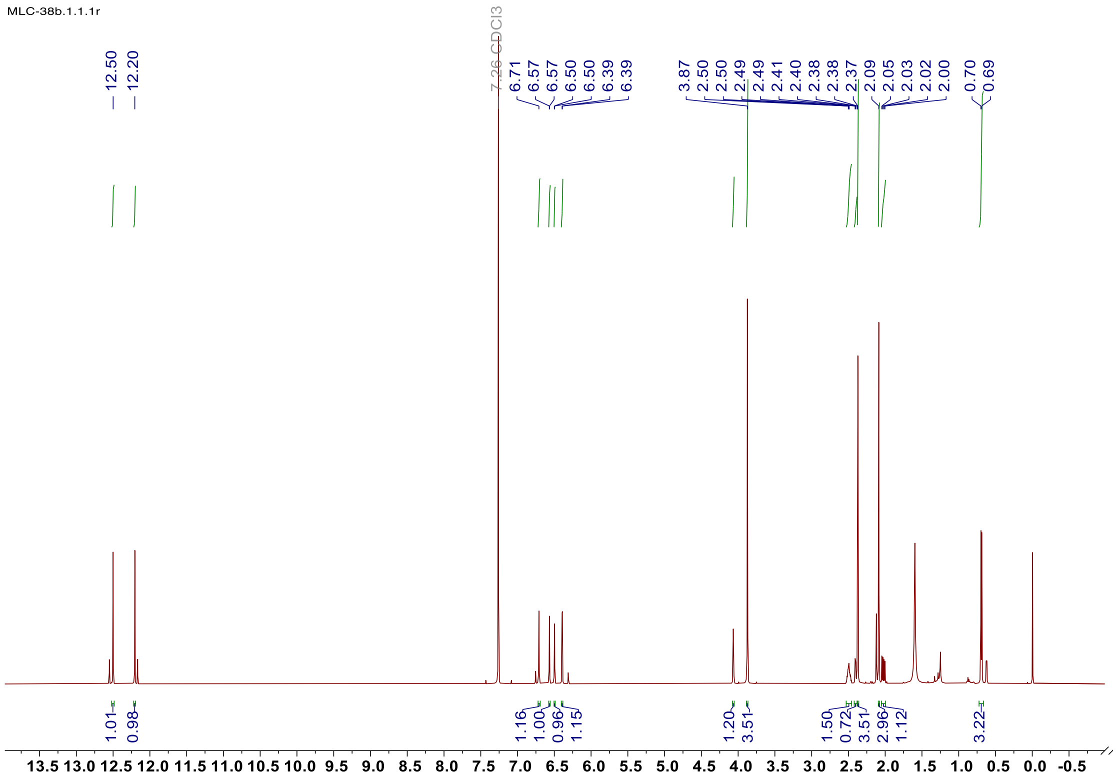

Supplementary Figure 16. ^{1}H NMR spectrum of **2** (600 MHz, $CDCl_3$).

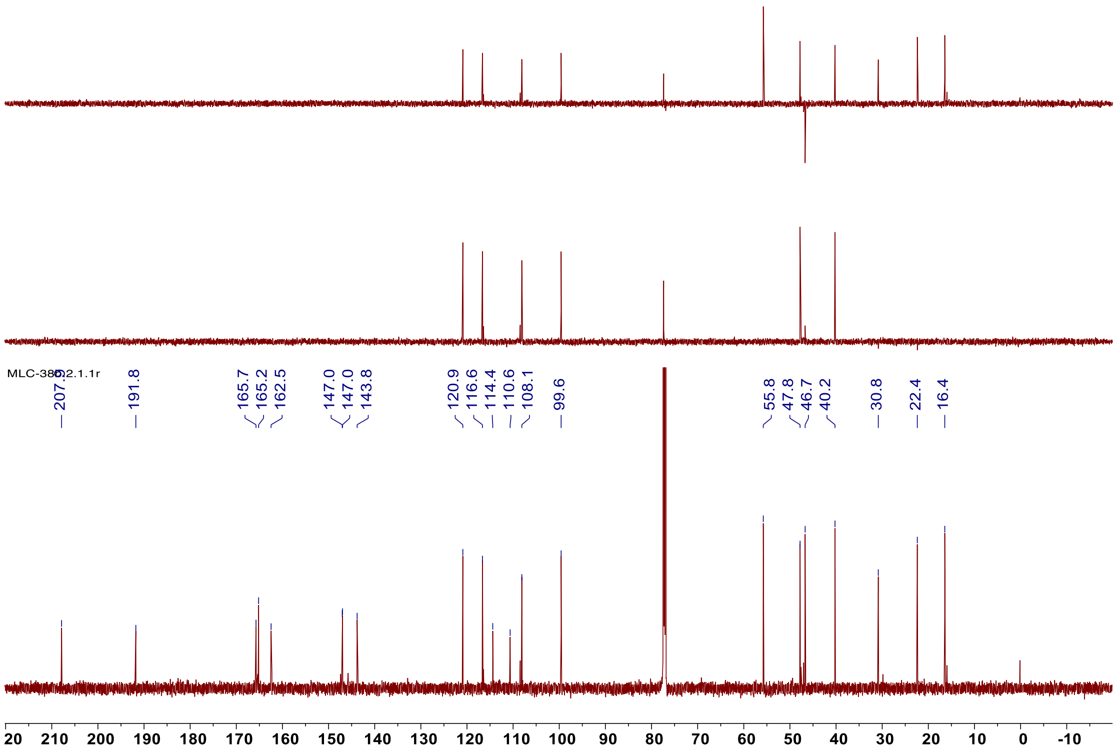

Supplementary Figure 17. ^{13}C and DEPT NMR spectra of **2** (150 MHz, $CDCl_3$).

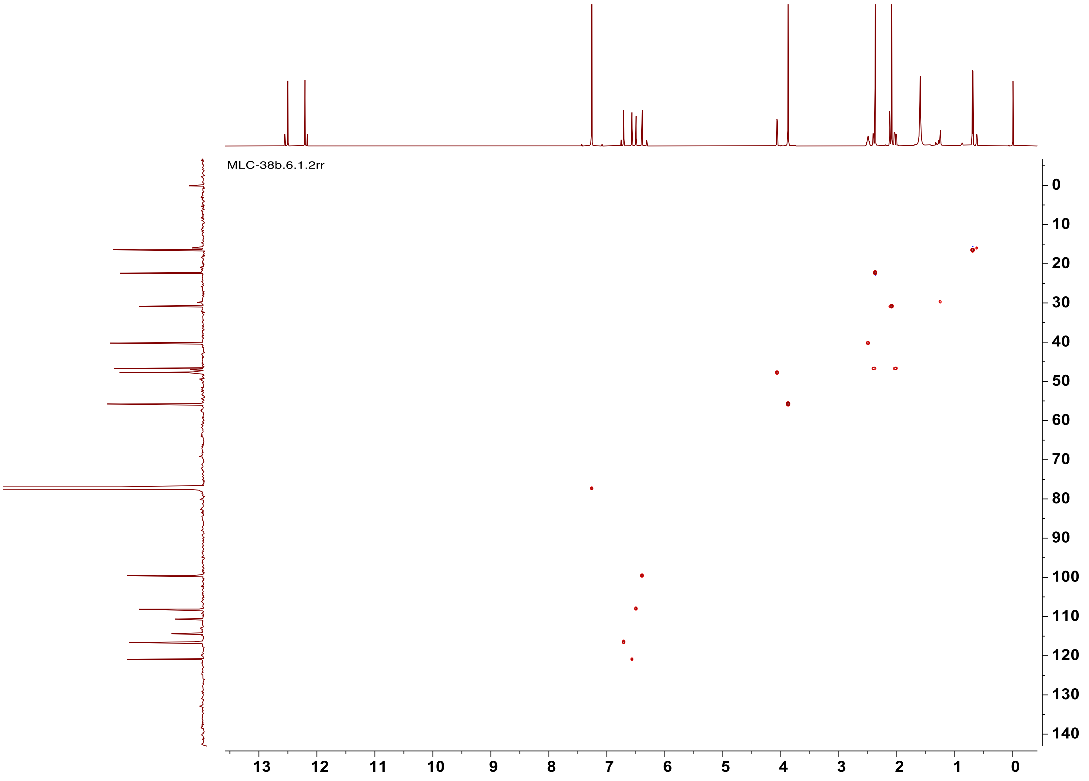

Supplementary Figure 18. HSQC spectrum of **2**.

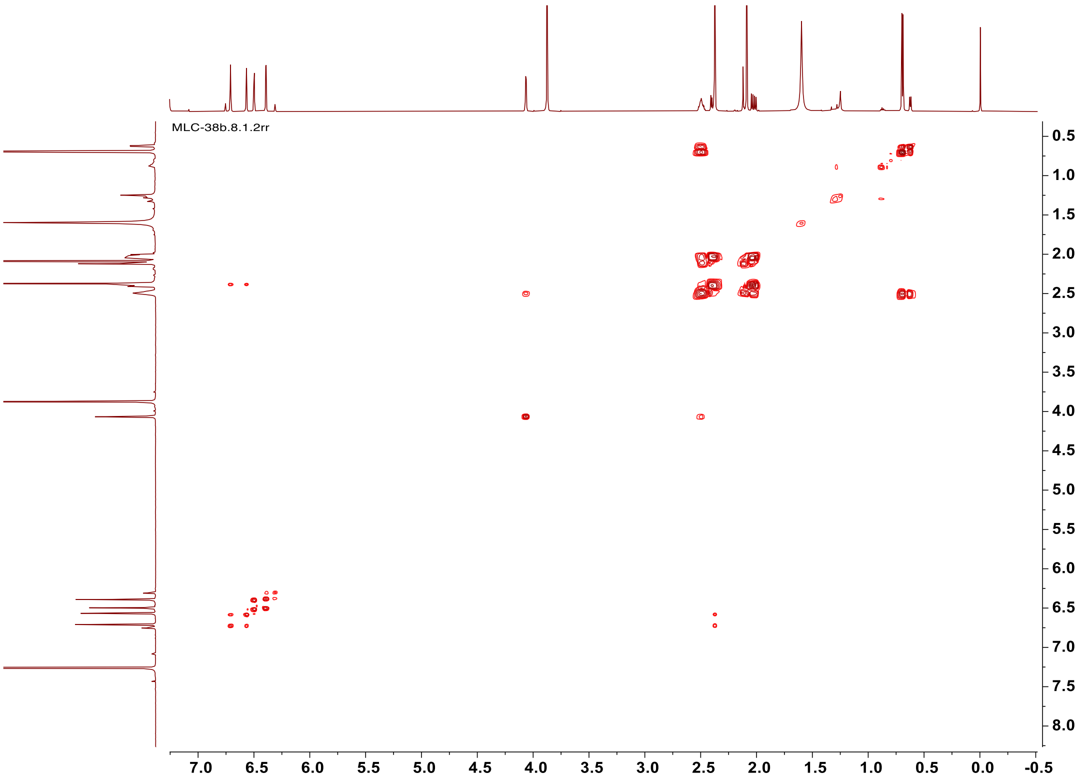

Supplementary Figure 19. ^{1}H-^{1}H COSY spectrum of **2**.

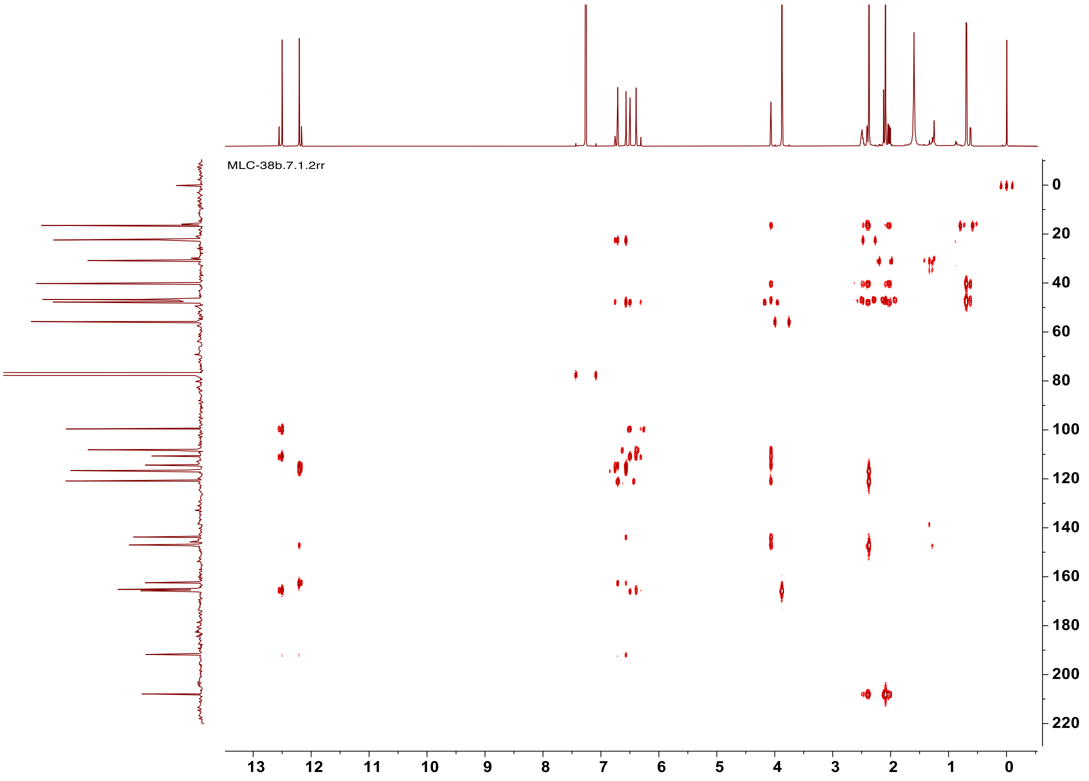

Supplementary Figure 20. HMBC spectrum of **2**.

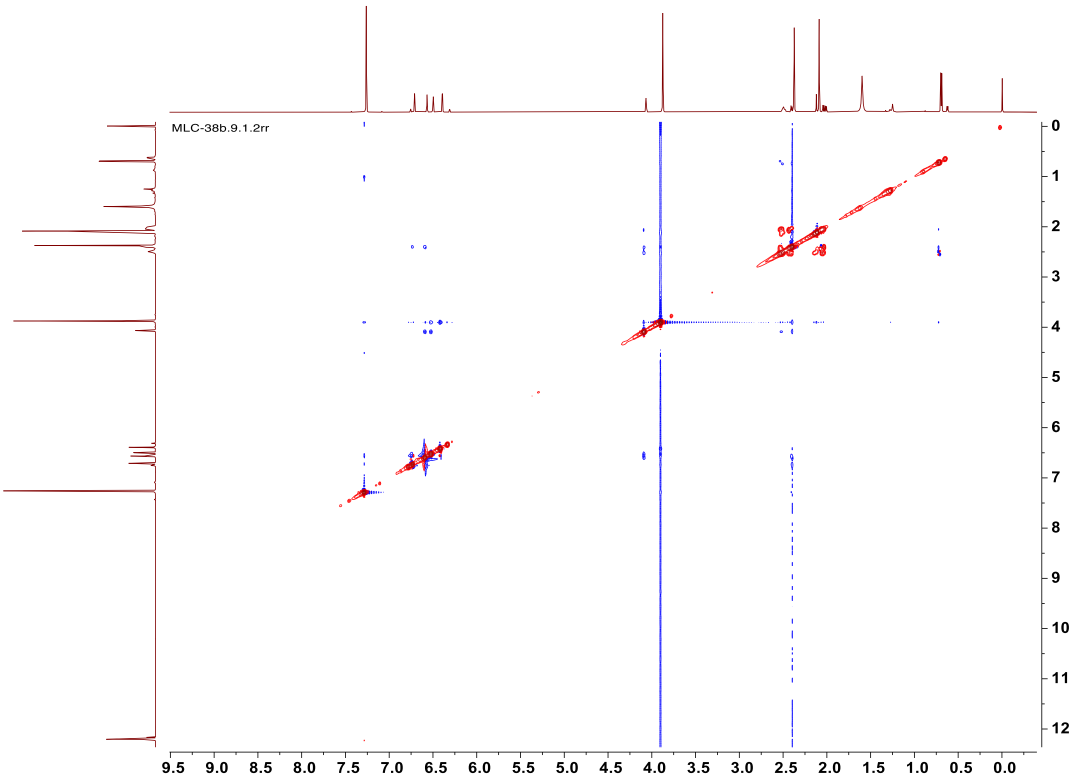

Supplementary Figure 21. ROESY spectrum of **2**.

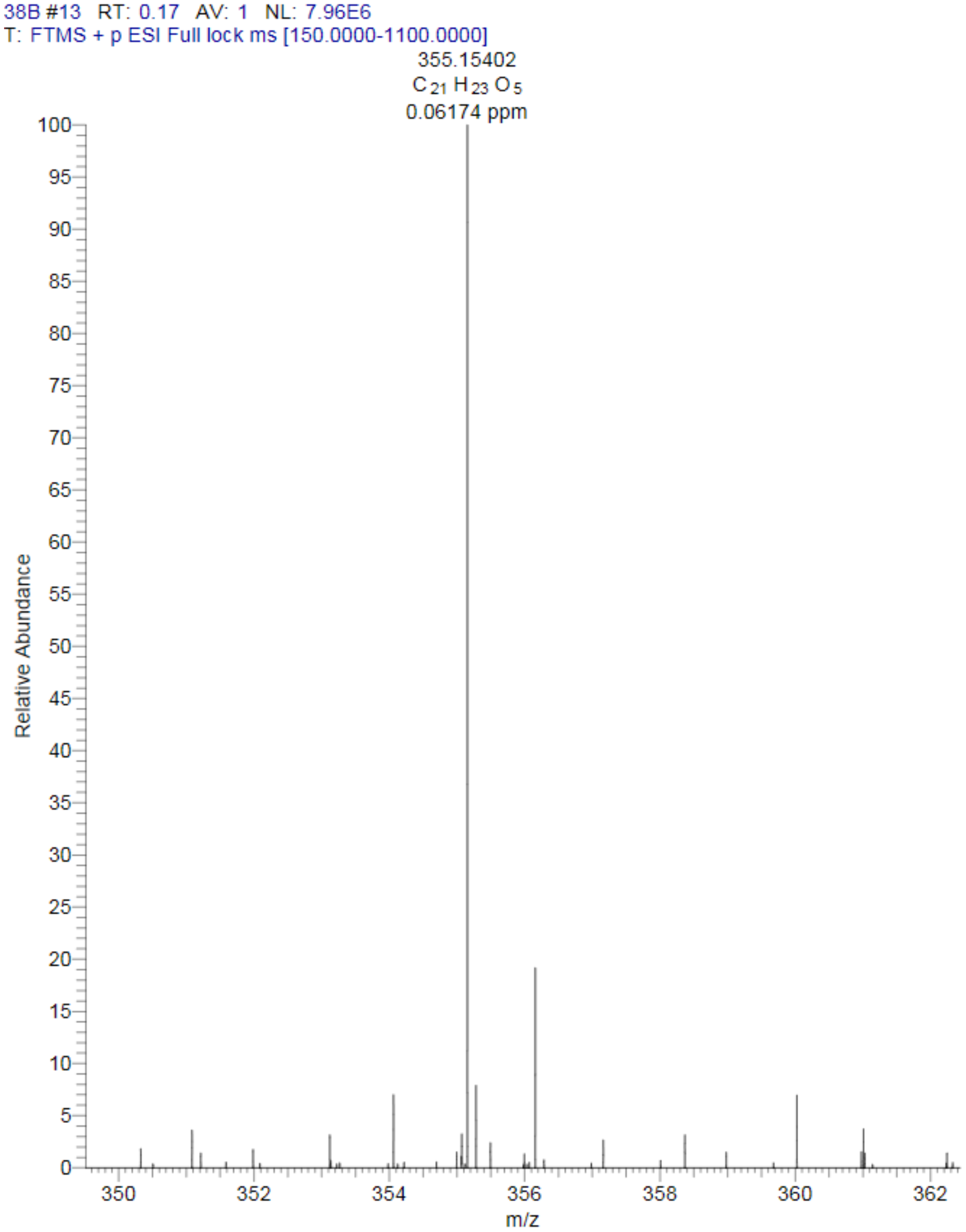

Supplementary Figure 22. HRESIMS report of **2**.

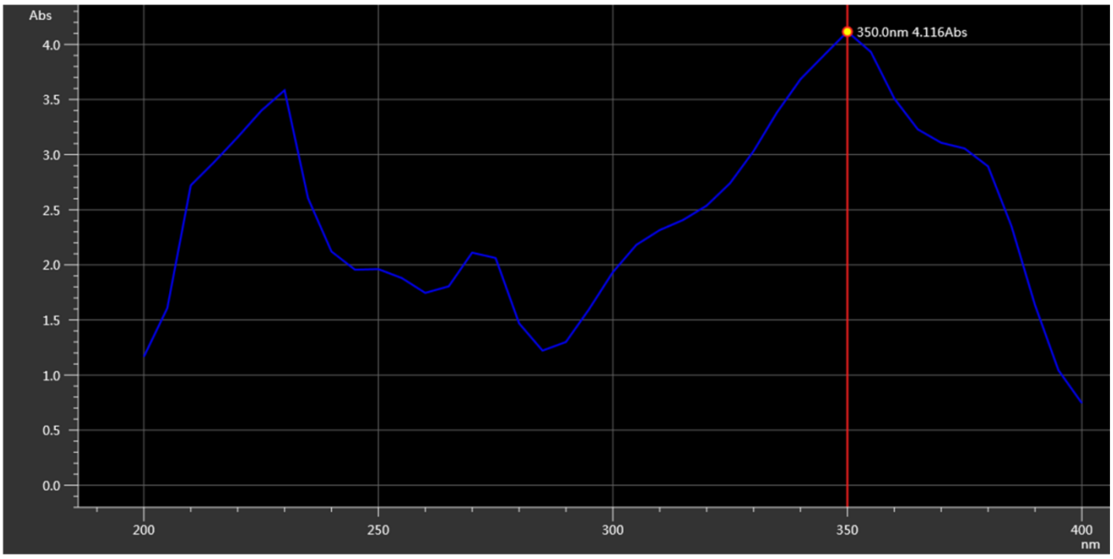

Supplementary Figure 23. UV spectrum of compound **2**.

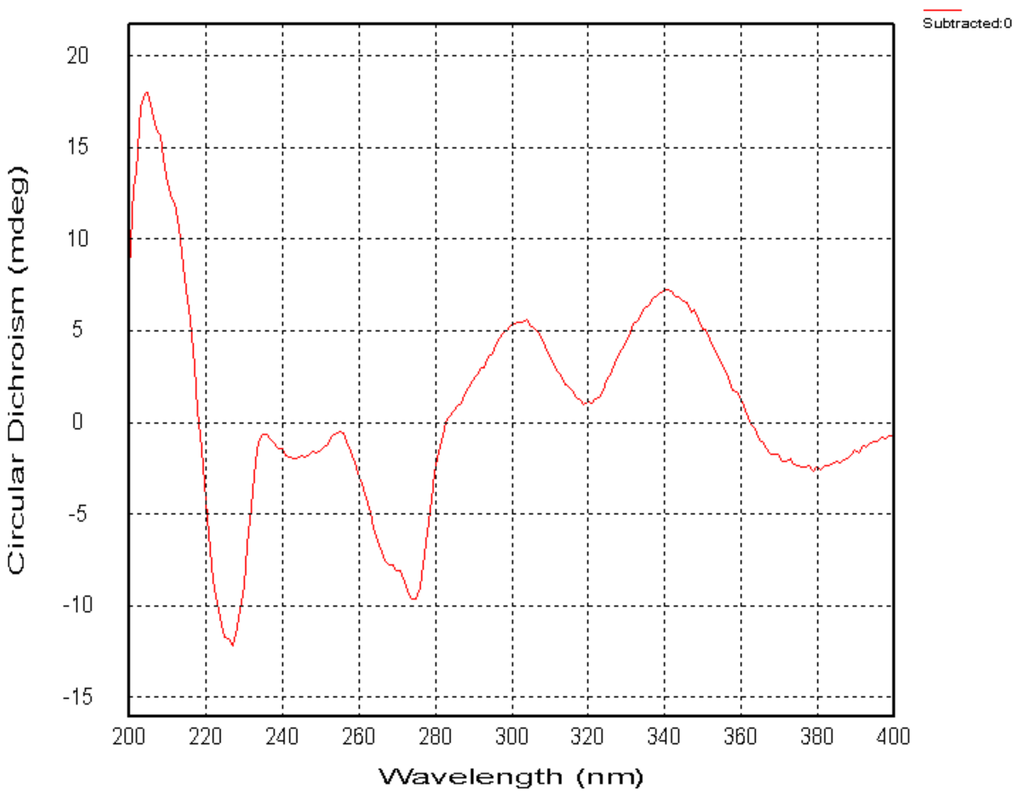

Supplementary Figure 24. CD spectra of compound **2**.

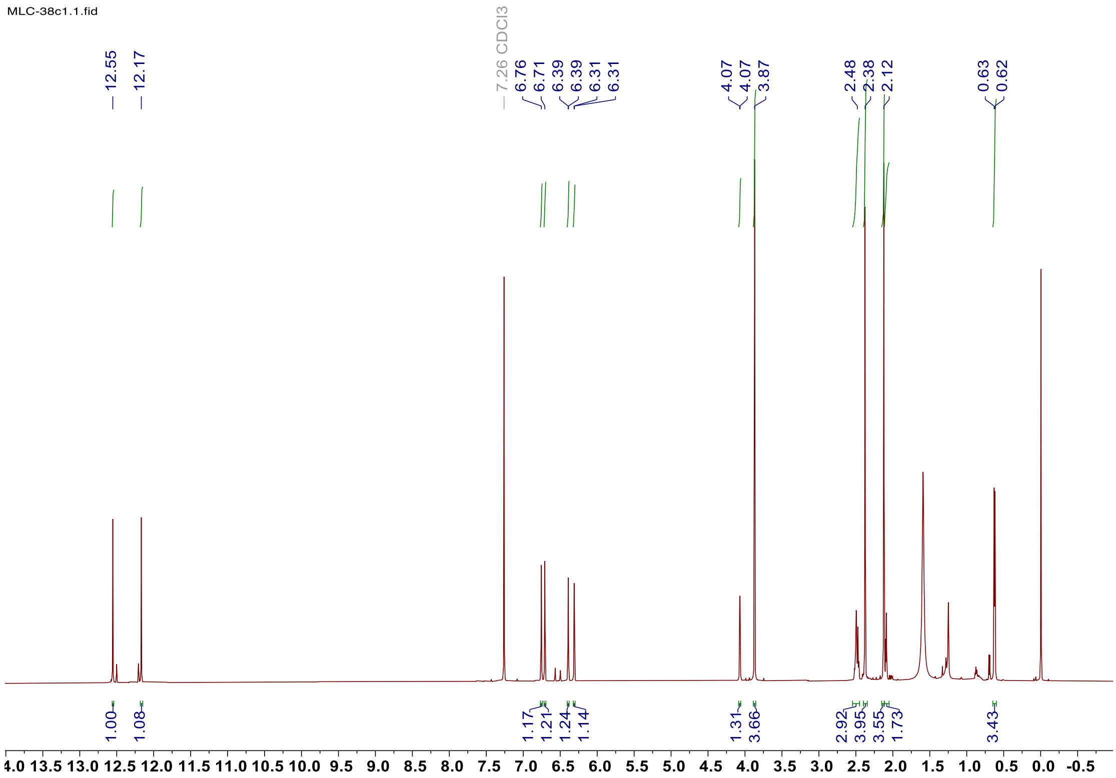

Supplementary Figure 25. ^{1}H NMR spectrum of **3** (600 MHz, $CDCl_3$).

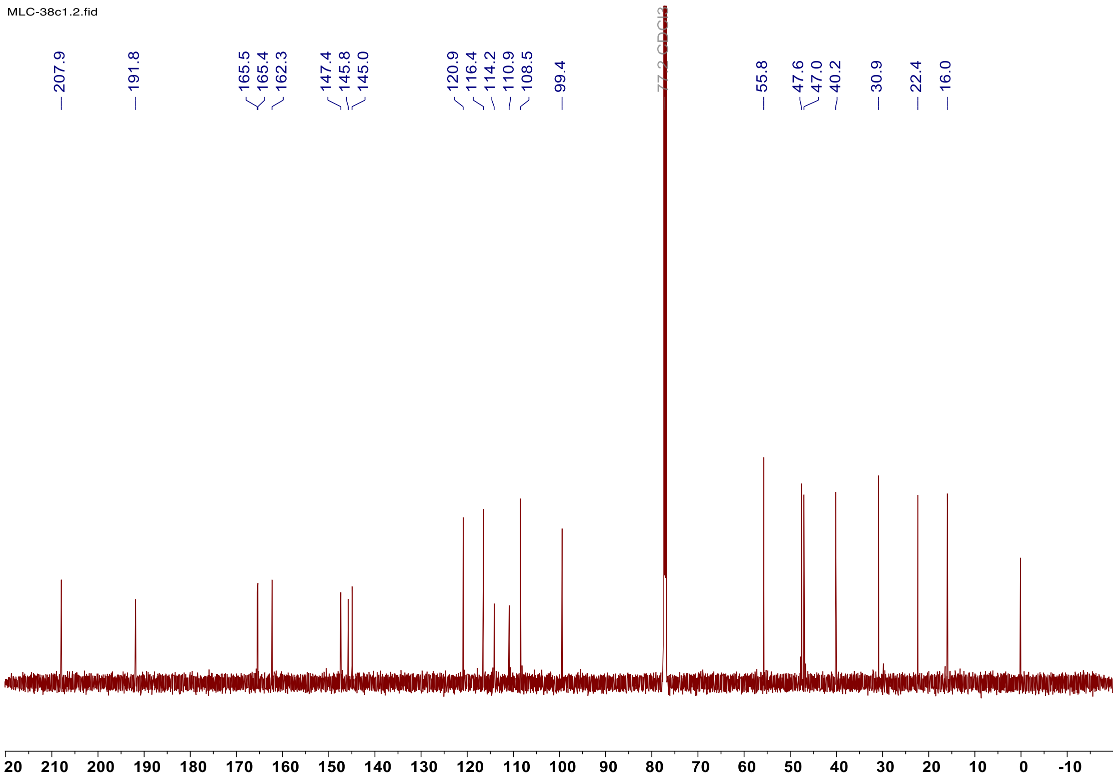

Supplementary Figure 26. ^{13}C NMR spectra of **3** (150 MHz, $CDCl_3$).

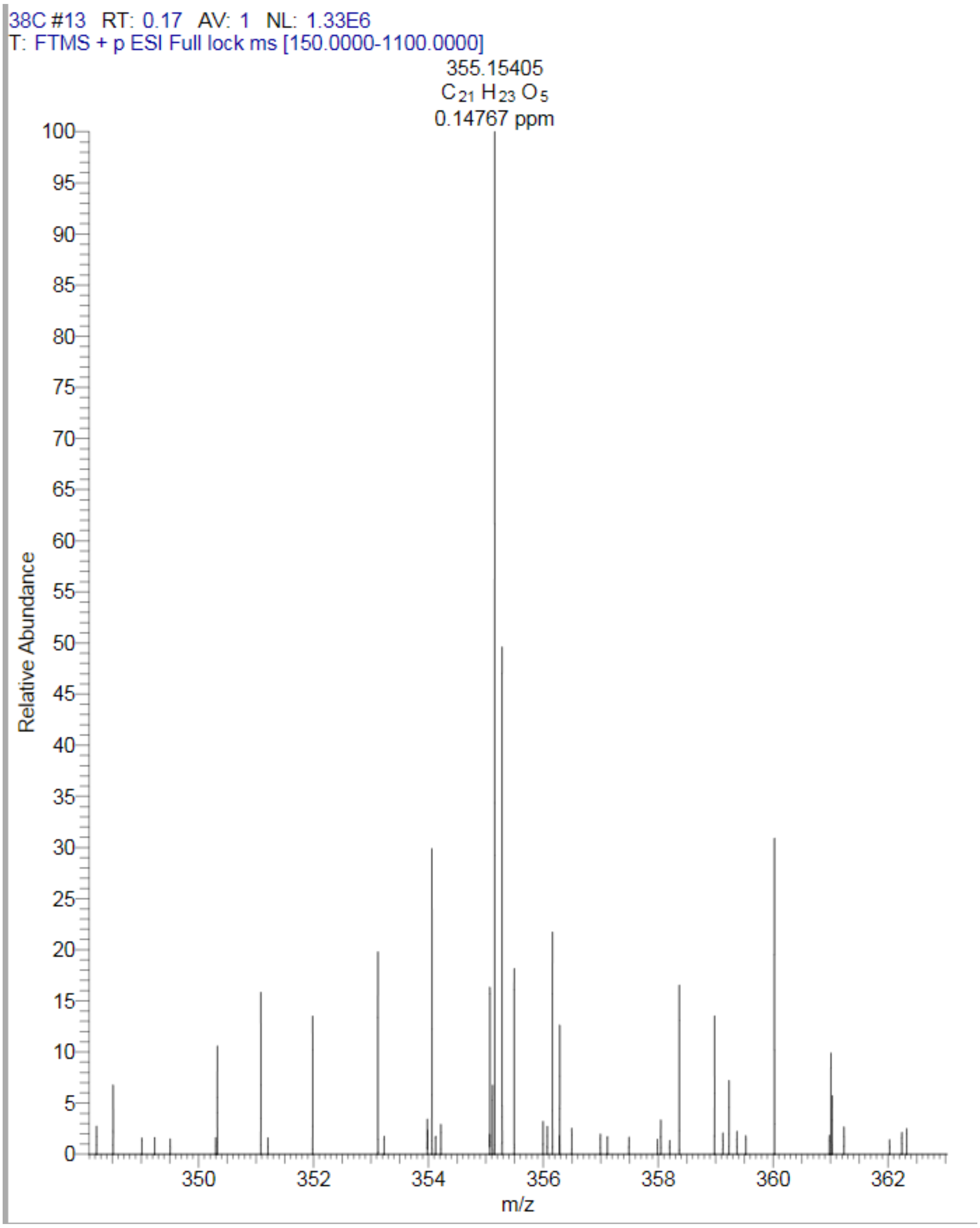

Supplementary Figure 27. HRESIMS report of **3**.

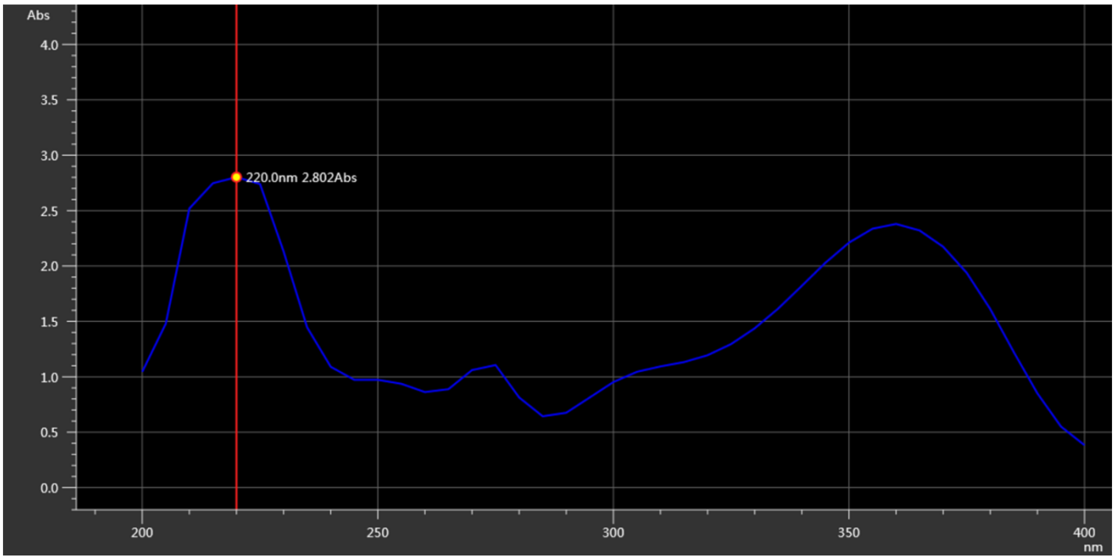

Supplementary Figure 28. UV spectrum of compound **3**.

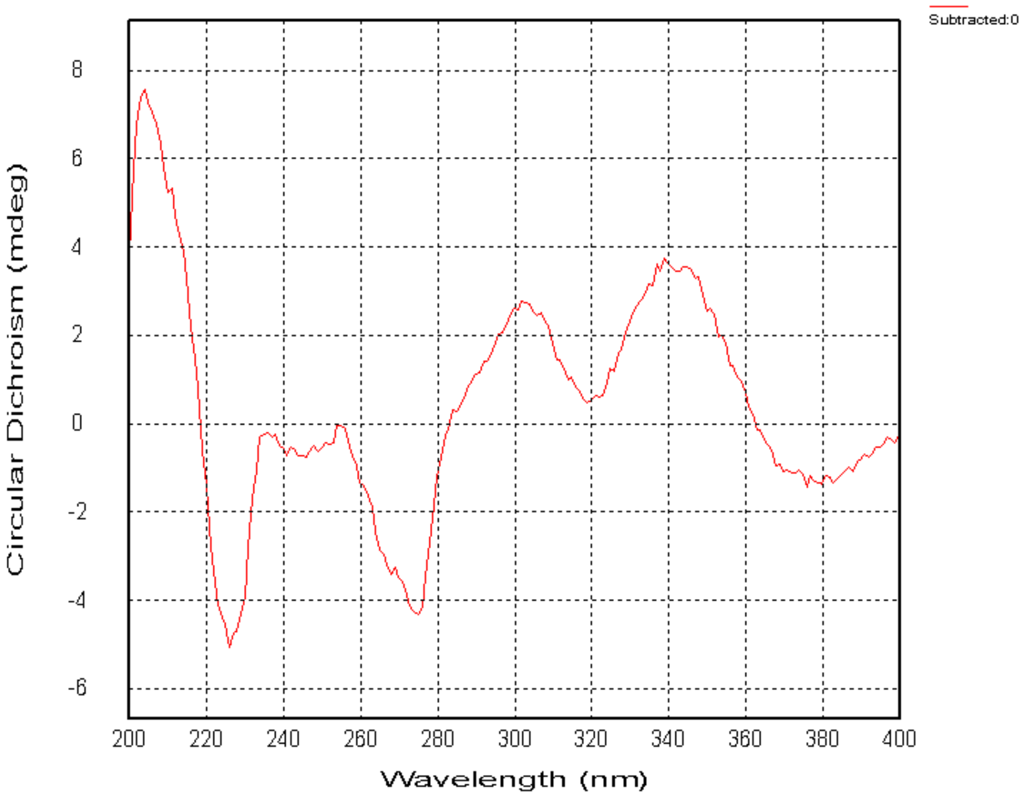

Supplementary Figure 29. CD spectra of compound **3**.

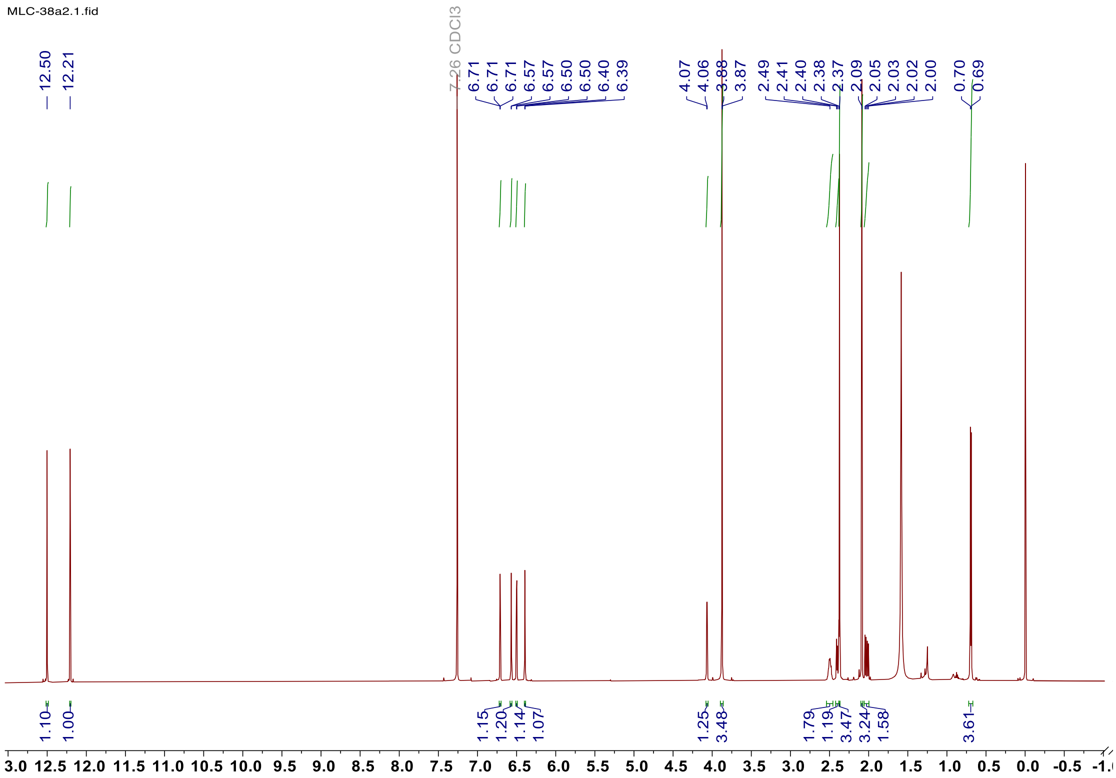

Supplementary Figure 30. ^{1}H NMR spectrum of **4** (600 MHz, $CDCl_3$).

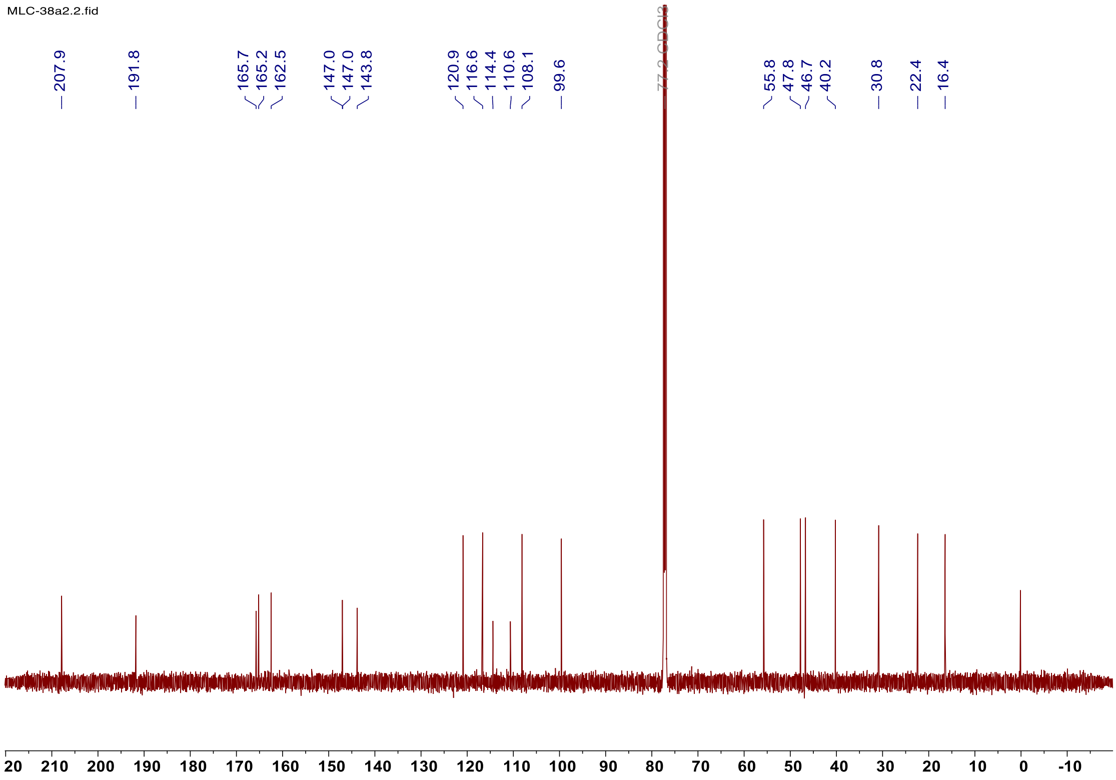

Supplementary Figure 31. ^{13}C NMR spectra of **4** (150 MHz, $CDCl_3$).

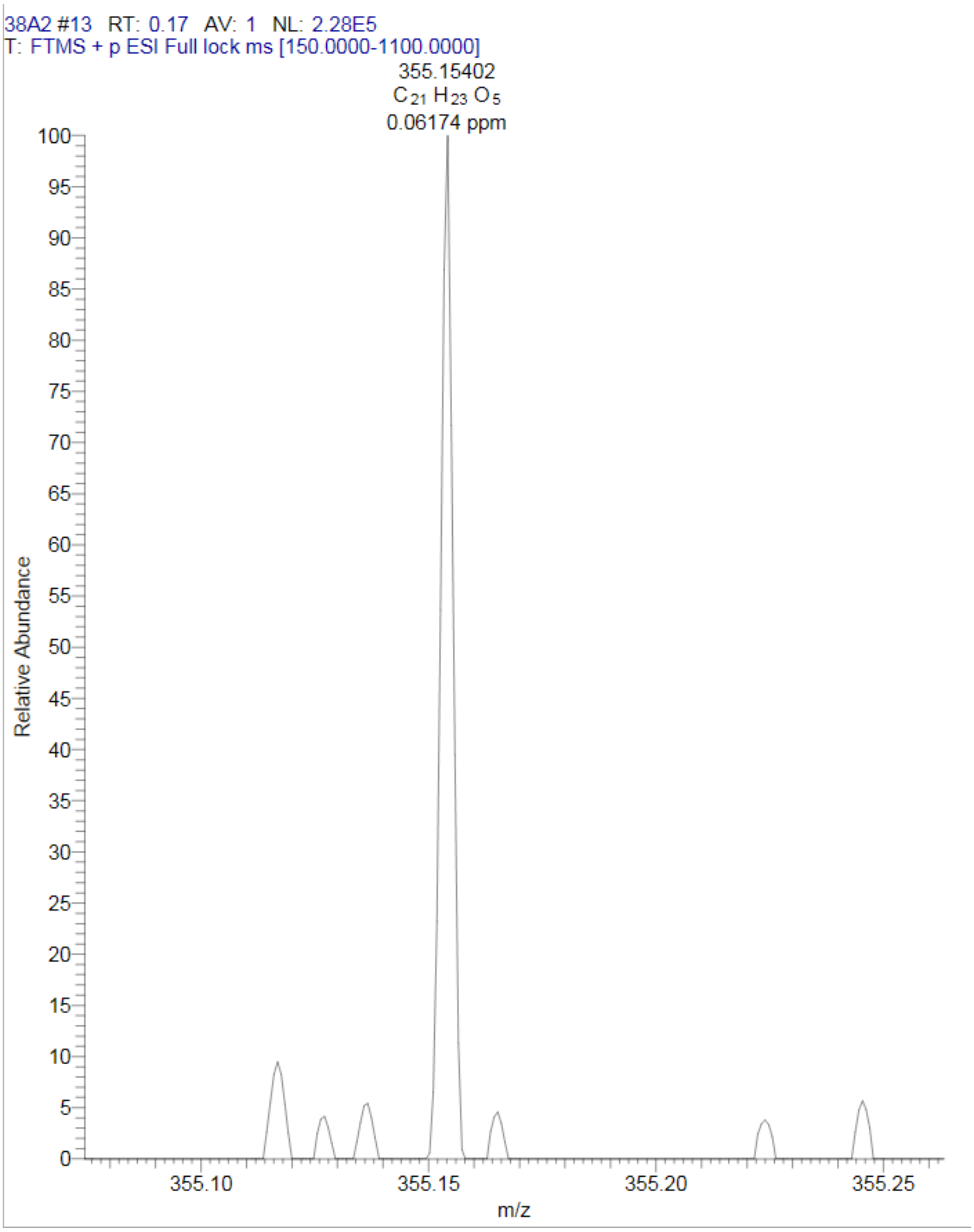

Supplementary Figure 32. HRESIMS report of **4**.

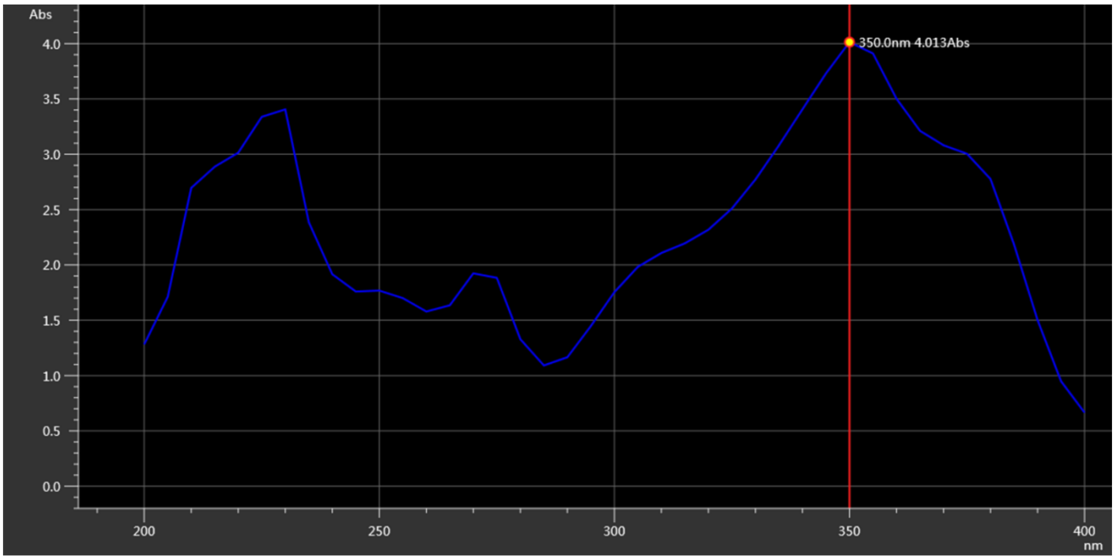

Supplementary Figure 33. UV spectrum of compound **4**.

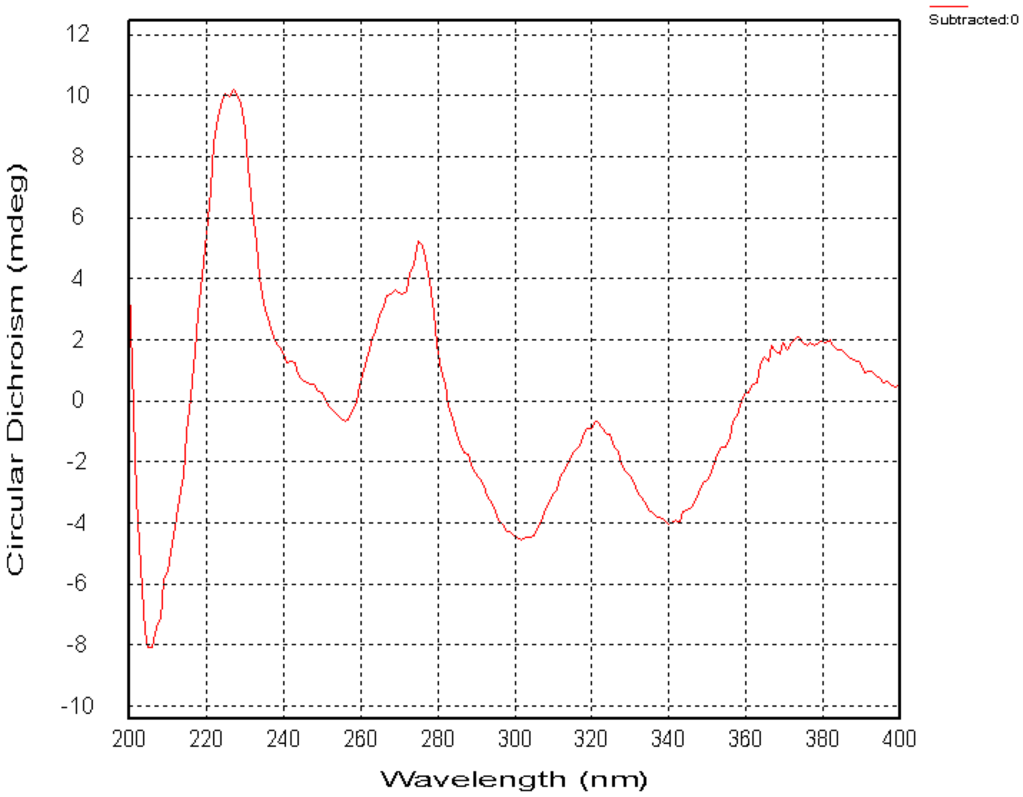

Supplementary Figure 34. CD spectra of compound **4**.

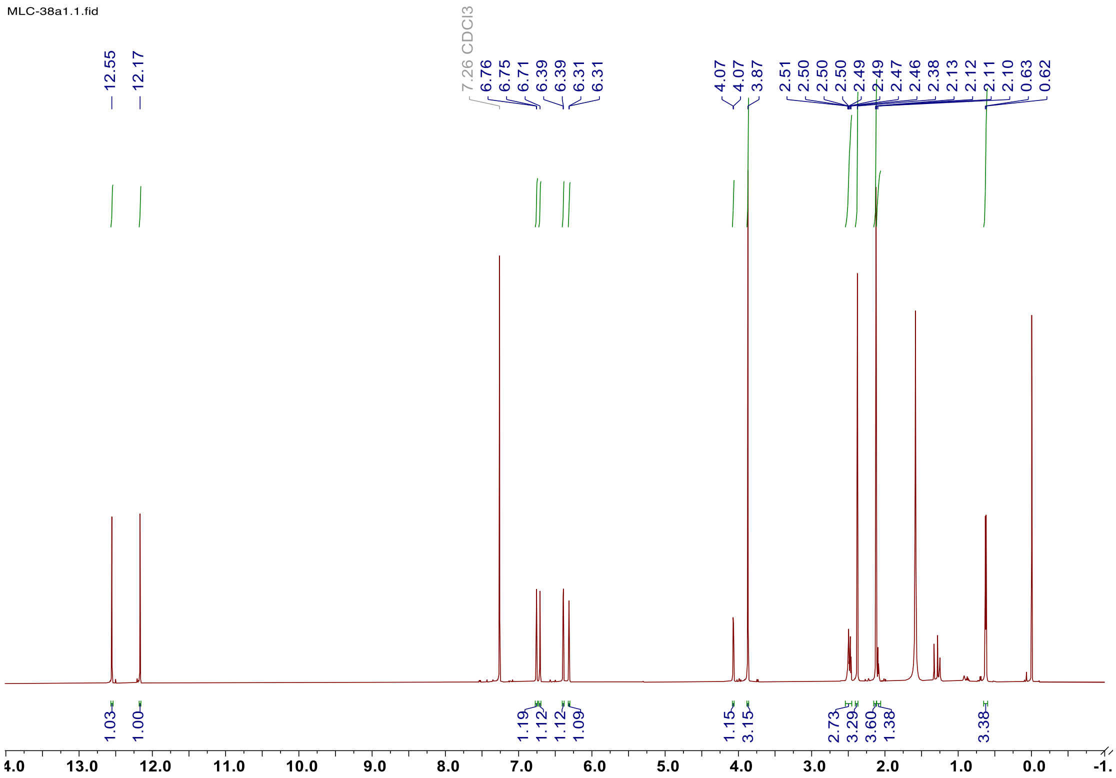

Supplementary Figure 35. ^{1}H NMR spectrum of **5** (600 MHz, $CDCl_3$).

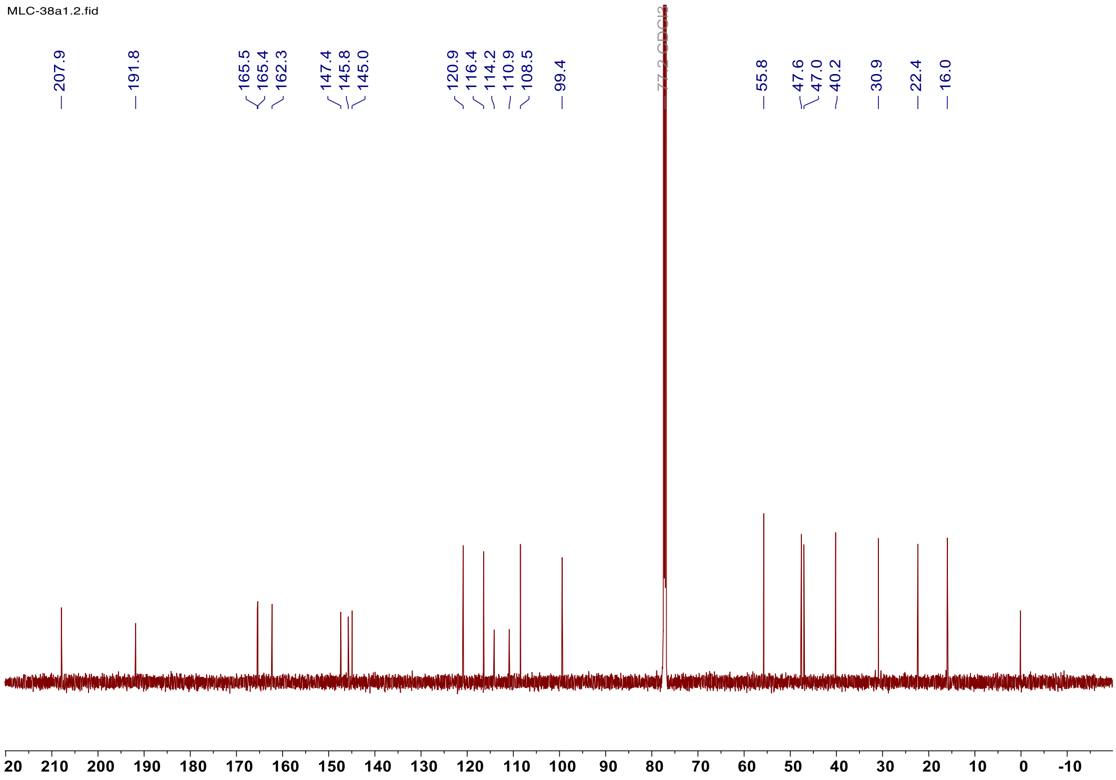

Supplementary Figure 36. ^{13}C NMR spectra of **5** (150 MHz, $CDCl_3$).

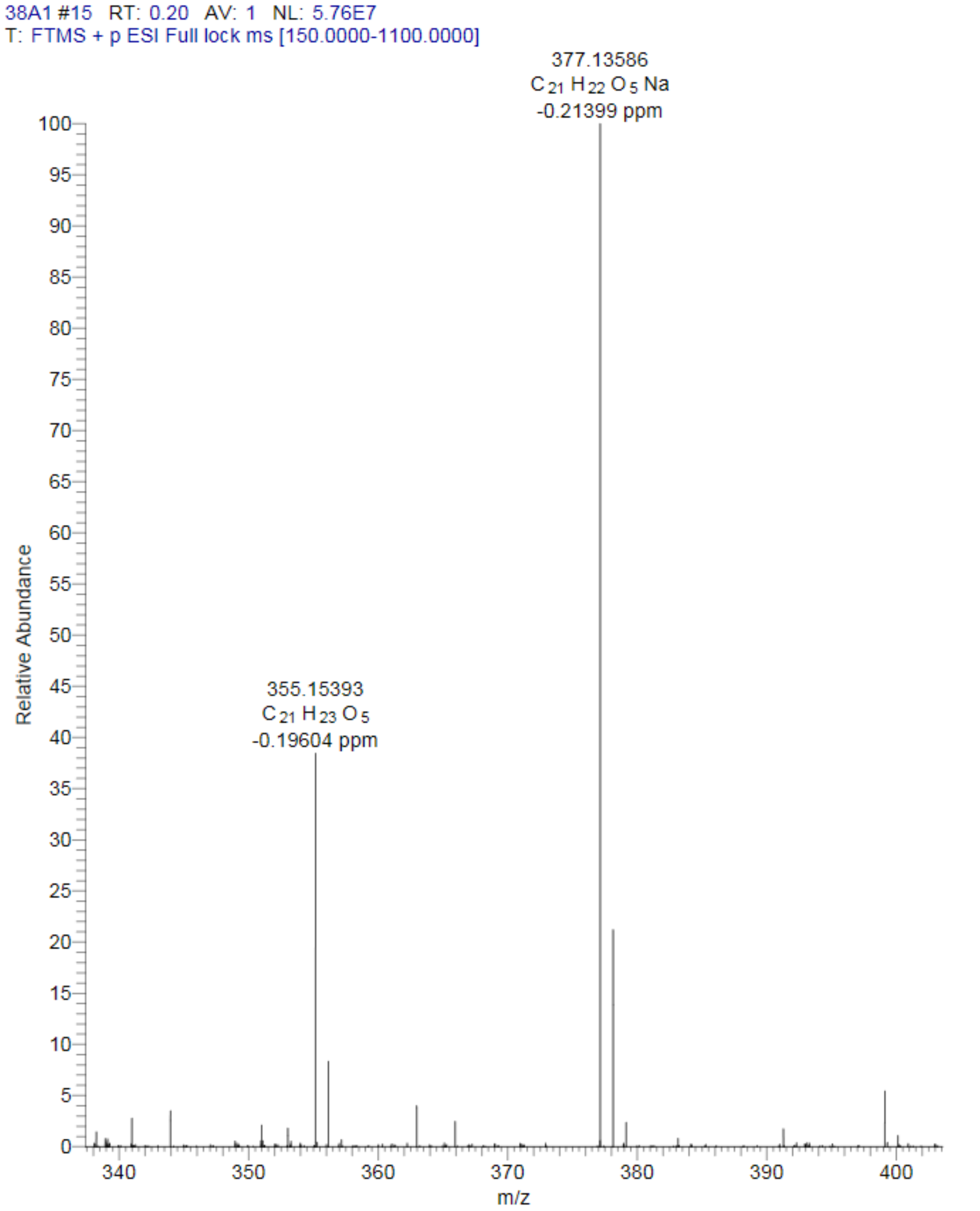

Supplementary Figure 37. HRESIMS report of **5**.

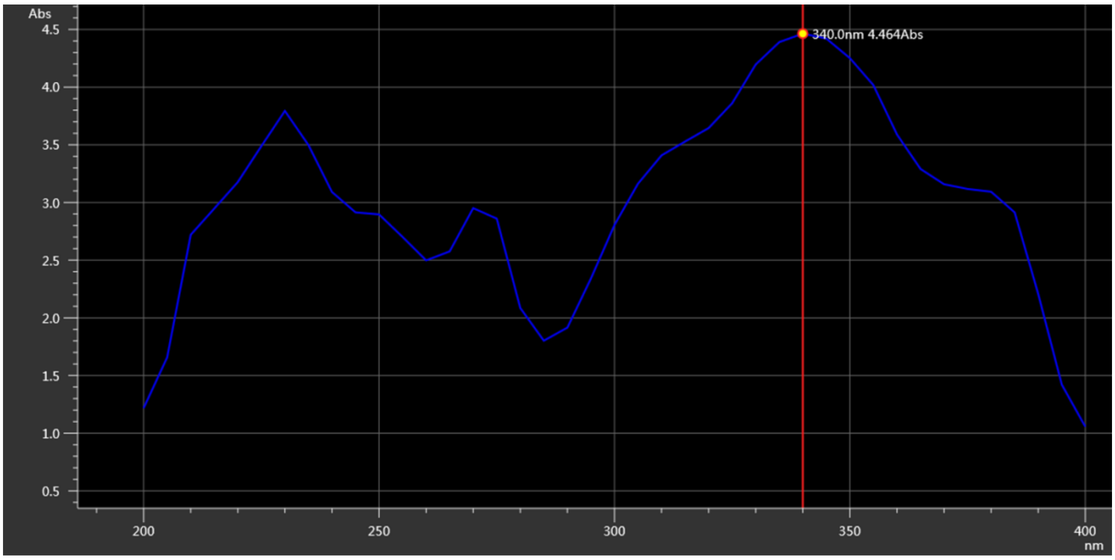

Supplementary Figure 38. UV spectrum of compound **5**.

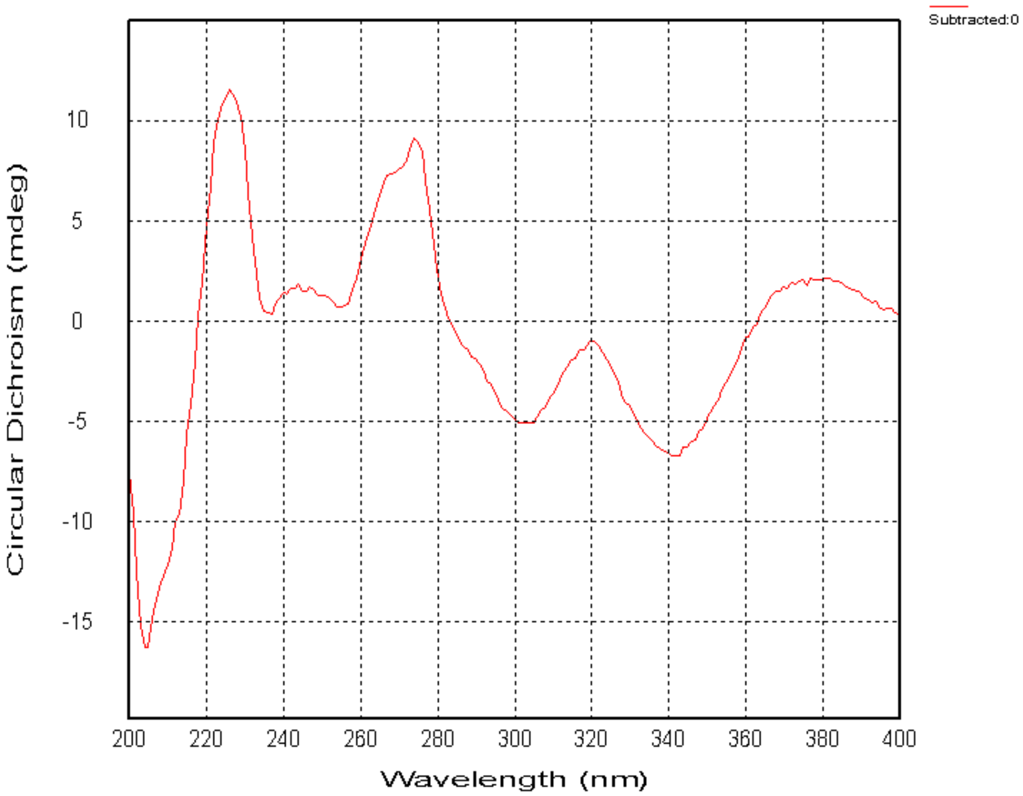

Supplementary Figure 39. CD spectra of compound **5**.

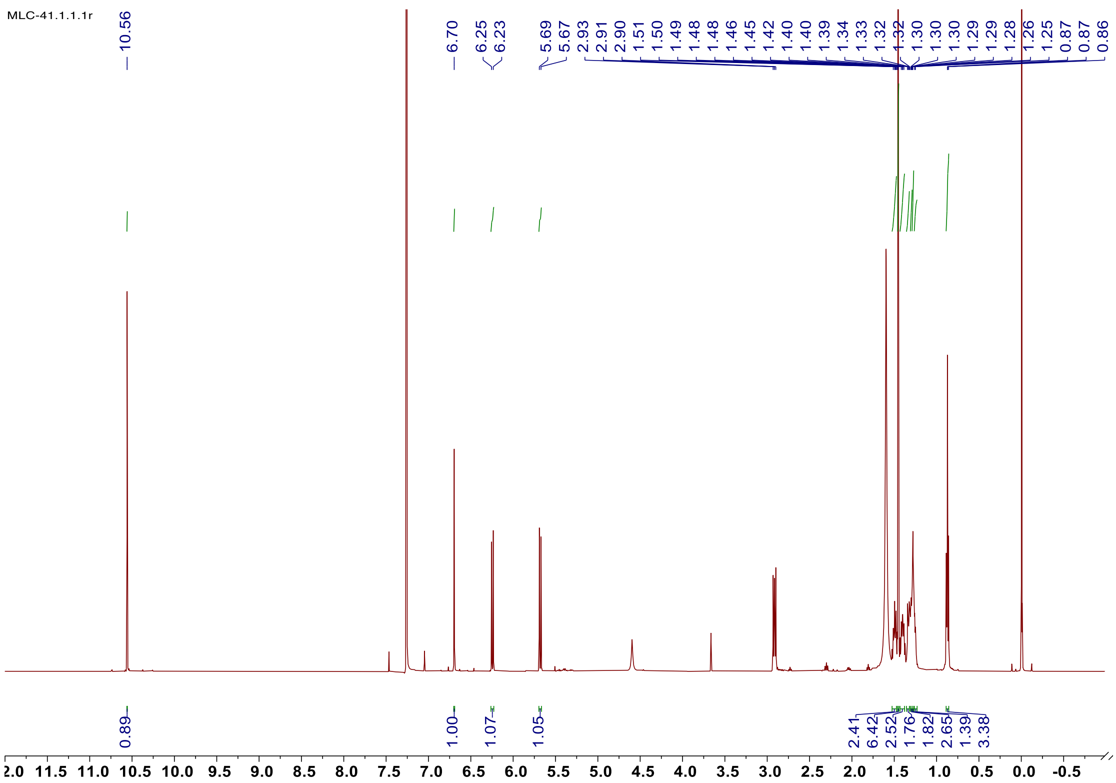

Supplementary Figure 40. ^{1}H NMR spectrum of **6** (600 MHz, $CDCl_3$).

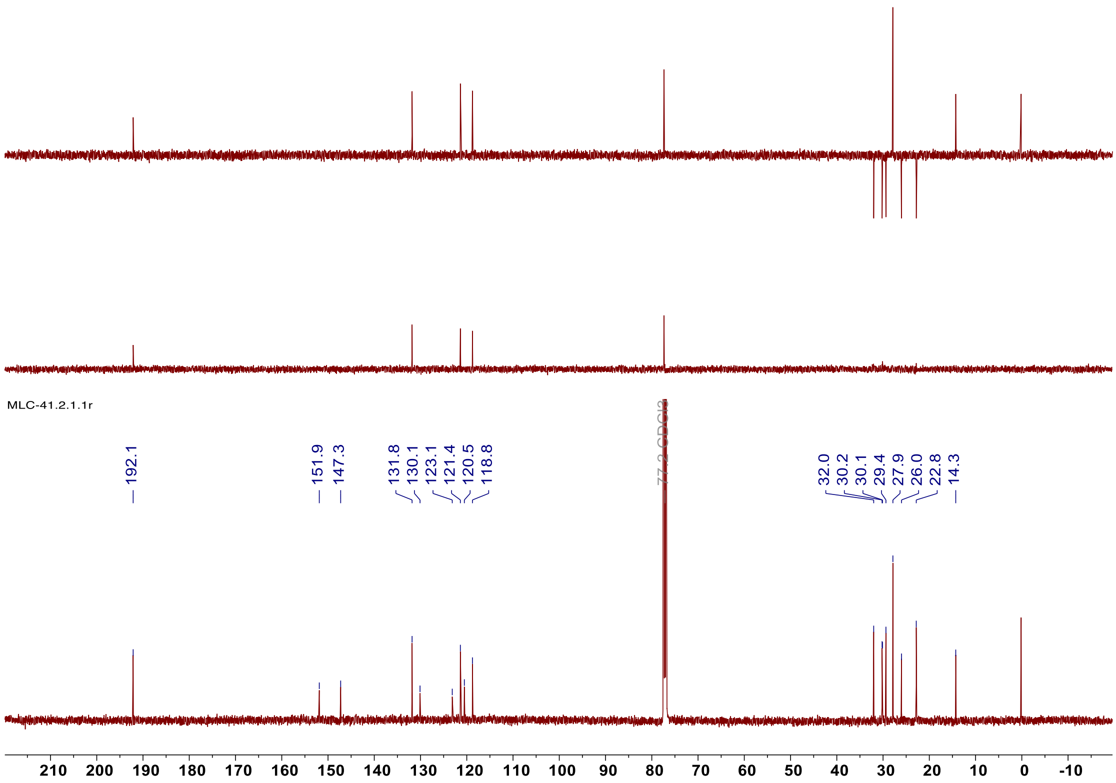

Supplementary Figure 41. ^{13}C and DEPT NMR spectra of **6** (150 MHz, $CDCl_3$)

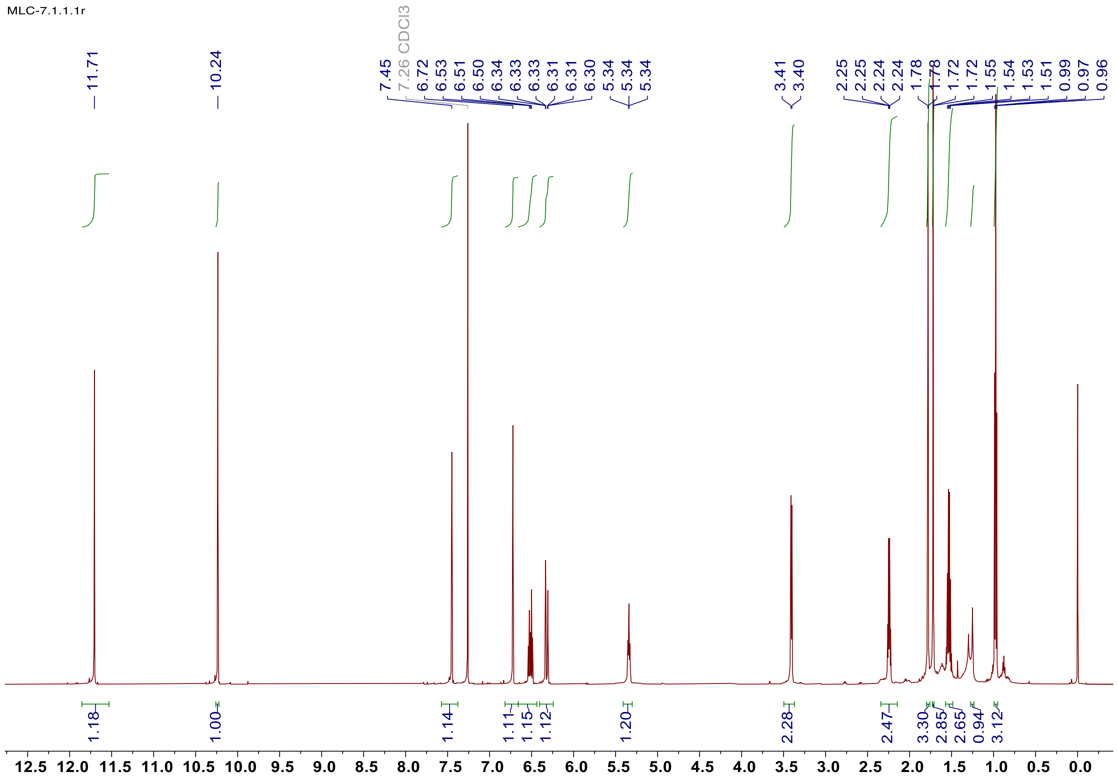

Supplementary Figure 42. ^{1}H NMR spectrum of **7** (600 MHz, $CDCl_3$).

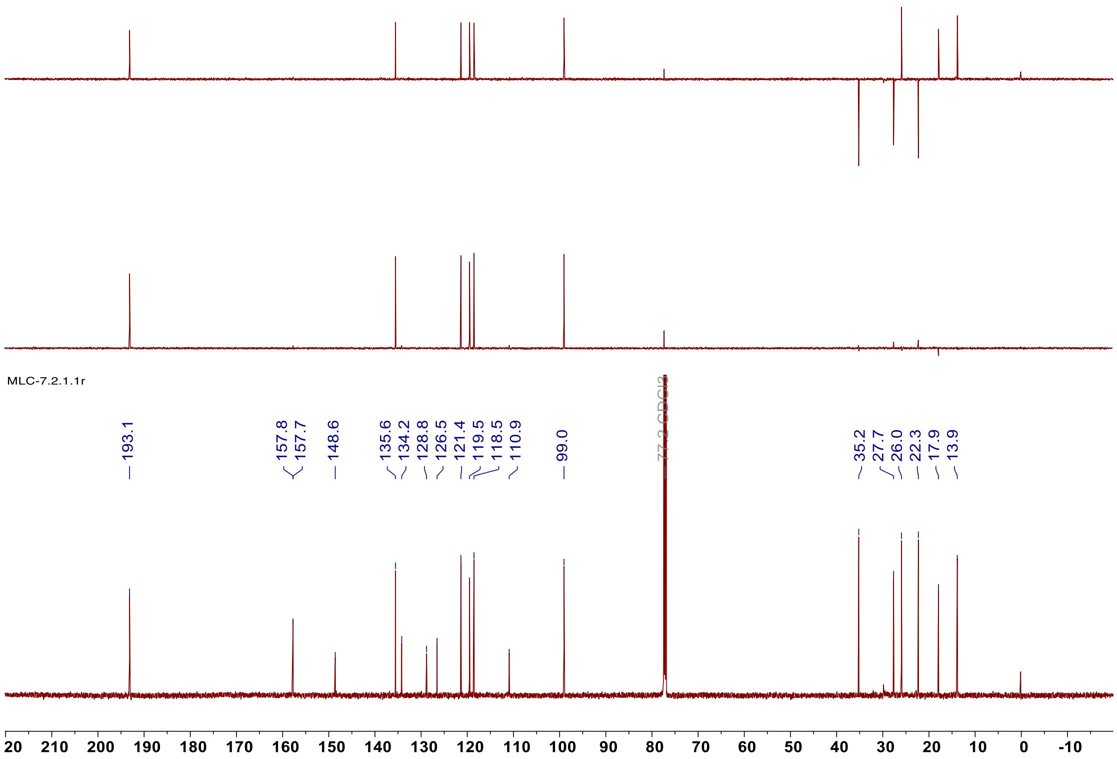

Supplementary Figure 43. ^{13}C NMR spectra of **7** (150 MHz, $CDCl_3$).

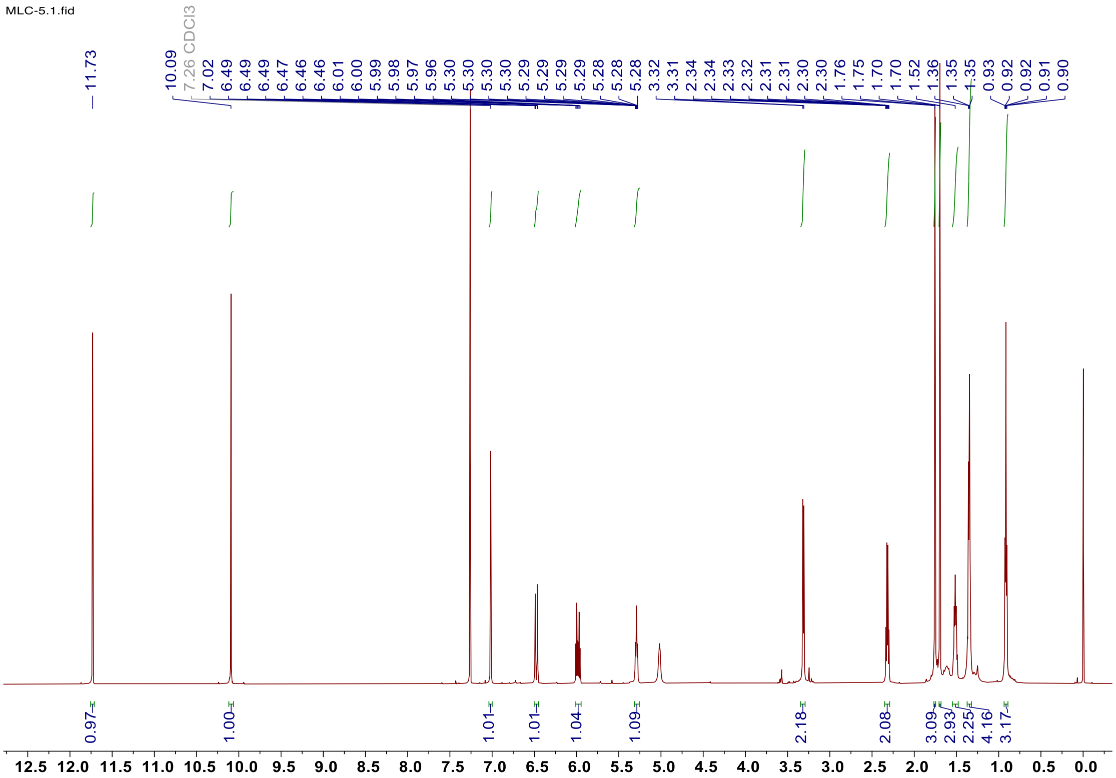

Supplementary Figure 44. ^{1}H NMR spectrum of **8** (600 MHz, $CDCl_3$).

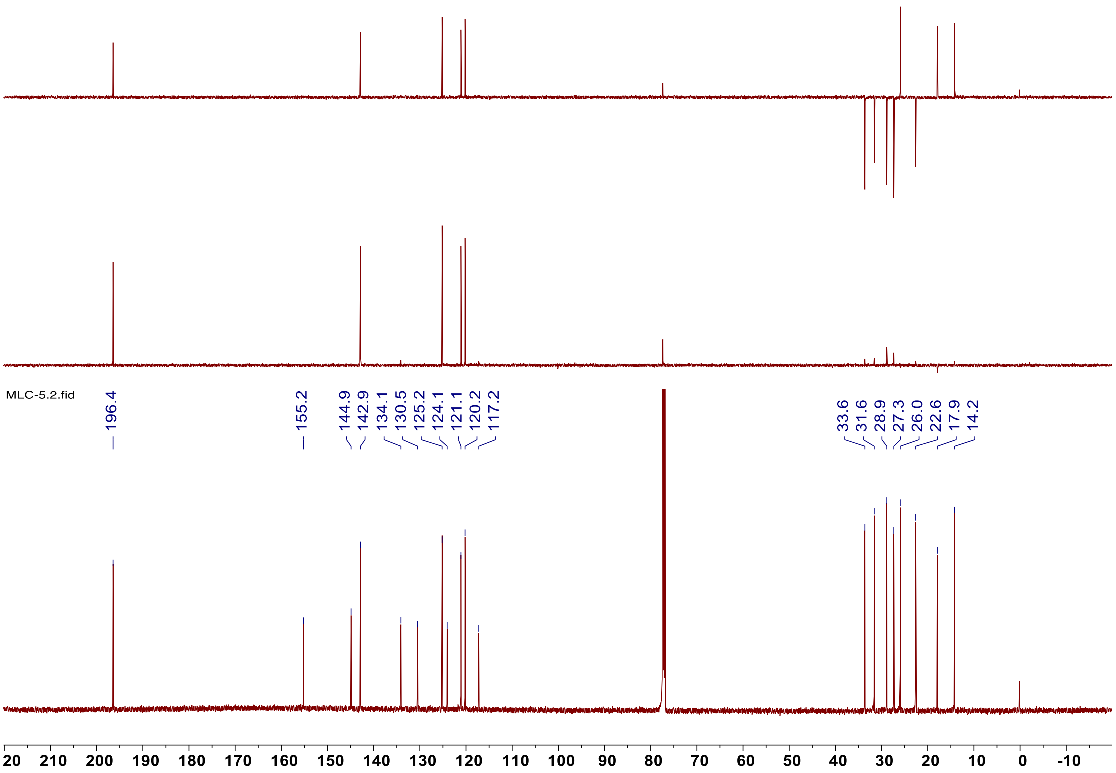

Supplementary Figure 45. ^{13}C NMR spectra of **8** (150 MHz, $CDCl_3$).

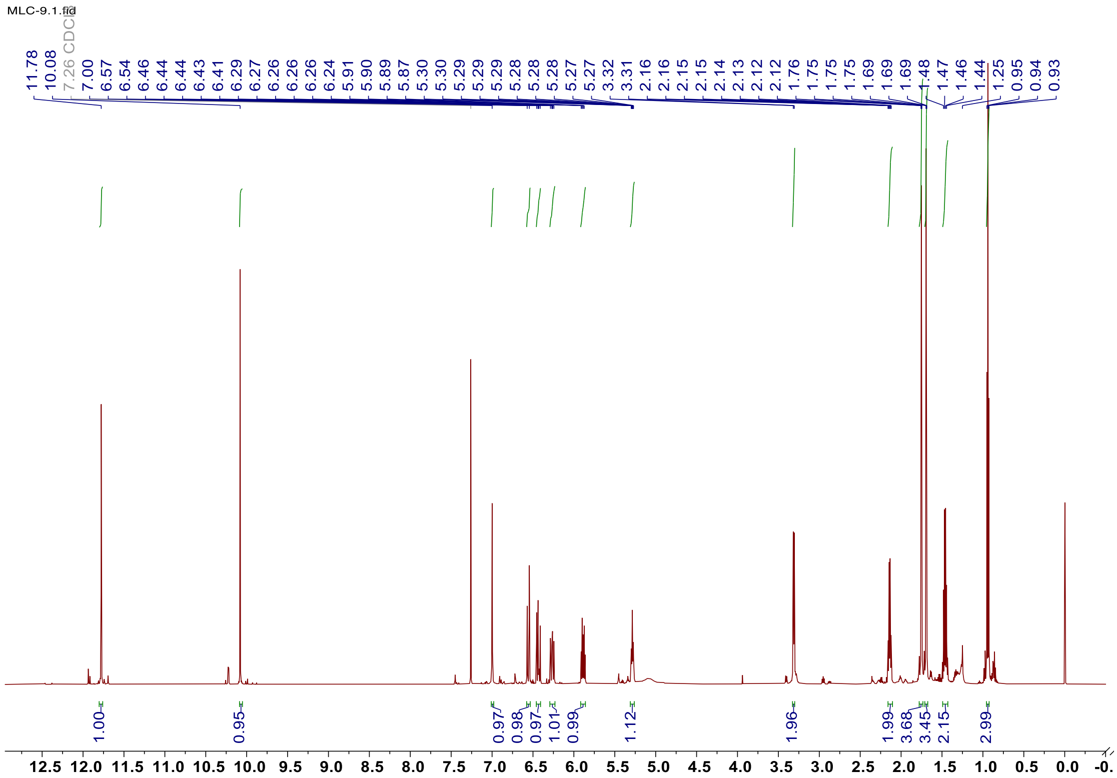

Supplementary Figure 46. ^{1}H NMR spectrum of **9** (600 MHz, $CDCl_3$).

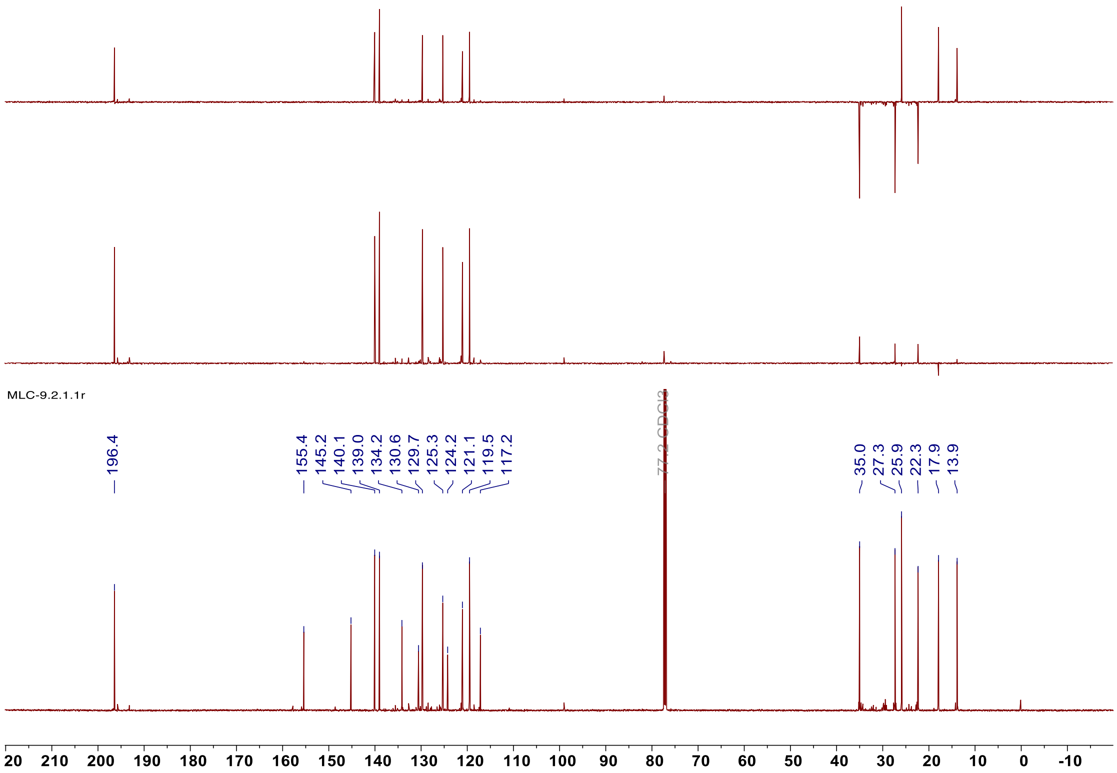

Supplementary Figure 47. ^{13}C NMR spectra of **9** (150 MHz, $CDCl_3$).

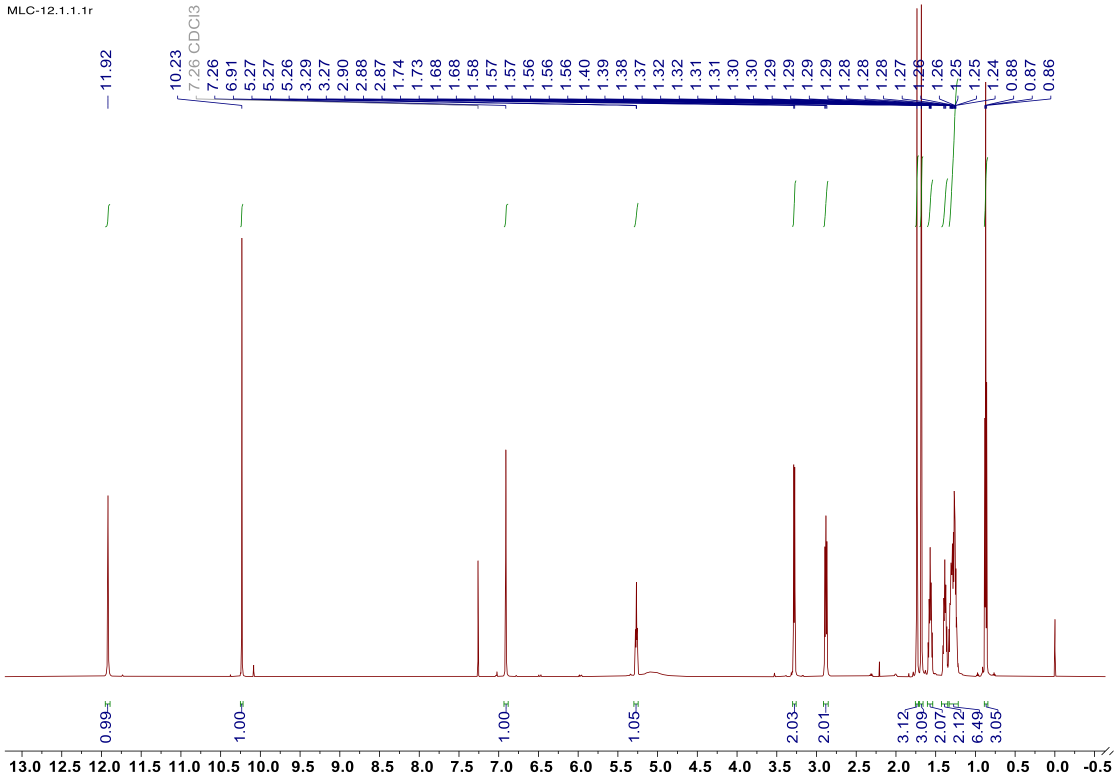

Supplementary Figure 48. ^{1}H NMR spectrum of **10** (600 MHz, $CDCl_3$).

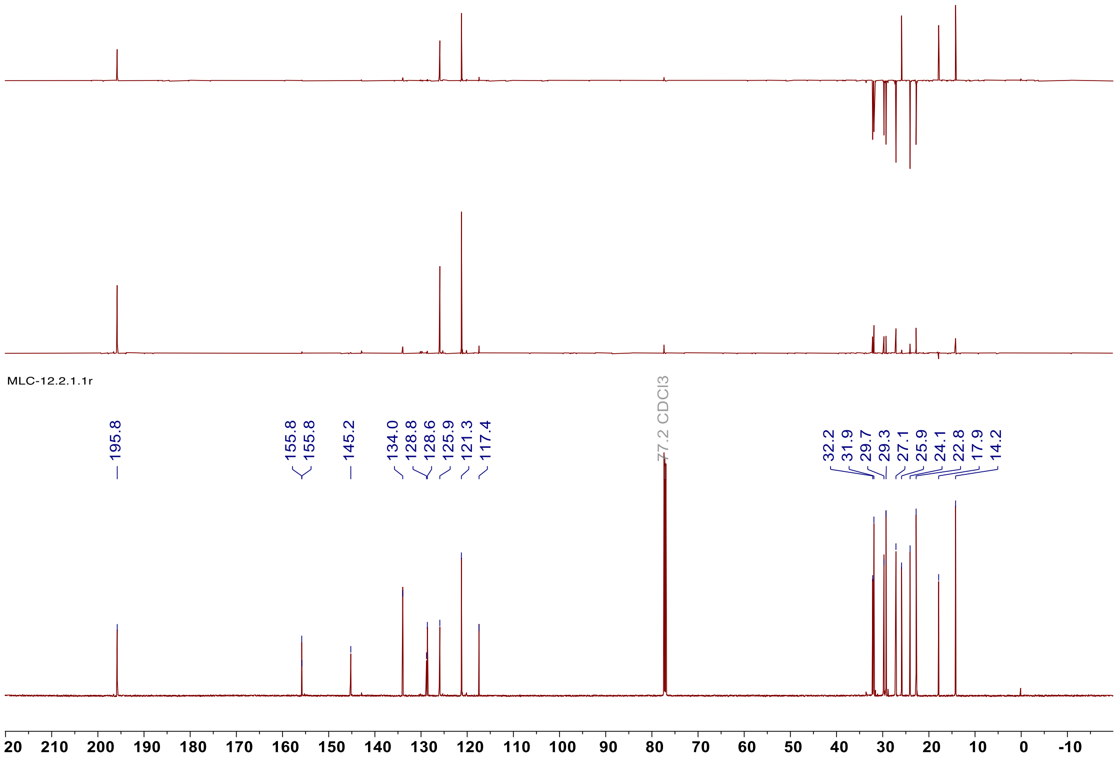

Supplementary Figure 49. ^{13}C NMR spectra of **10** (150 MHz, $CDCl_3$).

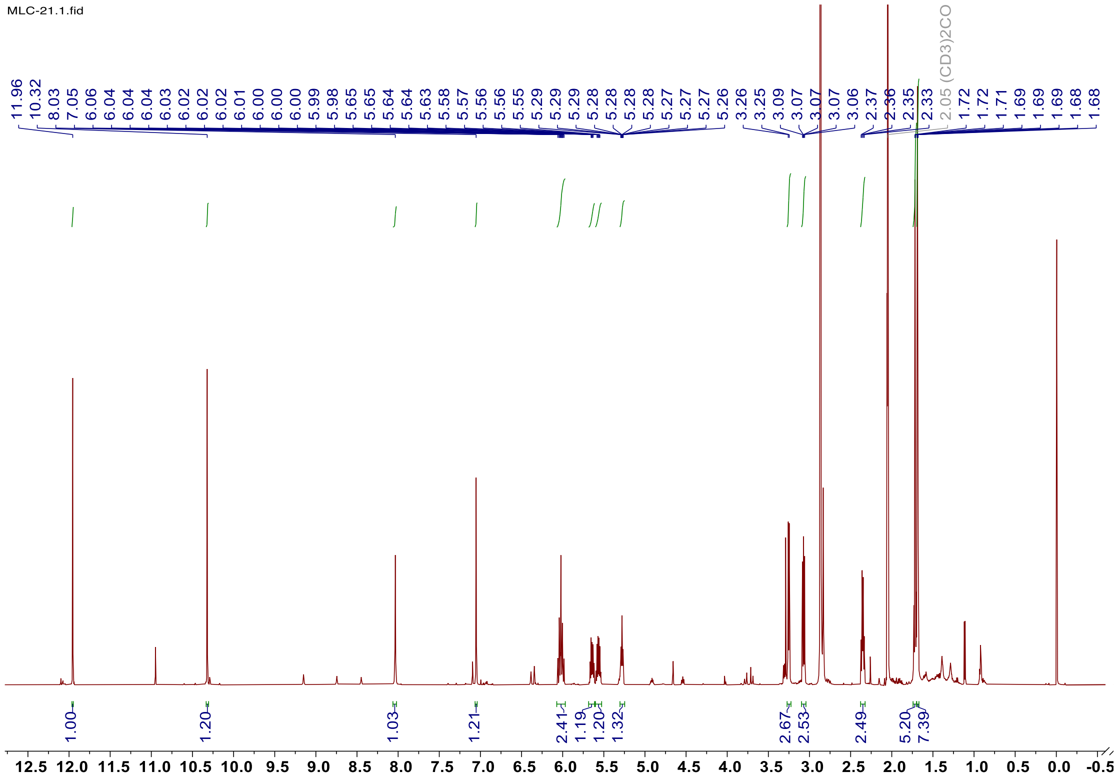

Supplementary Figure 50. ^{1}H NMR spectrum of **11** (600 MHz, acetone-*d*6).

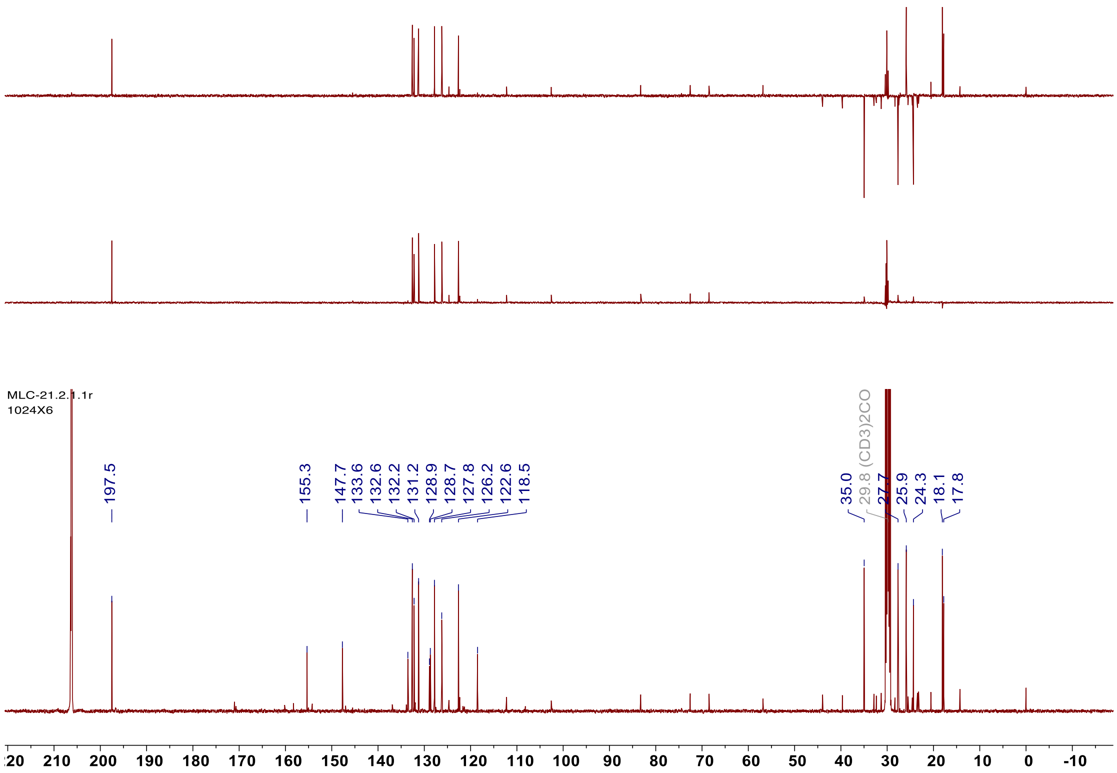

Supplementary Figure 51. ^{13}C NMR spectra of **11** (150 MHz, acetone-*d*6).

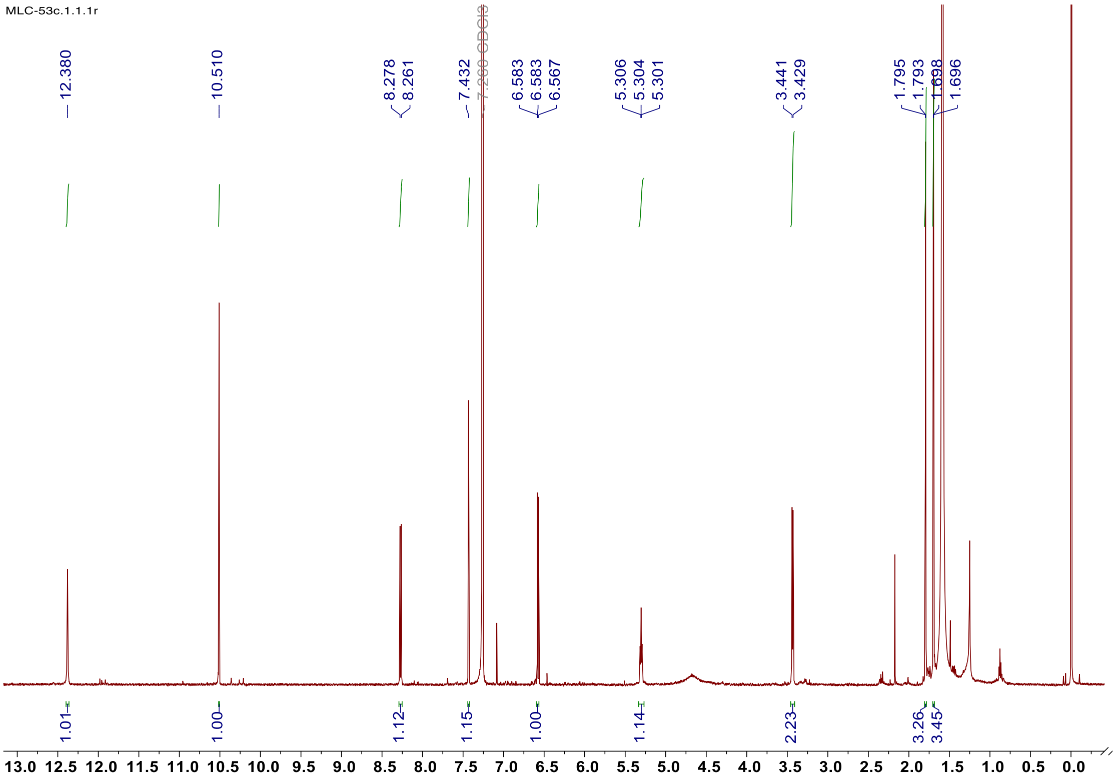

Supplementary Figure 52. ^{1}H NMR spectrum of **12** (600 MHz, $CDCl_3$).

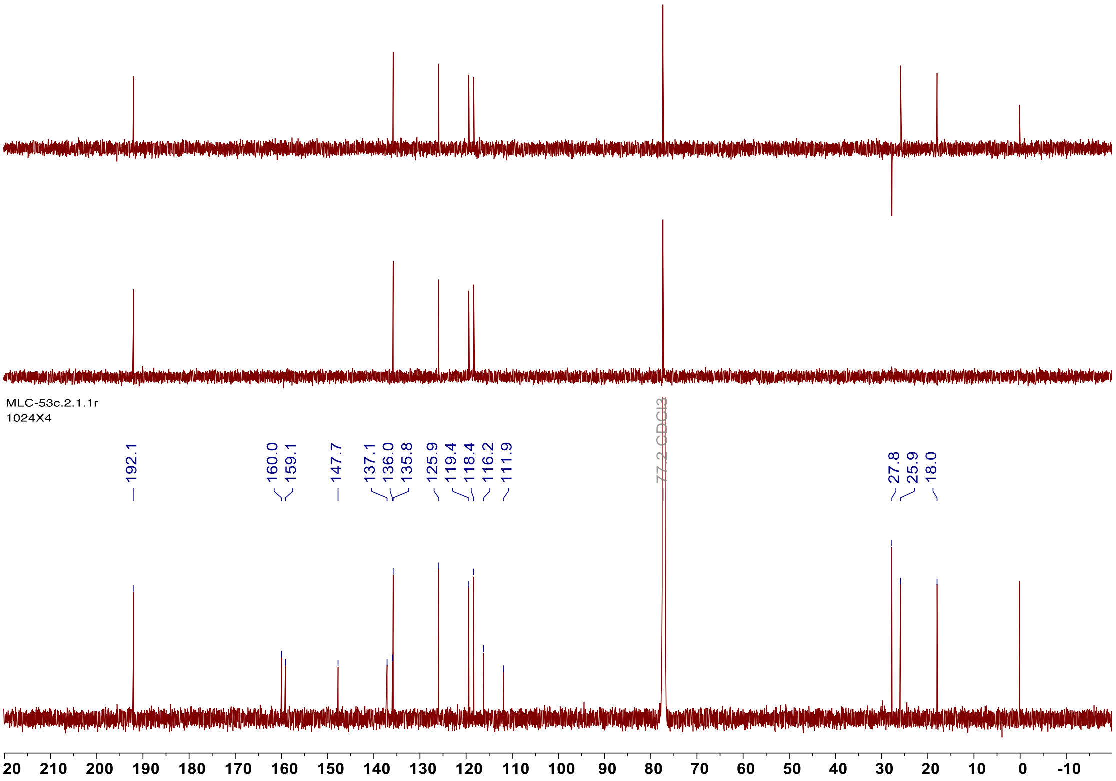

Supplementary Figure 53. ^{13}C NMR spectra of **12** (150 MHz, $CDCl_3$).

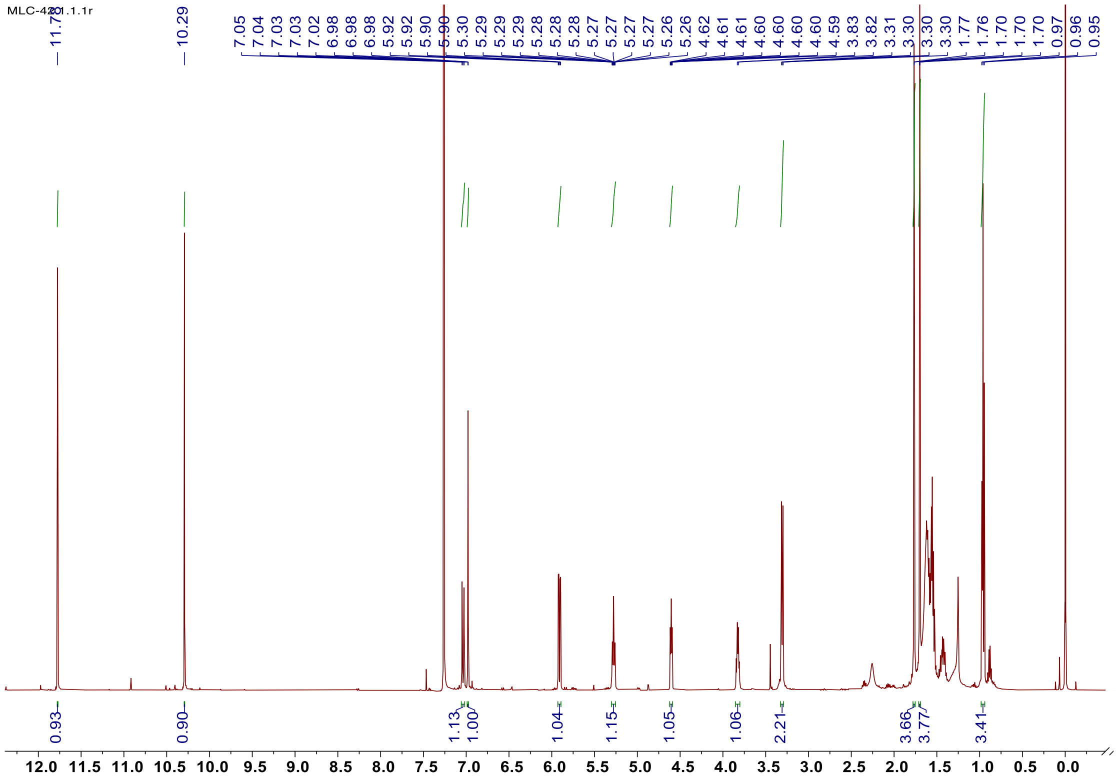

Supplementary Figure 54. ^{1}H NMR spectrum of **13** (600 MHz, $CDCl_3$).

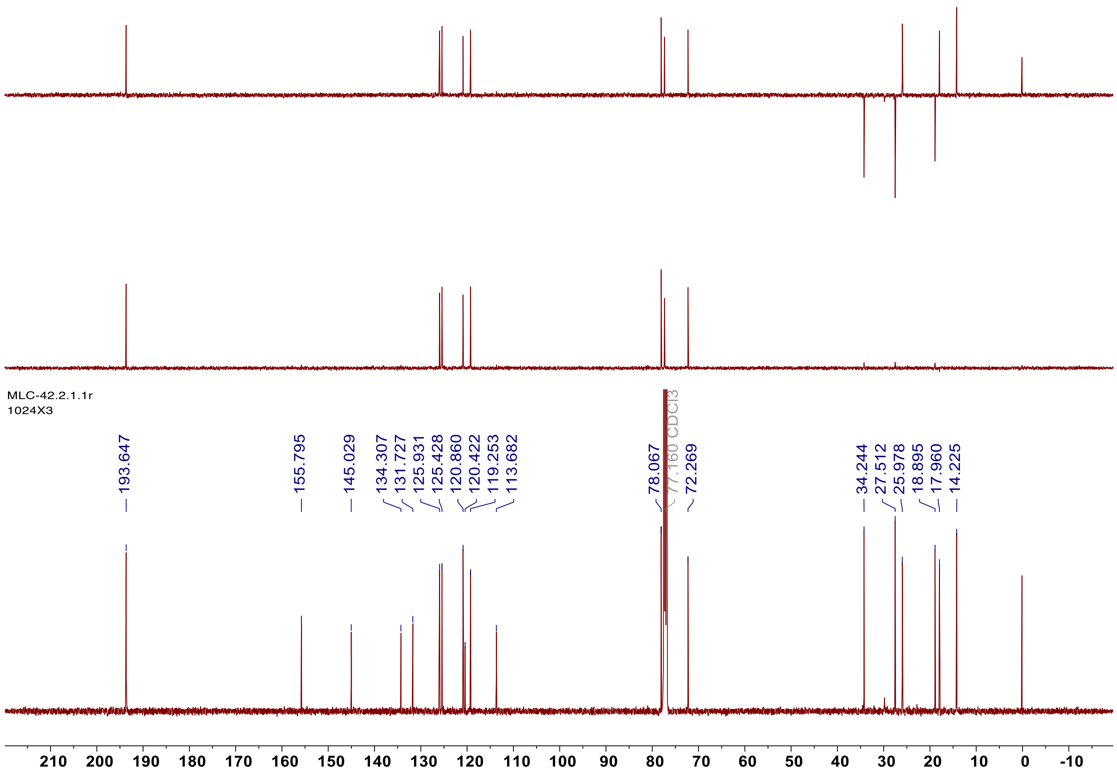

Supplementary Figure 55. ^{13}C NMR spectra of **13** (150 MHz, $CDCl_3$).

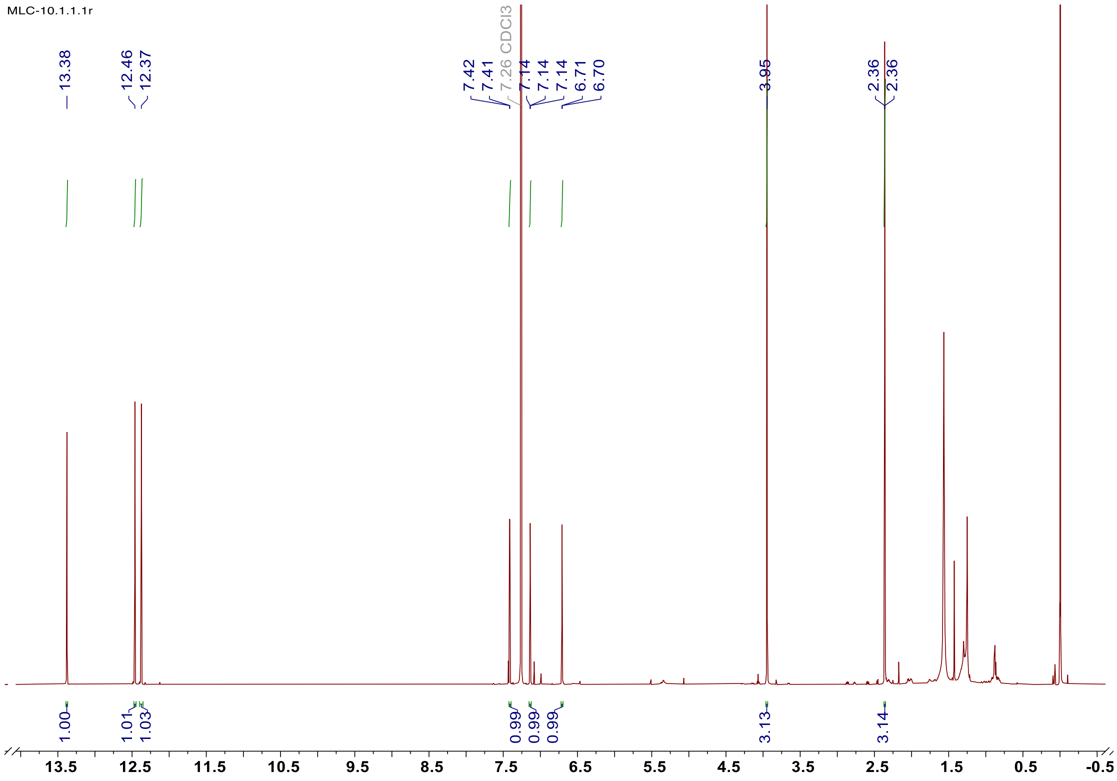

Supplementary Figure 56. ^{1}H NMR spectrum of **14** (600 MHz, $CDCl_3$).

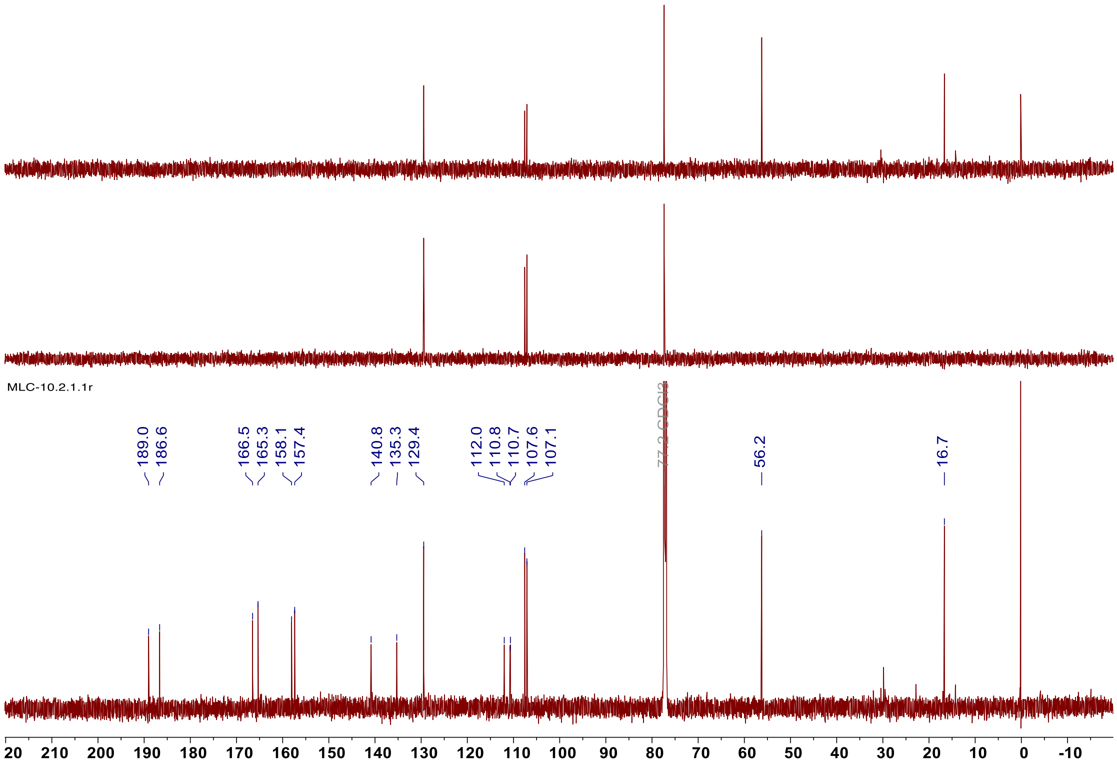

Supplementary Figure 57. ^{13}C NMR spectra of **14** (150 MHz, $CDCl_3$).

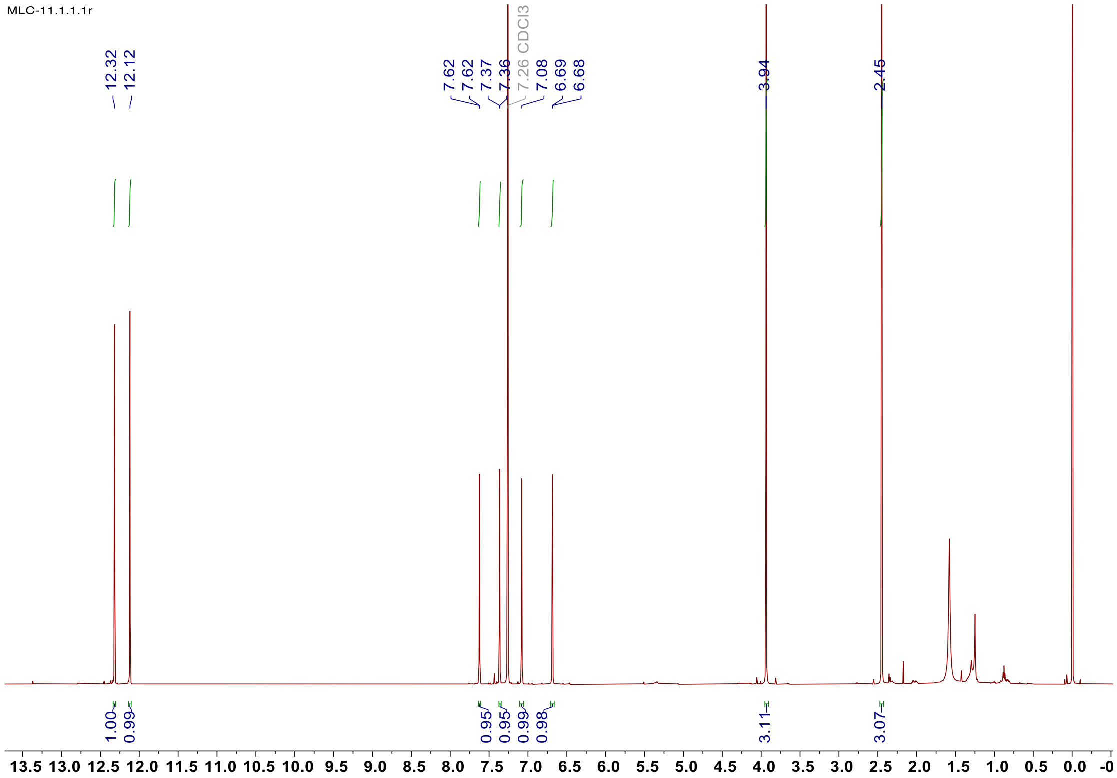

Supplementary Figure 58. ^{1}H NMR spectrum of **15** (600 MHz, $CDCl_3$).

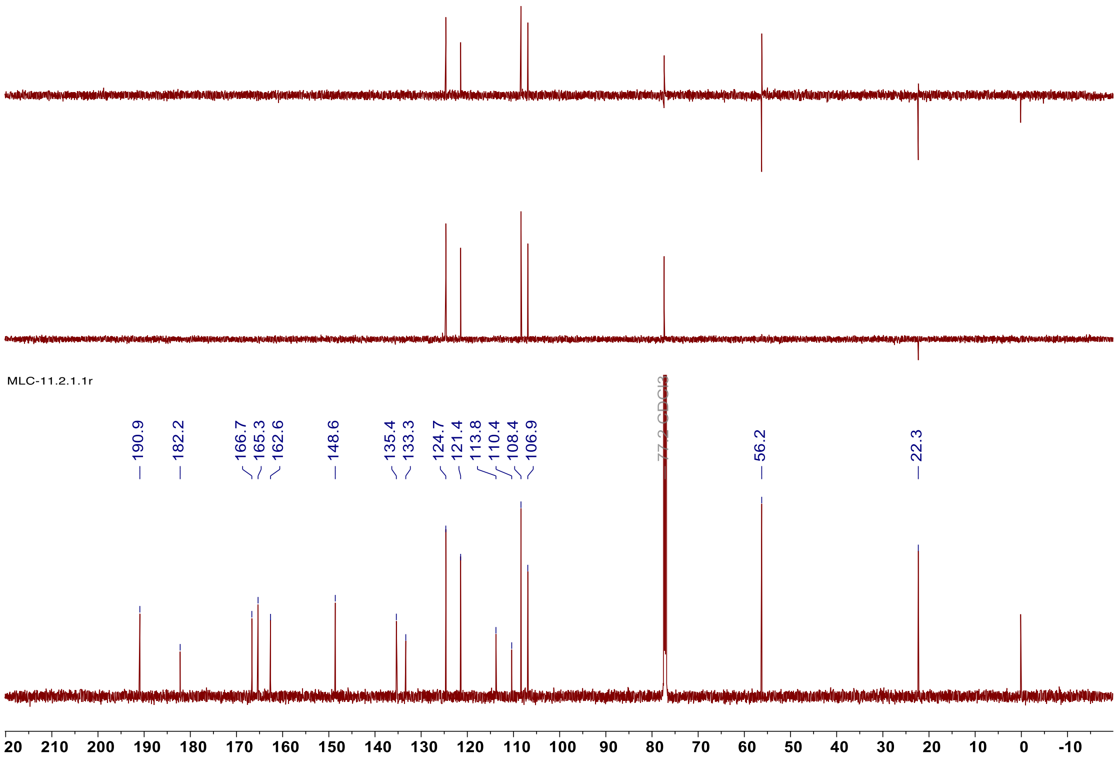

Supplementary Figure 59. ^{13}C NMR spectrum of **15** (150 MHz, $CDCl_3$).

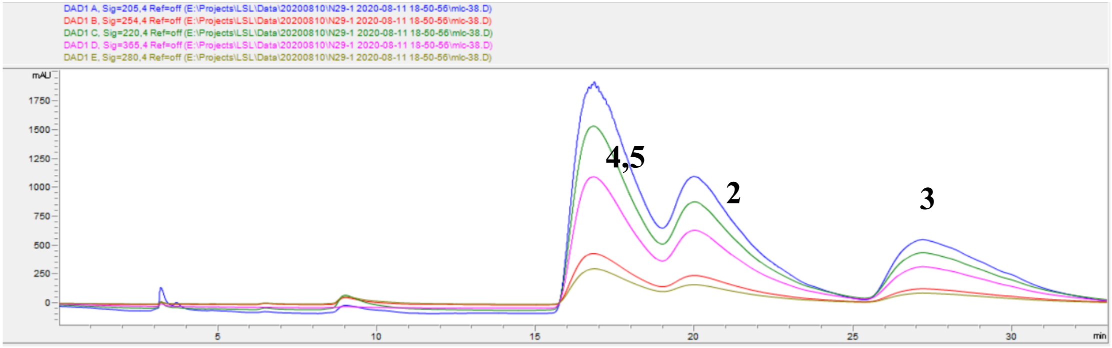

Supplementary Figure 60. Chiral separation of four racemates **2-5** by the column CHIRALPAK AS-H, (flow rate = 1 mL·min^{-1}, *n*-hexane/2-propanol = 97/3).

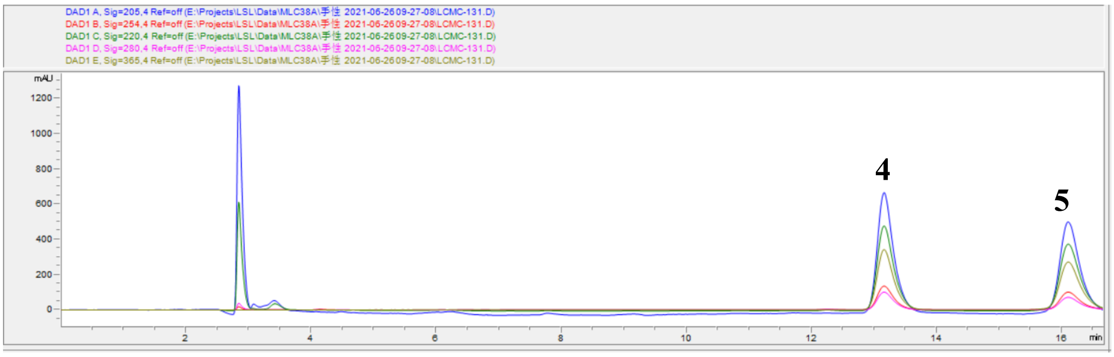

Supplementary Figure 61. Chiral separation of four racemates **4-5** by the column CHIRALPAK AD-H, (flow rate = 1 mL·min^{-1}, *n*-hexane/2-propanol = 94/6).

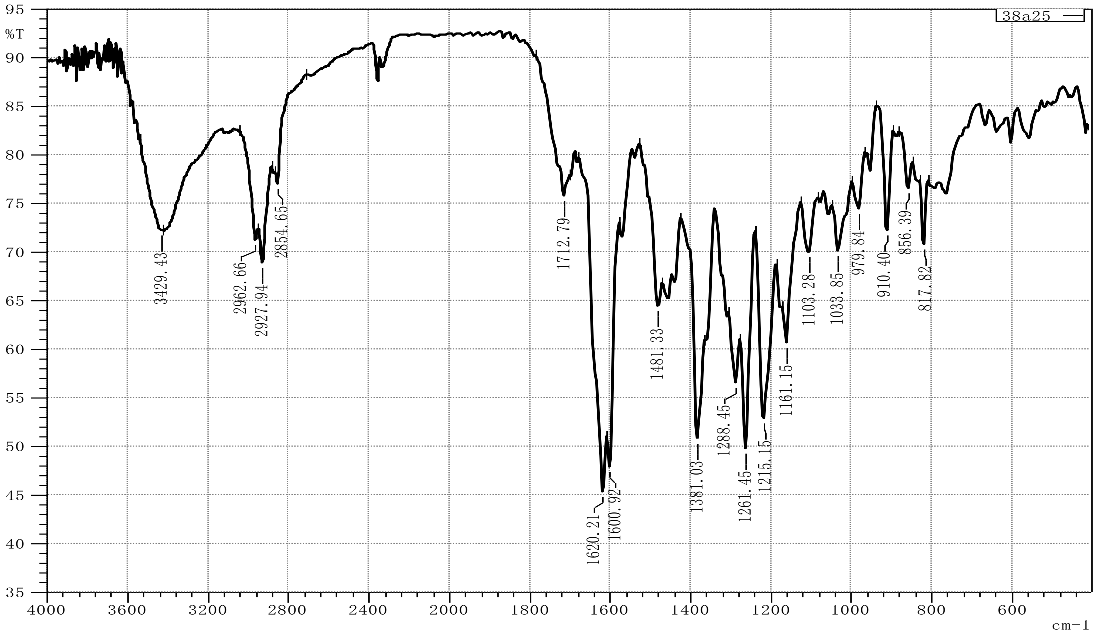

Supplementary Figure 62. IR spectrum of **2**

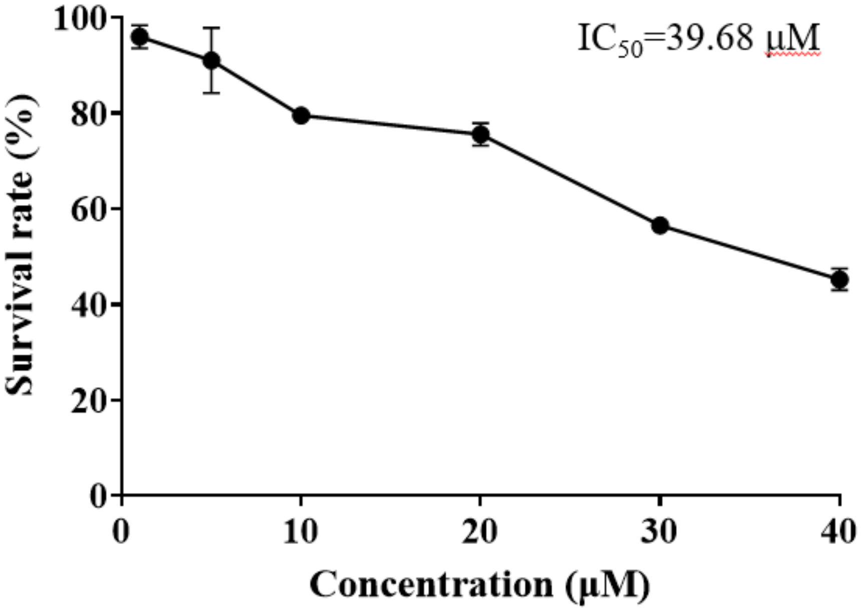

Supplementary Figure 63. Dose-response curve with IC_{50} value for compound **10**

4. Molecular identification result of the fungus

ITS1 primer: TCCGTAGGTGAACCTGCGG

ITS4 primer: TCCTCCGCTTATTGATATGC

>ITS region of *Aspergillus chevalieri*

TGCGGAAGGATCATTACCGAGTGCGGGCCCTCTGGGTCCAACCTCCCATCCGTGTCTATCTGTACCC
TGTTGCTTCGGCGTGGCCACGGCCCGCCGGAGACTAACATTTGAACGCTGTCTGAAGTTTGCAGTC
TGAGTTTTTAGTTAAACAATCGTTAAAACTTTCAACAACGGATCTCTTGGTTCCGGCATCGATGAAG
AACGCAGCGAAATGCGATAATTAATGTGAATTGCAGAATTCAGTGAATCATCGAGTCTTTGAACGCA
CATTGCGCCCCCTGGTATTCCGGGGGGCATGCCTGTCCGAGCGTCATTGCTGCCCTCAAGCACGGCT
TGTGTGTTGGGCTTCCGTCCCTGGCAACGGGGACGGGCCCAAAAGGCAGTGGCGGCACCATGTCT
GGTCCTCGAGCGTATGGGGCTTTGTCACCCGCTCCCGTAGGTCCAGCTGGCAGCTAGCCTCGCAAC
CAATCTTTTTAACCAGGTTGACCTCGGATCAGGTAGGGATACCCGCTGAACTTAAGCATATCATA
